# Supplementary material for: The microeconomics of abortion: A scoping review and analysis of the economic consequences for abortion care-seekers
Source: PLoS One. 2021 Jun 9;16(6):e0252005. doi: 10.1371/journal.pone.0252005 (PMC8189560; doi:10.1371/journal.pone.0252005)
Supplement: S1 Appendix — (DOCX) [file pone.0252005.s001.docx]

## S1 Appendix. Summary of studies reporting microeconomic costs

| **Author, year [country]** | **Aim/objective(s)** | **Population** | **Study type** | **Summary of main findings** |
| --- | --- | --- | --- | --- |
| [1] [Eastern and Southern Africa] | To identify and synthesize the literature on postabortion care (PAC) in eastern and southern Africa with the aim of reporting on the reach, quality, and costs of these services | Varied by studies on PAC, health workers in PAC, incidence and abortion-related service reviews | Systematic review | Medical abortion (MA) is the cheapest method followed by manual vacuum aspiration (MVA) and then dilatation and curettage (D&C).  In Malawi in the absence of complications, researchers estimated MA to cost U$ 12, MVA to cost U$ 19, and D&C to cost U$ 19. In the presence of complications, PAC costs increased to U$ 63 for severe non-surgical complications and U$ 128 for severe surgical complications.  In South Africa, researchers estimated MA to cost U$ 61 and MVA to cost U$ 69. In Swaziland, MA cost U$ 75 and D&C cost U$ 115. In Rwanda, PAC costs were higher if treated in regional health facilities (U$ 239) than in district hospitals (U$ 93) or health centres (U$ 72).  Treatment costs for one instance of post-abortion morbidity represented more than three times the annual per capita health expenditure in Uganda and more than five times that in Ethiopia. Law reform and provision of |
| [2] [Kazakhstan] | To analyze and compare trends in abortion and contraception, women’s attitudes toward abortion, and their perceptions of problems associated with abortion and contraception in Kazakhstan. | Kazakh women aged 15-49 | Cross-sectional descriptive survey | Of the 63% of respondents in who reported that it was difficult to obtain an abortion, and subsequently asked to identify the “main difficulty”, 40% identified “financial problems”, with variations between ethnocultural groups (non-Russified Kazakhs: 27.8%, Russified Kazakhs: 38.2%, Europeans: 47.7%). 54.2% of all respondents perceived financial problems to be associated with obtaining an abortion. |
| [3] [Bangladesh] | To understand where rural women go for induced abortion, their contraceptive practice prior to and after getting pregnant, their reasons for choosing abortion, who makes the decision for abortion, what complications they develop and where they go for treatment for these. | Women who came to one of two rural hospitals (THCs) or four static clinics (H&FWCs) for an abortion-related reason in two rural sub-districts of Bangladesh (Mirsarai and Abhoynagar). [n=143] | Cross-sectional descriptive | Although MR [menstrual regulation] services are supposed to be free of cost in Bangladesh, most providers charge a fee and for medicines. Information on cost (including payments to providers and for medicines) from 62 women: average cost - 618 Taka (range Taka 50-6,000). Average cost was higher for those who aborted at home or at the provider’s place (907 Taka) vs. abortion at a health facility (409 Taka). |
| [4] [Ireland] | To document the experiences and characteristics of women seeking and completing at-home medical termination of pregnancy through online telemedicine in Ireland and Northern Ireland. | Women resident in Republic of Ireland and Northern Ireland who requested at-home medication abortion through online telemedicine [n=5650] | Cross-sectional descriptive | Difficulty affording the €70 donation (34.6%). |
| [5] [United Kingdom] | To examine reasons for seeking abortion services outside the formal healthcare system in Great Britain, where abortion is legally available. | Women resident in England, Scotland, and Wales who requested at-home medication abortion through online telemedicine. [n=209] | Cross-sectional descriptive | Reasons for accessing abortion services outside the formal healthcare setting: lack of eligibility of free NHS [National Health Service] services (13.4%), distance or lack of transport to clinic (9.1%). Women ineligible for free non-emergency NHS services face particular barriers paying for abortion care; these women are either undocumented immigrants, or have been admitted under a visa program and considered visitors to rather than naturalized ordinary residents of Great Britain. For many of these women, the minimum cost of abortion would be approximately £545. |
| (Aiken…2018) [Ireland] | (1) to examine the factors affecting whether women in Ireland choose to access abortion by travelling or by using online telemedicine; and (2) to explore their experiences in accessing care through each pathway | Women (n=38) identified through three organisations: Women on Web, Abortion Support Network, For Reproductive Rights Against Oppression, Sexism and Austerity.  Criteria: aged over 18, had an abortion within 8 years of study, lived in Ireland at time of abortion, had travelled or used telemedicine to access abortion care. | Qualitative in-depth interviews | A respondent outlined the individual cost of seeking abortion-related care while in Ireland (respondent aged 29):  “It costs a thousand Euros to go to England, and that's a staggering amount, particularly if you feel that you can't tell your family, and you can’t look for support elsewhere. Privacy was a factor as well, because there’s this notion of shame in Ireland, of the thought of having to go to a clinic and speak to a nurse. I didn’t feel like I wanted anybody helping me, or comforting me, particularly someone I didn't know. The idea of actually being able to be on your own was appealing to me.” |
| [6] [Ghana] | To explore the abortion experiences of Ghanaian university students | Female undergraduate students at the University of Ghana [n=32] | Qualitative cross-sectional descriptive | Students suggest self-use of pharmaceutical substances is cheaper and more discrete vs. going to a safe medical abortion service provider. |
| [7] [United States] | The impact of TRAP (Targeted Regulation of Abortion Providers) laws on abortion trends and women’s health. | n/a | Systematic review | Findings suggest that TRAP laws increase out-of-pocket costs to women, thus women of lower socioeconomic position may face greater challenges in obtaining abortions. Especially for later trimester abortions which are more restricted and costly than first trimester abortions. TRAP laws may not need to close clinics to have an impact on health and other outcomes: some laws may instead increase service costs or decrease availability of appointment slots, both of which could increase the time it takes for a woman to obtain an abortion. An increase in gestational age at presentation may limit the number of providers willing to perform an abortion and increase out-of-pocket costs to patients. Access-oriented barriers to abortion may introduce special challenges to low-income, young and/ or rural women, as these women may be less able to manage increases in cost and distance. |
| [8] [Ghana] | To explore what direction women perceive gender roles to influence their reproductive choice and care | Data from women with records of recent birth (max 2 years prior to study) (n=90), health staff (n=16), policymakers (n=6) | Qualitative ethnographic study using focus groups and in-depth interviews | At the community, facility, and district facility levels, PAC services are neither provided nor covered under the antenatal/postnatal fee exemption policy under the health insurance scheme. To account for these charges, staff members asked women to pay (US$ 25) for care. |
| [9] [Puerto Rico] | To examine the current practice of abortion and the hurdles women face to obtain this service. | Women attending 10 of the 13 private abortion clinics in Colombia. [n=358] | Cross-sectional descriptive | The cost of an abortion up to 12 weeks of pregnancy (US $200- $300), second trimester abortion (US $700-$800). The average monthly salary is US $1,300.28. The majority of women were dependent on someone else for the money to pay for their abortions. Proportion of women who paid for the procedure themselves: Puerto Rican unmarried teens (11%); Puerto Rican adult women (40%) Dominican adult women (60%). |
| [10] [Uganda] | To perform a comprehensive assessment of the economic burden of induced abortion in Uganda in terms of its costs. | Uganda | Economic estimation | Patients incurred an average of $62 ($46–$183), 73% of the healthcare costs of induced abortion. |
| [11] [Australia] | To describe the context through which medical abortion has become available in Australia since 2013. | Australia | Narrative review | Medicare, the federal government health insurance scheme, has provided a rebate for the cost of a privately provided surgical abortion procedure since 1974. On average this rebate halves the cost of a surgical procedure; prices vary between clinics and increase sharply for pregnancies over twelve weeks. Average cost of surgical abortion under twelve weeks ranges AU $400-$500 after the rebate. For many poor women and those in non-metropolitan locations even this rebated cost is prohibitive, especially when travel costs are also involved. Recent significant development is the launch of the Tabbott Foundation (2015) offers an Australia-wide telephone consultation home medical termination of pregnancy service and for women with a Medicare card the cost is AU $250. A news report at the end of their first week claimed that the foundation had been “so overwhelmed by prospective patients it cannot meet the demand”. |
| [12] [Australia] | A historical examination of the effects of three instances of decriminalization in Australian jurisdictions. | Australia | review | Abortion is provided liberally in Australia, but mostly by private providers. Well-informed women in metropolitan centers with reasonable economic means seeking first trimester abortions are adequately served. While abortions performed at the public hospitals incur little or no cost, access is particularly compromised for Indigenous women in remote communities and others who must travel significant distances to a hospital. |
| [13] [India] | To understand the pathways through which unsafe abortion leads to post-abortion complications and to describe the experiences of women seeking care for post-abortion complications in Madhya Pradesh. | Women of reproductive age who were seeking care for post-abortion complications [n=344] at one of 10 hospitals. | Cross-sectional descriptive survey | (90%) visited one or more other providers before seeking care from one of the study facilities. The median distance travelled per visit was reported as 3.3, 2.3 and 7.3 km, respectively. The most common reasons that women reported for choosing a particular provider for their first visit include proximity or having no alternative. |
| [14] [India] | To compare women with induced abortions with women with spontaneous abortion. | Women of reproductive age who were seeking care for post-abortion complications [n=344] at one of 10 hospitals. | Cross-sectional descriptive survey | The average direct cost of treating induced abortion related complications was almost 85% higher (Rs. 721 or $16) than the cost of treating complications of spontaneous abortion (Rs. 390 or $9). No significant variation in the cost of transportation or clinical tests, but patients with induced abortion complications had significantly higher costs for medicine (P<0.01), the evacuation procedure (P<0.01), and consultancy fees (P<0.05). For both groups of patients, the cost of medicines contributed almost half of the total cost (induced: 47%; spontaneous: 37%). Women who visited other providers had higher expenses than women who went to the hospital first. Even women who were hospitalized for several days after reaching the hospital directly incurred lower costs (induced: Rs. 349; spontaneous: Rs. 218) than women who did not require hospitalization but visited other providers (induced: Rs. 841; spontaneous: Rs. 1169). Many women expressed the burden in terms of managing money and sacrificing regular earnings because of prolonged complications and unsuccessful visits to multiple providers. |
| [15] [India] | To describe young women’s experiences with unwanted pregnancy and abortion, identify their sources of information, knowledge of SRHR and describe their health-seeking behaviours | Young married (n=690) and unmarried women (n=691) (15-24 year-olds) in three rural communities | Cross sectional survey | Of the 23 married women who reported induced abortions, only 4% sought treatment from facilities where such services are virtually free |
| [16] [India] | To understand the socio-economic profile of women seeking abortion services in public health facilities and out of pocket cost accessing abortion services. | Women [n=1036] presenting at 1 of 19 facilities for abortion or PAC services. | Cross-sectional descriptive facility-based survey. | Although abortion services are technically free at public sector facilities, women reported that the indirect average total cost for an abortion at primary health centre was 67 INR (SD = 137 INR; Range: 0–2700), while the same cost was almost three times for women accessed services at urban hospitals. The total indirect cost of accessing abortion services had no significant association with women’s economic profile at primary level facilities. Significant associations observed at the secondary level facilities. Poor women spent more on average (305 INR) than women from middle (188 INR) and rich households (154 INR), when seeking care at urban hospitals. Poor women who visited secondary level urban hospitals spent almost five times more than poor women who visited primary level facilities (305 INR vs 69 INR; p < 0.001), while women from middle (56 INR vs 188 INR; p < 0.001), and rich (75 INR vs 154 INR; p < 0.023), households spent three and two times more respectively. |
| [17] [India] | To explore quality of care received by women seeking abortion services in selected settings in Jharkhand. | Women clients [n=107] who had had an abortion in the two years prior to the study. Key informants [n=16] comprised doctors (who did not provide abortion services), anganwadi workers, ngo representatives, dais (traditional birth attendants) and government providers. Service providers included certified and uncertified practitioners, those from allopathic and traditional systems of medicine, and from government and private facilities. | Cross-sectional qualitative descriptive | A factor influencing choice of provider was cost, reported by over half the clients (63/107). While cost of services did not prevent women from undergoing an abortion, it did determine the choice of facility. Women who could not afford fees charged by private qualified providers sought care from government or unqualified providers. Women clearly linked cost with quality. Women for whom cost was a concern tended to seek care from those perceived to provide cheaper services, even when they had concerns about the provider's technical skills. Cost was less likely to be reported as the leading criterion underlying the choice of a provider. In the tribal block, cost was a key factor; women were unanimous about the need for free, cheap or subsidised medicines, and free services. |
| [18] [United States] | To explore qualitatively the experiences of women who were most affected by the law [HB2]: those who had to travel farther to reach a facility and those desiring medication abortion. | Purposive sample of women [n=20] attending 10 abortion clinics in Texas | Qualitative cross-sectional | Following the passing of the TRAP law House Bill 2 (HB 2) by the Texas legislature, one of the United States’ most restrictive TRAP laws, abortion clinic closures resulted in an increased number of Texas women of reproductive age living farther from an open facility, meaning that more women had to travel longer distances to obtain abortion care. Women described the stress of having to get to unfamiliar cities and coordinate transportation, and extra costs such as staying in a hotel, fuel and transportation. Women reported difficulty with out-of-pocket costs and loss of earning associated with travel, and the need for some women to have to ask for help with these costs. |
| [19] [Benin and Burkina Faso] | To document the means women use to obtain abortions in the capital cities of Benin and Burkina Faso  To learn whether or not use of misoprostol has become an alternative to other methods of abortion and the implications for future practice | Women in Cotonou (n=21) and in Ouagadougou (n=13), including 5 secondary school students | Qualitative in-depth interviews | Six women self-managed their abortions, citing little or no money to access clinics as one of their reasons.  Women reported paying 10,000 CFA [16.18 USD) for general consultations in Benin and a further 15,000-55,000 CFA (24.50-89 USD) for abortion services.  Women in Burkina Faso reported paying 30,000 CFA (48.50 USD) in public health facilities and 60,000 CFA (97 USD) in private facilities for abortion services. |
| [20] [Thailand] | To examine the needs for reproductive services of Burmese migrant workers along the border with Thailand. | Women experiencing any type of early pregnancy loss; traditional midwife trainers [n=50]. | Cross-sectional descriptive | Women had to pay 2300 Baht ($US 56) on average for their hospital bill for post-abortion care. The majority of Burmese women in Thailand earn less than 3000 Baht ($US 73) a month. Induced abortions conducted by trained Thai medical staff at private clinics in regional towns cost 3000-7500 Baht (US$70-$174) depending on the service and gestation of the pregnancy. However, their illegality forces the prices up and there is little follow-up care. Undocumented workers are unable to travel to such clinics, and the need to show documentation and the prohibitive cost mean women rarely access these services. |
| [21] [sub-Saharan Africa] | To document the magnitude of abortion complications in Commonwealth member countries. | Sub-Saharan African Commonwealth member countries | Review | Cost-related issues were mentioned in many of the published articles reviewed for this monograph. Brief descriptions of the cost of obtaining an induced abortion, the average length of stay for women treated for abortion complications, and the time needed to perform an evacuation procedure were among the points cited. |
| [22] [Global] | To examine the changes in policy and health service provision required to make abortions safe. | n/a | review | Where abortion is clandestine and unsafe, women (or their partners and families) are buying drugs and other means of self-induced abortion and/or paying clandestine providers, while public health services and women are paying for the treatment of abortion complications. Costs (economic and social) incurred for unsafe abortions include not only acute care, however, but also the longer-term complications of damage to reproductive organs, pelvic inflammatory disease, and secondary infertility. Costs for families, especially for a woman's existing children, also include those that result from a maternal death. Unsafe abortion situations are characterised by a lack of equity in cost, safety and quality of care. Women may or may not be charged a fee at the point of service, but safety means affordability for the poorest of women as well as for those who can pay. |
| [23] [Global] | To discuss choice and acceptability of medical abortion from the perspective of both women and abortion providers. | n/a | review | Unless the costs of surgical and medical abortions are broadly similar, many women will feel they need to choose the cheaper option, which limits choice. Where the health service is covering the cost, or health insurance companies control treatment options, similarly, clinicians and managers will also tend towards the cheaper option. |
| [24] [United States] | To learn about women’s experiences applying for subsidized insurance and to identify barriers to obtaining insurance or its use for abortion services. | Interviews with [n=39] women who met the eligibility requirements for subsidized insurance programs in Massachusetts. | Qualitative cross-sectional | More than half the women interviewed reported delaying their abortions while they tried to secure subsidized insurance; this caused considerable stress as the women tried to manage symptoms, often without disclosing the pregnancy, and worried about being able to afford a termination if the pregnancy continued too long. Delays can have severe consequences, including delaying care until the second trimester. |
| [25] [Latin America and Caribbean] | To review results from 10 PAC operations research projects conducted in public sector hospitals in seven Latin American countries. | 10 PAC operations research projects conducted in public sector hospitals in seven Latin American countries. | Review of 10 operational research studies | Patient fees and other out-of-pocket expenses are likely to play an important role in whether patients seek professional PAC services when needed. The high cost of services may be a barrier to obtaining clinical services. One of the shortcomings of many PAC cost studies has been a narrow focus on costs to the facility. Facility costs certainly are a key component of sustainable services, but patient fees and other out-of-pocket expenses are likely to play an important role in whether patients seek professional PAC services when needed. As reorganizing services can substantially reduce opportunity costs to the facilities, in some cases administrators have passed these savings on to the patients by reducing fees. In the Peru-Callao study, when the director of the hospital was presented with data documenting the high cost of treatment with SC (average of US$119 per patient) and the potential for marked reductions with MVA provided on an outpatient basis, she cut patient fees in half for ambulatory patients (from approximately US$32 to $16) (Benson et al. 1998). Overall out-of-pocket expenses (including admission, treatment fees, medications and some supplies) declined from US$52.98 before to $37.40 after the intervention. At the time of the 2000 follow-up, clients’ costs averaged $32.75. The hospital was recovering almost 98% of the full cost of providing PAC services at the 2000 follow-up compared with less than half (45%) prior to the intervention. |
| [26] [United States] | To study women's experiences seeking and receiving second-trimester abortion care in two geographically and legislatively different settings to inform ways to improve abortion care access and services. | Women receiving second-trimester services in eight clinics in Iowa, Nebraska, New Jersey, and Pennsylvania. | Mixed methods | Most women reported traveling at least 1 hour to their appointment, with one woman traveling 5 hours. More than half of participants travelled up to 50 miles. Women reported challenges securing transport, childcare, and work leave. Interviewees reported paying between $300 and $1066 for services, with the mean cost of $722. Although 59.6% of women had insurance, over half of participants paid out-of-pocket. Women with insurance reported insufficient coverage, confidentiality issues, or complex processes and delays to obtain coverage. Most interviewees sought financial support from partners, family, friends, and/or abortion funds. |
| [27] [Ireland] | This paper considers abortion tourism in Ireland, both north and south, and how the moral conservatism present in both jurisdictions has impacted on attitudes and access to abortion | Literature on women seeking abortion services in restricted settings. | Literature review and supplemental expert interviews | Women who cannot access abortion in Northern Ireland must travel elsewhere to obtain one and pay as private patients despite being UK taxpayers. The cost of this ranges from £600–£2000, including travel and accommodation. This cost creates a significant burden to women with low incomes, often leading them to borrow from backstreet lenders. The difficulties in obtaining funds can also lead to delays in obtaining an abortion, thereby increasing its cost. Over the last decade, the data indicates an overall decrease in those travelling to England and Wales. It was noted that 6% of respondents had obtained an abortion outside the UK, with anecdotal evidence suggesting that travel to EU countries was less costly than accessing abortion services in the UK. |
| [28] [Colombia] | This study sought to identify the key barriers to legal abortion, and to explore the ways they may work separately and together to delay the receipt of high quality, legal abortion care. | Women who obtained a legal abortion in Bogotá (n=17). | Qualitative: in-depth interviews | Seven participants discovered during the process of obtaining an abortion that not only was abortion legal, but that health insurance companies were legally obligated to cover its cost. Through self-advocacy and the assistance of La Mesa, these seven were able to obtain full coverage, though they had their abortions later than they had desired. Study participants described the representatives of clandestine clinics as trying to lure them with prices cheaper than those charged at well-known, legal establishments. These representatives promised that the abortion would be both quick and easy. |
| [29] [United States] | To estimate directly the responsiveness of abortion demand to county-level variations in travel-cost component of the full cost of abortion services | Abortion providers in Texas | Log-linear regressions using data that were obtained from health facilities on each abortion performed and data on the localities of these facilities | The average travel costs in Texas were:  Overall (254 counties): US$ 14.59  Counties with providers (19): US$ 3.61  Counties without providers (235): US$ 15.48  The average time costs in Texas were:  Overall (254 counties): US$ 0.18  Counties with providers (19): US$ 0.22  Counties without providers (235): US$ 0.18 |
| [30] [Global] | This article makes the case for a political geography of abortion that moves beyond a state-based framework to account for changing patterns of resistance and restriction on abortion. | Review of literature on policies and political action related to abortion. | Literature review | Women who must travel for abortion face numerous barriers, of which political and economic obstacles often loom the largest. Crossing borders for an abortion requires a woman to have a passport and visa to freely leave and enter another country; refugees, asylum seekers, or undocumented migrants often lack this documentation and the money required to obtain it. Crossing large distances for abortion also requires a woman to have substantial financial means, access to transport, access to childcare, and a social support network to facilitate the trip. At the highest point in 2001, eighteen women per day travelled from Ireland to England for abortion; as of 2016, that number had fallen to just under nine per day. This decrease has been attributed to the increase in access to illegal abortion pills through online pro-choice networks. The recognition that abortion pills were being widely accessed, despite the threat of a prison sentence for their use, was instrumental in pushing towards a more permissive approach to early abortions in the lead up to its 2018 abortion referendum. |
| [31] [Global] | To describe barriers to fertility regulation | n/a | Review | “The literature on a consumer’s ability to pay … is virtually nonexistent in the case of abortion” |
| [32] [Canada] | This study aimed to document women's experiences seeking and obtaining abortion services while residing in Yukon Territory, identify financial and personal costs and explore avenues through which services could be improved. | Women who accessed abortion services while residing in Yukon (n=16). | Qualitative: in-depth semi-structured interviews | Transportation challenges are further amplified for women living in remote communities, where there are few or no public transportation options available to travel to Whitehorse. All of the procedures through Whitehorse General Hospital were covered by Yukon's territorial health insurance. Out-of-pocket expenses included taxi fares, intrauterine devices inserted on the day of the procedure, medications (methotrexate/misoprostol) for one woman and hotel and gas for the two participants traveling in from outside of Whitehorse who were later reimbursed after submitting receipts. Indirect costs included missed work, missed classes at school and childcare. |
| [33] [Chile] | To describe a study of the criminalisation of abortion as a human rights violation in Chile. | n/a | Review | Women who had abortions with trained physicians reported widely differing conditions and prices, ranging from several thousand dollars for a safe procedure to two hundred dollars for a less safe one. Doctors reported that price is based on weeks of pregnancy and as the weeks rise so does the price. |
| [34] [Chile] | To highlight the gender and poverty-related discrimination that poor women having abortions face in Chile, and how the law is used to undermine medical confidentiality . | Case review files | Review | The cost of an abortion ranged from US $50 to US $200. Prices depended on the method used, and on whether the provider felt sympathy for the woman. Identified two cases where two women had had an abortion from the same provider, but for very different prices. |
| [35] [Poland] | To describe the economic consequences of the stigmatisation and illegality of abortion and its almost complete removal from public health services in Poland since the late 1980s. | Polish women | Review | The cost of a surgical abortion (D&C) in 2006 ranged PLN1,500–4,000 PLN and the cost of medical abortion from PLN400–1,000. The cost of a surgical abortion exceeded the average monthly income of a Polish citizen. Historically, under state socialism, abortion was accessible and, compared to present prices, affordable. |
| [36] [United States] | This study uses the abortion visit as an opportunity to identify women lacking well-woman care (WWC) and explores factors influencing their ability to obtain WWC after implementation of the Affordable Care Act. | Low-income women seeking abortion services (n=34). | Qualitative: purposive sampling; semi-structured interviews | Most women identified at least one structural barrier to engaging in WWC, centered mainly around three themes: insurance and cost, navigating providers and clinics, and geography and transportation. Many women experienced or perceived instability around insurance coverage. In the 2 years before participating in this study, 15 women (44%) had gained or switched insurance and 9 women (26%) had lost insurance. Accordingly, many women cited insurance disruptions as a barrier to WWC, whether owing to changes in employment, clerical errors, or “aging out” of parental coverage. |
| [37] [Thailand] | The purpose of this descriptive study of women from southern Thailand, who had undergone a recent abortion (spontaneous, therapeutic and unsafe), were to obtain data regarding: pregnancy history; number of abortions and cost of abortion related treatments; abortion complications, impacts and related health care services; reasons for having an unsafe abortion; and, circumstances related to an unsafe abortion. | Women that received abortion services from one of six government hospitals | Convenience sample and questionnaire | For most of the women, in both the unsafe abortion group and the spontaneous/therapeutic abortion group, this was their first abortion. Universal health care coverage was the primary payment option for both groups. The mean length of the hospital stay, among the subjects in the unsafe abortion group, was slightly longer than the mean length of the hospital stay among those in the spontaneous/therapeutic abortion group. The cost of health care treatment was found to be slightly higher among subjects in the unsafe abortion group compared to those in the spontaneous/therapeutic abortion group. This finding was not surprising given that, compared to spontaneous/therapeutic abortions, unsafe abortions often are followed by medical complications |
| [38] [Zambia] | To analyse the details of pathways to care, barriers and delays, and the role of others in influencing these pathways. | Females aged 15-43 years seeking either safe abortion or PAC at University Teachig Hospital, Lusaka [n=112] | Qualitative cross-sectional | Financial costs of seeking an abortion played a role in the timing and complexity of trajectories, as well as the choice of abortion method and provider. The hospital served a large area and finding money for transport was a hurdle. To decrease unnecessary hospital use and encourage use of government primary care facilities in Lusaka, a referral from a satellite health centre reduces registration fee at the hospital from K80 (£11.60) to K10 (£1.20). For poorer women knowing how to navigate the public sector health system in this way made care more affordable but it also added an additional step in their trajectory to the hospital. Illegal, unofficial provider payments are quite frequently expected and paid, and can introduce delays to care-seeking. |
| [39] [Global] | To present a new conceptual framework for studying trajectories to obtaining abortion-related care. | n/a | Review | In contexts where abortion is illegal, access to economic resources and emotional support are critical for accessing a medically supervised abortion in a clandestine clinic. Women may delay care in order to have sufficient funds to pay user fees. Disclosure can be influenced by the need for financial support for the abortion. |
| [40] [United States] | The primary purpose of this paper is to explore abortion patients’ perspectives on abortion regulations. | Women seeking abortion services at three abortion facilities in the U.S (n=20). | Semi-structured interviews | Cost of the procedure ranged from $370 to $1,575. 6/20 traveled more than 60 miles for their abortion appointment(s) and 4/20 traveled more than 100 miles. The cost of travel depended on distance and ranged from $5 to $200. Of those who were traveling from out of town, 3/20 were required to stay overnight. Of these three, two stayed overnight because of the nature of the care they were receiving rather than state regulation. Although 10/20 had some type of public or private insurance, all women paid the full out-of-pocket cost of their abortion . Of the women who were insured, only two women attempted to get coverage for their abortion. The remaining women did not attempt to get coverage because they already suspected their insurance would deny them coverage or because they did not want their insurance provider, employer, or parents to know about their abortion. |
| [41] [anonymized Latin American country] | To identify appropriate channels through which instructions of misoprostol use could be disseminated | Healthcare workers, people receiving care and women in the unnamed community | In-depth interviews and focus group discussions | Physicians were viewed as inappropriate for providing misoprostol, as they might promote more costly procedures. Care-seekers tend not to use physicians due to costs.  A respondent who sought a safe abortion noted:  ‘‘They give you another [drug] and sell it to you for the same price as Cytotec. That is to make you suffer for a while, and nothing else. They don’t give you advice for what you want, so they are very, very bad sources of information.’’ |
| [42] [United States] | Examine the relationship between adolescent pregnancy intention and policies affecting abortion access: mandatory waiting periods, parental involvement laws, and Medicaid funding restrictions. | Data from the Pregnancy Risk Assessment Monitoring System (PRAMS) on women under 18 years of age seeking abortion services in 30 states. | Case-control | Those living in states with either Medicaid funding restrictions or mandatory waiting periods reported higher percentages of both unwanted and mistimed birth compared to minors living in states without these statutes. Subgroup analyses noted additional associations with unintended birth for black teens exposed to Medicaid funding restrictions and for white and Hispanic teens exposed to parental involvement laws. These findings may be explained by difficulties accessing abortion. Among minors who identified as black or Hispanic, who received Medicaid, or who had lower educational achievement, the risk of unintended birth was even higher than among the general adolescent population. Waiting periods that mandated multiple visits varied from other types of waiting periods, further supporting the view that it is the financial and time costs imposed by these statutes that impose may alter pregnancy outcome. |
| [43] [United States] | Examine the impact of the Women's Right to Know Act in Texas (disclosure, waiting period, and surgical center regulations) on number, timing, and cost of abortions and distance traveled. | Reported data for abortions performed in Texas. | Regression analysis | Charges for second-trimester abortions increased by more than $400 (39 percent) between 2001 and 2006. |
| [44] [Moldova] | To present information on the current abortion law, policy  and services in Moldova and describe a project whose aim is to improve the quality of abortion  services, including the introduction of medical abortion through training of service providers  and community education. | Abortion services in Moldova | Review | Abortions were performed free of charge 1955-1998. However, most women (67%) had to make unofficial payments to medical personnel. 1998 regulations stipulated that payment for abortion services in the public sector was required (37–65 lei). Abortion is not covered by health insurance, and the public sector price has officially been raised to 170–250 lei (about US$20), while the average income is 250 lei per month. In the private sector the price for an abortion ranges 300-900 lei (US$25–US$70). There are no officially approved categories of women for whom abortion fees are waived. Since 1994, to reduce the number of complications, the Ministerial Order said that all abortions should be performed in hospitals by obstetrician–gynaecologists. This centralisation of services and their relatively higher cost has reduced the accessibility of abortion. There are difficulties in the organisation of service delivery as most abortions are currently being provided on an inpatient basis, and the fees include payment for a hospital stay. The cost of a medical abortion, including the cost of the pills, is much higher than for vacuum aspiration. Thus, many women will not have access to this method, preferring the cheaper option. |
| [45] [South Africa] | To report whether a significant proportion of women seeking abortion in public sector services would be early enough in pregnancy to be eligible for medical abortion and to investigate the hypothetical acceptability of medical abortion among women, policymakers and providers and whether women would attend for follow-up. | People (n=673) attending abortion services in 8 facilities across three provinces in South Africa | Cross sectional survey | 81% of care-seekers spent approximately 1.25 USD to reach the clinic. The cost (in South African Rands) breakdown per area for travel to seek care was:  Soweto (n=272)  No cost (walk) - 13.2%  <R5 - 2.6%  R5-R10 - 70.2%  R11-R20 - 12.9%  >R20 - 1.1%  Cape Town (n=287)  No cost (walk) - 17.4%,  <R5 - 48.4%  R5-R10 - 18.2%  R11-R20 - 12.2%  >R20 - 3.8%  Philadelphia (n=114)  No cost (walk) - 4.4%  <R5 - 14.0%  R5-R10 - 40.4%  R11-R20 - 29.8%  >R20 - 10.5%  Total (n=673)  No cost (walk) - 13.5%  <R5 - 24.1%  R5-R10 - 42.9%  R11-R20 - 15.5%  >R20 - 3.9% |
| [46] [United States; India] | Evaluate relative differences in direct and total (direct and indirect) costs for medical abortion regimens mifepristone and misoprostol or misoprostol alone. | Modeled data with no specific population. | Modeled data and cost analysis | In the United States, the mifepristone– misoprostol regimen was more expensive, regardless of the cost variation for follow-up visits and suction aspiration procedures. The true direct cost difference between the two regimens ranged from US$38.35 to US$67.35 in Model 1 and from US$57.41 to US$71.41 in Model 2. When indirect costs are included, the differences shrink, ranging from US$6.63 to US$22.37 in Model 1 and from US$17.95 to US$31.95 in Model 2. Accounting for the higher efficacy, the mifepristone– misoprostol regimen significantly reduces the actual excess cost of the mifepristone by at least 73% in Model 1 and by 62% to 78% in Model 2. In India, under Model 1, the direct costs of the misoprostol-alone regimen were US$6.59 higher than the mifepristone– misoprostol regimen (increases by US$2.50 in Model 2). When including indirect cost estimates, this cost difference increases to US$10.64. Other factors besides cost also influence decisions related to treatment regimen. In countries where access to abortion providers is limited and waiting periods for appointments are long, the increased efficacy of the mifepristone– misoprostol regimen would outweigh its more prohibitive costs because of the decreased number of office visits required as compared with a regimen using misoprostol alone. In developing countries, transportation and time away from the home become very important factors when examining the differences between the two regimens. |
| [47] [Europe; France; Germany] | To explore the impact of this controversial technology on abortion rates and practices in Europe, looking first at the EU region as a whole, then examining more closely the politics of RU 486 in France and Germany. | Abortion rates, policies, and practices for each nation state. | Policy analysis from published literature | Depending on how health care is funded, access to Mifegyne may vary as well with a woman’s income and social class. For example, in France, where patients are required to stay in the hospital for three days for a surgical abortion, Mifegyne provides an attractive alternative administered on an out-patient basis. In addition, the procedure is fully covered by national health insurance. In the Netherlands, abortion carries no stigma, and the costs are covered by national insurance, so surgical options trump medical procedures in their cost-benefit analysis. The cost of medical abortions (MA) can exacerbate an underlying budgetary dilemma in the National Health Service (NHS): “If women live in an area where a lot of abortions are requested and the budget is tight, then they are often subjected to restrictions beyond those imposed by law.” Unless a woman has private health insurance or can pay out of pocket, she may confront NHS- imposed limits on her abortion choices. |
| [48] [Romania] | Explore, through individual in-depth interviews, psychosocial antecedents and consequences of the Romanian pronatalist policies banning importation of contraception, prohibiting abortion, and imposing tax on childless couples. | Women seeking abortion services prior to the revolution (n=50). | Qualitative: open-ended interviews | Anxiety was also created by the lack of sufficient money to obtain an abortion. A large amount of money was necessary, usually the equivalent of 2-3 months’ salary. Many couples relied on borrowing money from different persons or on selling goods from their home. |
| [49]  [United Kingdom] | To present results of a review of the sexual health situation in the UK and to outline the challenges remaining in order for the UK to meet ICPD goals by 2015. | UK residents | Review | In 2003, 80% of abortions in England and Wales were funded by the NHS (National Health Service) vs. 67% in 1994. The authors conclude that while women should have the right to choose and pay for an abortion privately, all women who want to have an abortion should be able to access abortion services funded by the NHS, and recommend that as a minimum, at least 90% of abortions should be paid for by the NHS. |
| Dawson 2016 [United Kingdom; United States; Russia; Australia; New Zealand; Canada] | To identify quality studies of abortion services to provide insight into how access to services can be improved in Australia. | United Kingdom (n=14), United States (n=7), Russia (n=2), Australia (n=2), New Zealand (n=3), Canada (n=1) | Systematic review | Interviews with American women accessing a MTOP telemedicine clinic explained that they selected it as it saved them money that they would have had to spend on travel. For some women obtaining a STOP in Australia was very expensive, particularly rural women who in one study said they had borrowed money to cover not only the abortion fee but pay for travel, accommodation and additional childcare costs. |
| [50] [Global] | Summarises the results of a literature review on the subject of unwanted pregnancy and induced abortion among women living with HIV/AIDS. | Women living with HIV/AIDS | Review | Cited studies. Zimbabwe - HIV-positive women may be ready to end childbearing, but often cannot put that decision into practice because they lack control over contraception and access to abortion, among other reasons due to the cost. Thailand, 3/30 women had abandoned attempts to obtain a termination because of the costs involved. |
| [51] [United States] | To investigate how Medicaid staff in 17 states responded to inquiries about coverage for abortion in the few circumstances that qualify for federal Medicaid funding. | Medicaid staff [n=23] in 17 states. | Cross-sectional | When asked, respondents who did not have full information about the availability of abortion coverage, the process for obtaining it, or the associated costs, suggested one of three sources for further information: the physician providing the abortion (39%), the state Medicaid website (17%), or other Medicaid offices or staff (13%). |
| [52] [United States] | To answer the following questions: 1) What do women know about the cost of abortion and the availability of Medicaid coverage for abortion? 2) Where do women obtain this information? and 3) What are women’s experiences paying for care? | Low-income women over the age of 18 who had an abortion within the past two years within one of the four study states (n=98). | Qualitative study: semi-structured interview | Women believed that obtaining an abortion is expensive and that the procedure must be paid for out-of-pocket at considerable personal costs. Women’s experiences paying for abortion care varied widely and were almost entirely dependent on their home state’s policies regarding Medicaid coverage. Insured women residing in Massachusetts, New York, and Oregon largely described a straightforward payment process. Most women in these states who enrolled or were able to enroll in insurance at the time of their abortion reported that they would not have had the resources to pay for their care otherwise. Women living in Arizona and Florida, states where Medicaid coverage is largely not available, and women who were unable to access Medicaid coverage in Massachusetts, Oregon, or New York, turned to a number of different resources to pay for care, including drawing from their own resources and borrowing money. Some had savings, most had to wait for their paychecks, work additional hours, juggle bills, cut back on personal and household necessities (usually food), take out loans, use credit cards, and/or sell personal possessions to gather the necessary funds for their abortion. |
| [53] [United States] | Study explores low-income women’s experiences accessing abortion in Massachusetts. | Low-income women who received abortion services in Massachusetts (n=27). | Qualitative: in-depth interviews | 74% of women interviewed had public insurance at the time of their abortion. Participants who used their public insurance to cover their care described the process as easy and straightforward. Two insured women paid out of pocket because they feared a loss of conﬁdentiality if they used their insurance. 33% of women paid out of pocket for their abortion care. Most commonly, uninsured women reported that they: 1) were in the lag period between applying for insurance coverage and being approved, 2) faced challenges navigating initial enrollment procedures and remained uninsured, or 3) had difﬁculty recertifying eligibility for insurance and therefore lost coverage. Uninsured participants often said they delayed their procedure while trying unsuccessfully to initiate or reinstate public insurance that would cover the procedure. The 33% of women who paid out of pocket for care reported that it was difﬁcult to ﬁnd the money they needed. Most of the low-income women in this study described having access to timely, conveniently located, affordable, and acceptable abortion care. Some Massachusetts policies likely contributed to this ﬁnding, including state health care reform, which lead to increases in the already high rate of insurance coverage in the state, and comprehensive abortion coverage in almost all public and subsidized insurance plans. |
| [54] [Mexico] | To identify the perceptions and opinions of people who provide abortion services in Mexico City, three years after implementation of elective abortion legal reforms. | Key informants: clinic health providers [n=10], hospital health providers [n=9] | Cross-sectional qualitative | Participants identified the expenses incurred by women travelling from elsewhere in Mexico to Mexico City as an implication of the exceptionality of the federal District law. |
| [55] [Brazil] | To examine equity in health and health care in Brazil, examining unjust disparities between women and men, and between women from different social strata, with a focus on services for contraception, abortion and pregnancy. | Brazil | Review | Safe abortions are only provided in the private sector illegally; they are prohibitively expensive for poorer women but affordable for those with a higher income. |
| [56] [United States] | 31 family planning clinic sites in rural Washington State were surveyed about their sponsorship, stafﬁng, service provision and population coverage. | Family Planning clinics in rural Washington (n=31). | Cross-sectional survey | Only one clinic provided abortions onsite; the rest referred their clients elsewhere. Respondents for these clinics estimated that women seeking an abortion traveled from 20 to 200 miles each way to obtain an abortion, with an average oneway driving distance of 68 miles. |
| [57] [Australia] | Identify factors that New South Wales (NSW) rural women experience in relation to their ability to access an abortion service and follow-up care. | Rural clinics in NSW (n=7) and women who sought abortion services at those clinics (n=13). | Qualitative: Surveys and interviews | Only one women’s health clinic provides financial support; between 2007 and 2012, 138 women were loaned money with an average individual loan of $450.00. Cost, transport/lack of public transport, distance, lack of services in local area and shortage of rural General practitioners were identified as factors that influenced access to an abortion and follow-up care. Expenses beyond the clinic fee included overnight accommodation, petrol/train/airfare costs, taking time off work (for woman and their support person) and childcare. |
| [58] [Multiple: USA, Canada, Australia, New Zealand, France, Norway, Sweden, Northern Ireland, Norway, UK] | To identify the factors that facilitate and hinder access to abortion services for women in developed countries in relation to first-trimester abortions. | n/a | Systematic Review | The direct and indirect costs of travel – including time away from work or studies; extended arrangements for child care; transport, accommodation and cost of meals; poor continuity of care and significant time away from home – were identified in four studies. The cost of abortion procedures was identified as a barrier in four studies. Almost 20% of Canadian women who accessed an abortion clinic reported that the fees were too high. In the USA, hospital-based abortions cost around six times that of non-hospital abortions and increase sharply beyond a gestational age of 12 weeks. Almost 75% of women self-fund their abortions. Research undertaken in 15 USA states revealed that in only two states were 97% of submitted claims funded, and women with low incomes experienced significant challenges to access affordable and timely care. Women who qualify for Medicaid have delays in reimbursement, which sometimes prohibits them from accessing abortion. Delays in accessing resulted in an inability to access an abortion; later abortions for some women; and inability to access a medical abortion. In the USA in 2008, medical abortion at 10 weeks was reported to be more expensive than surgical abortion except in facilities with smaller caseloads. Another study found some women choose to travel for anonymity, lower fees or to access a surgical abortion which might not be available locally. |
| [59] [Australia] | Identify factors that women in rural New South Wales (NSW) experience in accessing abortion services and suggestions about how rural women could be better supported when seeking access to an abortion service. | Women who sought access to abortion while living in a rural part of NSW (n=13). | Qualitative: in-depth interviews | Logistics arrangements included managing expenses related to organising early morning departure, child care, borrowing a car, seeking finance, asking a support person to drive them to and from the clinic, overnight accommodation, petrol/train/airfare costs and taking time off work for the woman or the support person. Participants travelled 1–9 hours one way to reach a clinic and five women required overnight accommodation. Not all women mentioned problems with money but several did. Some women’s partners paid the abortion fee, even if they were separated. Some women commented that the abortion cost, whilst expensive in the short term, was not as expensive as raising a child. Many women borrowed money to help with petrol, abortion fees or accommodation. |
| [60] [United States] | Synthesis of published literature and current practices for adolescent abortion care. | Literature on national abortion statistics and evidence for adolescents. | Literature review | The availability of affordable abortion services determines if a young woman can obtain an abortion at all and how quickly. Compared with adults, barriers to care may particularly affect adolescents with limited resources. Obtaining an abortion may require significant travel because most counties in the United States (more than 80%) have no abortion provider. In most states, abortion is not covered by state Medicaid programs and poor teens may have difficulty funding the cost of care. Delays experienced by adolescents lead to increased costs and risk, as later abortions are more technically difficult and expensive. |
| [61] [India] | Examination of the political economy of abortion care in India by reviewing cost and expenditure patterns for abortion care in India. | Literature/database review | Literature/database review | First trimester abortion is mostly available for Rs.500–1000 and second trimester abortion for Rs.2000–3000. Overall in the six states, the average charges for an induced abortion were Rs.615. This is equivalent to more than three weeks of average per capita income for all- India. The overall charges in the public sector averaged Rs.115 (or four days of per capita income) and in the private sector Rs.801 (or 30 days of per capita income). A 1987 study on health expenditures, which included abortion, found that the mean expenditure for an induced abortion was Rs.300, of which 41% went to the doctor and hospital and 36% for medicines and tonics. The share of abortion expenditure in total out-of- pocket household health expenditure was 0.21%. In two recent studies, the share of abortion in total out-of-pocket household health expenditure was 0.16% in 2000 and 0.28% in 2001, respectively. Analysis of expenditure data shows that women have to spend substantial amounts to access both private and public abortion services. Public abortion services until recently were free of charge even though women reported out-of- pocket expenses (usually non-medical expenses like travel or prescription drugs). At present, abortion services in the public sector are free only if the woman or her husband accepts some form of contraception, usually sterilisation or an IUD, after the abortion. |
| [62] [India] | To synthesize findings and reports from the Abortion Assessment Project – India | 380 facilities in six states in India | Review of studies: policy reviews, multicentred facility surveys, 8 qualitative studies, community-based studies | Out of pocket cost of abortions in Maharashtra and Tamil Nadil were Rs. 1220 and Rs. 950 respectively. |
| [63] [Latin America and Caribbean) | Present evidence of medical abortions’ (MA) contributions to reduced complications, describe strategies to enhance safe MA, and highlight existing barriers to access in Latin America and Caribbean (LAC), while examining MA’s role in newly legal abortion services. | Women seeking abortion services in LAC | Descriptive analysis | The cost of one misoprostol pill, approximately US$0.75–$2.81 in the United States, can reach US$35 or even higher in LAC. In Brazil, misoprostol is limited to hospital use only and cannot be obtained in pharmacies, obstructing access to nearly 50% of South American women. |
| [64] [United States] | Use a trauma-informed lens to explore abortion-related hardships in a previously understudied group: patients in the United States who received ﬁnancial pledges from the National Network of Abortion Funds’ (NNAF) Tiller Memorial Fund, to pay for an unaffordable abortion. | Patients in the US who received financial pledges from NNAF to pay for an abortion (n=3,999). | Cross-sectional descriptive analysis | Patients from states that do not use state Medicaid funds to exceed coverage of abortions (outside of instances of rape or incest) had a higher average reporting of hardships related to abortion and abortion care. This ﬁnding was also true for patients from states that restrict the private insurance coverage of abortion, with patients from these restrictive states experiencing a higher average number of hardship experiences. |
| [65] [United States] | The aims of this paper are to discuss the results of a secondary data analysis of NNAF’s Tiller Memorial Fund cases that represent patients who received funding pledges to assist them with paying for an abortion. | Patients in the US who received financial pledges from NNAF to pay for an abortion (n=3,999). | Exploratory, descriptive, secondary analysis | The average cost of the abortion that patients were seeking to fund was $2,247.64. The average abortion costs were highest for adolescents (11-13), costing $4,015.64 and costing $2,695.43 (14–15) and $2,696.37 (16–17). Married women’s average costs were the highest at $2,605.99. Asian women reported the costliest procedures $3,613.44 resulting from procedures occurring at later stages of pregnancy relative to patients in other race categories. The average amount of funds the patients had to contribute was $535.02. The costs of abortions were highest for very young adolescents, suggesting that women in this group have significant difficulty gathering funds to pay for abortion procedures. Younger patients may not be fully employed, may not be comfortable asking parents or support persons for funding assistance, and they may also experience unique burdens, such as co-occurring psychosocial problems, which may increase delays in knowledge of the pregnancy and impact timing of abortions. The increased need for funding for second trimester procedures suggests that it may be becoming more difficult for women to gather the funding needed early enough to obtain the procedure in the first trimester. |
| [66] [United States] | The study aims were to assess the origination of funding requests by geographic region, federal Medicaid requirement, and private insurance restrictions, and to analyze the distances travelled to receive services. | Patients in the US who received financial pledges from NNAF to pay for an abortion (n=3,999). | Secondary descriptive analyses | On average, an expected distance of 228.17 km, SD = 446.90 (141.78 miles, SD = 277.69) was traveled to access an abortion. Those with pledges in states that restrict private insurance companies from covering abortions travelled greater expectant distances compared to those in states that do not. Those in states that did not exceed federal requirements for Medicaid travelled lesser expectant distances than those in states that exceeded. Of the 3,156, a total of 986 persons travelled out of state for an abortion, and persons who travelled out of state, on average, travelled nearly ten times the distance relative to persons who did not travel out of state for an abortion. Trimester was the most significant predictor of traveling out of state, with persons in their second trimester having a 3.40 times greater likelihood of traveling out of state for an abortion than first trimester pregnancies, and callers from states that did not exceed the federal Medicaid requirements a 2.62 times greater likelihood of travelling out of state relative to those from states that exceed the federal requirement. |
| [67] [United States, Republic of Ireland, Northern Ireland, Isle of Man] | To examine the experiences of abortion fund patients in the United States (USA) and Republic of Ireland (RI), Northern Ireland (NI) and Isle of Man (IM) to compare abortion fund patient experiences across these developed nations for the first time. | Select abortion fund patients within each country (n=6340 cases; 3995 from the USA and 2345 from the RI, NI and IM). | Cross-sectional descriptive analysis | National Network of Abortion Funds (NNAF) patients were contacting the fund approximately eight weeks further along in their pregnancies relative to Abortion Support Network (ASN) patients, which resulted in significant impact on the cost of abortion with US procedures costing over three times the amount. In a linear regression model where procedural costs were regressed on a binary variable, weeks pregnant explained 44% of the variation in procedural costs, while the datasets (representing organisation or country) explained 5% of the variation. While NNAF patients had a greater amount of funds to contribute to procedural costs, patients required a greater proportion of funding to have an abortion because the overall cost was three times greater. Conversely, while ASN patients had lesser funds to contribute, lower abortion costs allowed ASN patients to contribute to over half of the total costs. Results from the current study suggest that patient characteristics vary somewhat across countries, with characteristics of patients in the USA reflecting dire circumstances, including fewer resources and higher costs to cope with when compared to those in the RI, NI and IM. This is contrary to our hypothesis that circumstances from the ASN patients would reflect greater difficulties, given that abortion is banned with fewer exceptions there. |
| [68] [Ghana] | Explore the pre and post experiences of young people (aged 12 to 24) who had their abortion three months prior to the study. | Young people (12-14) who received abortion services at the Planned Parenthood Association of Ghana Cape Coast clinic (n=21). | Qualitative: in-depth interviews | The respondents were aware of both public and private health facilities where abortion services are provided. Respondents chose the PPAG clinic because it is youth-friendly, the cost of abortion is comparatively cheaper, the location of the facility is convenient in that it does not attract many potential ‘gatekeepers’ and gossips, and the attitude of staff is youth-friendly, compassionate, and sympathetic. Comparing the cost at the PPAG Clinic to other facilities elsewhere, they indicated that the former was economical. The cost of aborting a pregnancy in its first trimester ranged between $30-$40 accordingly and approximately $175 for second trimester abortions. In most cases, the male partners or both partners contributed to pay for the abortion services, unless the partner was not in favor of the abortion. The Coordinator of the facility indicated that the facility usually provides free services to young people who do not have money to pay for the services received. |
| [69] [United States] | The analysis seeks to measure whether young women really are less careful in using contraception if abortions are less costly, both in the context of ﬁnancial and opportunity costs, and explore the impact of direct and indirect abortion restrictions, | Data on women under the age of 25 seeking abortions. | Regression analysis | The results of this analysis indicate that legal restrictions on minors’ access to abortion have increased pill use among young women. This is especially true among the youngest women and in the earlier years of this analysis. Restrictions on minors’ access to abortion may have an impact that diminishes over time, thus the restrictions may be most applicable to the youngest women and fades as they age. In the context of abortion provider availability, this restriction is shown to matter most for the youngest women in our sample, with a diminishing effect over time and as women age into adulthood. Women have fewer ﬁnancial resources at younger ages, which may pre-vent them from traveling across state or county lines in order to ﬁnd a provider. |
| [70] [United States] | Analysis of state abortion legislation and proxying how the cost of obtaining an abortion varies across states, then assessing the implications of legislative changes on women’s contraceptive choices. | Data from the National Survey of Family Growth (NSFG) on women who are actively making an observable contraceptive choice (n = 7,070). | Regression analysis | Women making US$20,000 to US$59,999 a year are unaffected by both abortion restriction measures, meaning that their contraceptive behavior does not change as abortions become harder to obtain. This is a concern because these women may not be able to afford the additional travel costs associated with seeking an abortion, potentially resulting in an increase in unwanted births or illegal abortions. The poorest women (income < US$20,000/year) are affected in almost the same way as the total population of women. In addition, they are relatively less likely to use barrier and hormonal methods relative to no contraception. This means as abortion restrictions grow, there are some cases where they move toward less reliable forms of birth control. Finally, the richest women are rarely affected significantly. |
| [71] [United States] | Examine the steps in the process of obtaining abortions and women’s reported delays in order to help understand difficulties in accessing abortion services. | Women seeking abortion services (n=1,247). Location and facility details presented elsewhere [72]. | Mixed methods | Women who are financially disadvantaged also have difficulty obtaining early abortions. Lower-income women typically take more time to confirm a suspected pregnancy, which could relate to the cost of a home pregnancy test and the difficulty in getting a test from a clinic or a doctor. They also typically take several more days between deciding to have an abortion and actually doing so than their higher-income counterparts. Poor women (67%) were also more likely to say that they would have preferred to have had the abortion earlier than women above 200% of poverty (50%). The need to take time to make arrangements is the most common reason for delay for the sample as a whole, and low-income women are more likely to have this problem. Similarly, women who had second- trimester abortions were more likely to have concerns about cost or about raising money. Half of second- trimester patients reported that it took them a long time to decide, while only 35% of first-trimester patients said so; this finding was of borderline statistical significance (p=.06). However, second-trimester patients were more likely to cite worries about cost as a reason for delay in deciding. |
| [73] [Spain] | The objective of this study was to describe the determinants of the voluntary pregnancy interruption (IVE) delay until the second trimester of pregnancy in the city of Barcelona, between 2004 and 2005. | Women who reside in the city of Barcelona who obtained abortions for physical or mental health issues between 2004 and 2005 (n=9,175). | Cross-sectional study | The time of gestation and the moment in which the IVE occurs is important because it determines the abortion method that can be used, the risk of complications and mortality, and economic cost of the intervention. After adjusting the rest of the independent variables it is observed that women with primary education or lower have a prevalence ratio of 1.8 to delay the IVE until the second trimester (confidence interval of 95% [IC95%]: 1.4-2.2) compared with women with university education. |
| [74] [United States] | To examine and report on factors associated with abortion delay among a cross-section of patients at the San Francisco General Hospital Women's Options Center. | Women seeking elective abortion services at General Hospital Women’s Options Center (n=398). | Secondary data analysis of a cross-sectional study | “difficulty with getting MediCal to pay for the abortion” was significantly associated with delay during the second step of the abortion process. Several factors may contribute to difficulty with getting MediCal to pay for the abortion including women's lack of knowledge about available coverage, difficulty negotiating the MediCal application process or difficulty locating an abortion provider that accepts the MediCal payment. In the original analysis, results indicated that nearly a third of second-trimester women were still in the first trimester when they first called an abortion provider. This delay was associated with the following: having a prior second-trimester abortion, an initial referral to another clinic, an unsupportive partner, and difficulty financing an abortion. |
| [75] [United States] | To analyze data on women who sought and received an abortion at or after 20 weeks’ gestation for reasons other than fetal anomaly or life endangerment | People who were seeking abortions after 20 weeks gestation across 16 sites (n=272) and people who presented for first trimester abortions (n=169) | Mixed methods – qualitative data from interviews and quantitative data for logistic regression | The average cost of a first-trimester abortion was 519 USD and later abortions 2,014 USD.  “[the] financial part [slowed me down], because I could get there, but paying for it was the biggest problem.”  21-year-old Black woman in California – 22 weeks gestation  “I couldn’t afford it. They told me it was going to be $650, [but] by the time I was able to raise the $650, they had to do a different procedure, and so the price went up. The price jumped to $1,850...and they don’t take insurance.”  28-year-old Asian woman – 21 weeks gestation  Individual financial challenges also included costs associated with travel. People seeking first-trimester abortions spent an average of 18 USD in travel with a range of 0-400 USD. Women seeking later abortions spent an average 100 USD in travel with a range of 0-2,200 USD.  The following are case studies:  "Angel had difficulty finding a clinic where she could obtain an abortion. After visiting one facility that could not help her, she found another, three hours away. As late as she was in her pregnancy, the cost of the procedure was daunting: $2,700. But as Angel said, “I was determined.” She paid $300 herself, borrowed $400 from her mother and received aid from three funds that help low-income women pay for their abortions. She had her abortion at 24 weeks."  "Deciding to have an abortion was easy for Rose. Finding a clinic where she could obtain an abortion, however, was much more difficult. Rose called four clinics and visited another one before finding a clinic four and a half hours from her home that could perform her abortion at 20 weeks, for which she paid $1,750."  "Lesley left her husband. After counseling from a private therapist and with the support of two friends, Lesley got an abortion at 20 weeks’ gestation. The procedure cost her $1,700.” |
| [76] [Canada] | To document women's experiences obtaining abortion care in New Brunswick (NB) before and after the Regulation 84-20 amendment; identify the economic and personal costs associated with obtaining abortion care; and examine the ways in which geography, age and language-minority status condition access to care. | New Brunswick residents who received abortion services (n=36). | Qualitative: semi-structured interviews | Women obtaining hospital abortions in New Brunswick are required to undergo multiple visits, a process that costs women time and money. Although the abortion services are “free” at the hospital, some patients had to pay for travel and overnight hotel costs in order to attend multiple appointments in cities away from home. Some women also mentioned childcare costs and lost wages. For women who obtained clinic-based abortion care in-province, the costs were even greater, ranging from CAD400 (US$300) to CAD1600 (US$1225), excluding travel costs. Although Francophone women were unable to obtain abortion care in their preferred language, all of the Francophone participants described the overall inaccessibility of abortion care in New Brunswick as a more pressing issue and were more concerned about travel, cost and wait times than receiving French-language care. A small number of participants traveled outside of New Brunswick to obtain their abortion care. In addition to having to pay for both the costs of the procedure and the costs associated with travel, there seemed to be a lack of clarity about how the cost of the procedure was calculated, with prices up to $2000 (US$1535). |
| [77] [Zambia] | To examine men’s involvement in women’s abortion seeking in Lusaka, Zambia. | Women [n=112] who had undergone induced abortion at the hospital or had received post-abortion care there following induced abortion. | Cross-sectional descriptive | Some men were instrumental in securing care following an unsafe incomplete abortion, including payment for travel and the costs of care. Very few of the men helping in such cases were told the purpose of the care they were supporting or that the woman had attempted abortion unsafely. However, it is unclear how much these men knew of what was happening or if they preferred not to know. When directly involved in women’s abortion trajectories, men were most commonly providers of financial assistance for care seeking. |
| [78] [United States] | To assess the association of clinician referral with decision-to-abortion time. | English-speaking women aged 19 years and older presenting for an abortion for all indications at the three abortion clinics in Nebraska [n=263] | Cross-sectional survey | Reasons for delay among Nebraska women who would have preferred to have their abortion earlier: worry about cost (9%); time to raise money to have the abortion (28%); difficulty taking time off work (13%); difficulty arranging for childcare (4%). |
| [79] [Mexico] | To examine how a legislative discrepancy (legalized access in the city center vs. restricted access in the remainder of the muncipalities in the metropolitan area) affects access to public-sector abortion across the Mexico City Metropolitan Area. | Data from Mexico City’s public abortion program and census for 75 municipalities. | Case-control | Some of the barriers posed by local illegality in the context of proximate legal services include increased actual or perceived cost, a decreased level of knowledge about abortion services and increased stigma. The legal abortion program (ILE) framework indicates that only women in the city center are eligible to receive care for free (though a sliding scale for payments is often used for a majority of women seeking care from out of state). Regardless, perceived cost may inﬂuence decision making for women considering travel into the city center. The legalization of abortion in only part of the Mexico City Metropolitan Area has created a situation of reduced access to public-sector abortion services for the approximately 55% of the population of women of reproductive age living outside of the city center. Socio-economic status is also a key determinant of abortion access, especially in determining who is able to cross a border to access care. Even within the ILE program, adolescents and women with lower levels of education are more likely to present for care past the legal limit. |
| [80] [Vietnam] | To understand the determinants of delaying obtaining abortion until the second trimester. | Clients presenting for an abortion at 13–24 weeks of gestation in 5 health facilities in 3 provinces in Vietnam (n=60); abortion service providers (n=6) | Qualitative: semi-structured interviews | Despite sliding scale fees, about one-third of respondents recounted difﬁculties paying for the abortion. For example, a farmer related that the second-trimester abortion fee was equivalent to their annual savings. Some women described borrowing money to pay for the procedure or needing time to acquire enough money for the procedure. |
| [81] [India] | To explore adolescent women's access to abortion services, decision-making on abortion, determinants of provider choice and extent of morbidity experienced. | women who had undergone an induced abortion in three districts in Western Maharashtra in an 18-month reference period (1996–1998) [n=1717] | Mixed methods [interviewer-administered questionnaire, a qualitative in-depth time-line of sequence of events] | Cost considerations were the most commonly determinant of provider choice mentioned by adolescents (29.1%). Older women mentioned cost considerations significantly less frequently than the adolescent women (12.1%). The cost of the abortion depended on gestational age at which it was sought and the type of provider, but did not vary by age of the woman. The median costs that women incurred for a first trimester abortion in the private sector were Rs. 490.8 (US$ 10.20: Rs. 65–2625 / US$ 1.40–54.70). Public sector abortions were cheaper but not free of cost; the median costs for a first trimester were Rs. 177.2 (US$3.70: nil to Rs. 550 /US$11.50). Second trimester abortion costs in the private sector averaged Rs. 1661.4 (US$34.60) and in the public sector Rs. 560.8 (US$11.70). Indian law requires a guardian’s consent for young women below 18 years of age but some girls reported that private practitioners were willing to forego this requirement for a fee, between three to five times the normal rate. |
| [82] [India] | To understand how mifepristone and misprostol how they are being used, who is using them, how women access them or how providers, chemists, women and their partners perceive medical abortion. | A survey of 209 chemists, in the Indian states of Bihar and Jharkhand. | Cross-sectional descriptive survey | The belief that mifepristone was better was often due to its higher price, with cost being a proxy for quality. 8/9 chemists reported deciding on which drug to prescribe based on their estimate of the customer’s ability to pay. The bottom line for most chemists was to ensure that no one went away from the shop without buying a medicine. 6/9 chemists said that when the prescription is for mifepristone and it is not in stock, they have no option but to turn the person away, as there are no other abortifacient drugs in that price range that can be substituted. While the price of mifepristone in India is less than in many other countries, a single tablet still costs 8–10 times more than any of the other preparations and even in absolute terms can prove a considerable barrier in these two states, both of which have a per capita income considerably lower than the national average. The fact that higher than necessary doses of mifepristone continue to be prescribed and sold increases costs and further reduces access and therefore demand. |
| [83] [United States] | To evaluate the additional burdens experienced by Texas abortion patients whose nearest in-state clinic was one of more than half of facilities providing abortion that had closed after the introduction of House Bill 2 in 2013. | Texas-resident women seeking abortion services from ten facilities in Austin, Dallas, Fort Worth, Houston, and San Antonio. [n=398] | Survey | Out of pocket expenses >US$100 for 19.7% women whose nearest clinic in 2013 was open in 2014 vs. 31.9% of women whose nearest clinic in 2013 was closed in 2014 (p=0.04). |
| [84] [United Kingdom] | To better understand the experiences of non-resident women who travel to the United Kingdom (UK) seeking abortion services. | Non-UK residents seeking abortions at three British Pregnancy Advisory Service (BPAS) clinics (n=58). | Cross-sectional survey | Nearly all women in this study (97%) came to England for the express purpose of seeking an abortion. 75% travelled with a companion. The large majority of women travelled by airplane (95%) and stayed overnight (88%). Women paid an average of £631 (range: £20–£3000) for travel expenses, and an average of £210 (range: £0–£1000) for accommodation. More than half of women in this study found it ‘difficult or very difficult’ to cover the cost of travel. |
| [85] [South Africa] | To investigate the reasons for attempting self-induction, methods used, complications, and sources of information about informal sector abortion, and test a recruitment method. | Women [n=41] who sought informal sector abortion services in Cape Town, South Africa using respondent driven sampling (RDS). | Cross-sectional descriptive survey | Cost of informal sector abortion (Rand) (n=41) Mean=163R; SD, range: 146.8 (0-450); distribution: <50R = 12; 50-100R = 8; 100-250R = 7; 300+R = 14; missing = 1. Two women believed the cost would be lower outside the formal health system—despite the fact that public sector facilities are required to offer services for free. |
| [86] [Poland] | To present evidence from the Polish Tribunal on Abortion Rights | Testimonials of women who had managed to obtain an abortion, legally or illegally, and those who had not [n=7] | Qualitative | Illegal abortions cost between 2000 and 4000 PLN (US$500–1000). The average monthly Polish salary is 2000 PLN. The negative impact of the Act is therefore disproportionately greater on poorer women, and on those who do not live in large towns where services are more easily found. It takes women time to find the money and identify a willing provider. The fees for clandestine abortions were considered by the Tribunal judges to constitute a barrier in Poland. |
| [87] [United States] | The purpose of this study was to evaluate patients’ and providers’ experiences with telemedicine provision of medical abortion. | Women receiving telemedicine (n=20) or in-person (n=5) medical abortion services from Planned Parenthood of Heartland clinics in Iowa. | Qualitative: in-depth interviews | One dominant factor that drove women to opt for the telemedicine visit was closer proximity, along with the associated considerations of reducing the time they had to take off from work or school, limiting costs associated with travel, and avoiding having to explain the reason for traveling to a more distant location, among others. No women in this study cited telemedicine or the greater access to abortion services that it facilitated as a factor in their decision about whether to have the abortion. Instead, women reported reasons such as ﬁnancial insecurity, it being a bad time in life for a child, wanting to ﬁnish school, being too young, having completed their family, and not having a stable partner as their principal considerations. |
| [88] [South Africa] | To examine the acceptability of medical abortion among young people in Durban, South Africa.  To investigate the potential demand for and applicability of the method among women in South Africa | Sexually active women at the University of Durban and under 30 years of age (n=20) | Qualitative in-depth interviews | Private consultation fees are a major financial barrier – with reports of people needing ZAR 1000 to access these. The study reports that paying an approximate ZAR 900 for medical abortion at private clinics is unaffordable. |
| [89] [Global] | Review of protocols and existing evidence on follow-up care for abortion. | Global literature on follow-up visits for abortion care. | Literature review | Costs associated with this visit can be great. These include travel expenses, lost wages, child-care expenses, privacy and emotional burdens for women, and scheduling disruptions and the related opportunity costs caused by “no-shows” for the provider. With 87% of American counties lacking a single provider, approximately 25% of American women travel 50 miles or more for their abortions. Time spent traveling to and from the clinic, as well as in the waiting and examination rooms, may be taken away from work or school and may reduce earned income. These lost wages also may apply to the woman’s partner or another person who accompanies her to the visit. Women who attend a follow-up visit and have children must arrange child care on 2 separate occasions. |
| [90] [United States] | To assess the change in abortion services after the first three provisions [of a restrictive law HB2] went into effect. | Licensed Texas abortion facilities (n=22) | Observational study | The findings suggest that most—but not all--women desiring an abortion overcame the barriers of distance and additional cost to obtain the service they needed, following the implementation of HB2. |
| [91] [South Africa] | Article examines the policies that have regulated accessibility of abortion and assesses their impact on reproductive health. | South African policies. | Review of policies and related evidence | Prior to 1975, financially secure upper- and middle-class white women could fly to England to terminate an unwanted pregnancy if they could not procure adequate services privately in South Africa. In contrast, the relatively low-paying and insecure jobs available to black and colored women limited their ability to seek termination of an unwanted pregnancy. Besides the difficulty of financing a safe abortion, finding a trained doctor willing to perform an abortion was more difficult for women of color. |
| [92] [United Kingdom] | Estimate and compare the relative costs to the NHS of providing legal termination of pregnancy using medical abortion or vacuum aspiration; also to estimate and compare the relative financial costs incurred by women undergoing these procedures. | Women receiving legal abortion services at a Scottish teaching hospital (n=363). | Cost analysis | Medical abortion used 18 percent less of women's resources (213 pounds vs 259) when compared to vacuum aspiration. |
| [93] [United States] | To provide information on the percentage of women who travel long distances to obtain abortion services, the availability of abortion providers for second trimester services, the need to make more than one trip to the abortion facility and the amount abortion providers charge for services. In addition, it presents a measure of antiabortion harassment. | Abortion providers in the United States (n= 1,525) | Cohort | 8% of women having abortions in nonhospital facilities in 1992 traveled more than 100 miles for abortion services, and an additional 16% traveled 50–100 miles. The larger the facility, the higher the proportion of patients who travel long distances for services. If mandatory waiting periods are adopted by more states, travel to states without such restrictions will probably increase. Other women may choose a distant provider to take advantage of lower fees or to obtain services such as general anesthesia that may not be available from small local providers. Because most women go to the larger clinics with lower fees, the average patient paid about $296 for the abortion itself, not including other expenses such as travel, time missed from work and any additional medical services needed by a particular woman. The average amount paid in 1993 was 18% higher than the average amount paid in 1989. Women who live in large urban areas and have the necessary ﬁnancial resources can usually obtain abortion services in a single visit without a long wait for an appointment. Many women who have unintended pregnancies, however, have a low income and lack health insurance that covers abortion services. |
| [94] [United States] | This article documents the current status of abortion service accessibility in the United States, on the basis of data collected in a survey of all known abortion providers in the United States conducted in 2001–2002 by The Alan Guttmacher Institute (AGI) | Providers documented in the AGI Abortion Provider Survey (n=1,819). | Quantitative; survey | Respondents estimated that 8% of women having abortions in nonhospital facilities travel more than 100 miles to obtain this service, and that 16% travel 50–100 miles. In 2001, four states had legislation requiring most or all clients to receive specified in-person counseling at least 24 hours before the procedure is performed. Such requirements usually necessitate two trips to the abortion provider. On average, surveyed facilities charge $468 for a surgical abortion at 10 weeks LMP (last menstrual period). The lowest average charge ($364) is reported by specialized abortion clinics, and the highest average charge ($632) is reported by physicians’ offices. At 16 weeks, the mean and median charges ($774 and $650, respectively) increase. At 20 weeks, the mean and median charges increase to $1,179 and $1,042, respectively. In the second trimester, charges vary relatively little by type of provider, but the range remains wide, with some providers charging 2–5 times the average. In general, the mean amount paid by clients ($372) is lower than the mean amount charged by the typical provider, since larger providers (especially abortion clinics) tend to charge lower fees. However, clients obtaining an abortion at a doctor’s office pay substantially more ($471). The average self-paying client’s payment for an abortion at 10 weeks LMP has increased steadily over time—from $200 in 1983 to $319 in 1997, and to $372 in 2001. When cost of living inflation is taken into account, the amount changed little between 1983 and 1997, but increased by 9% ($30) from 1997 to 2001. When compared with the amounts paid for other medical care, the amount paid for abortion services fell from 1983 to 1997, and then increased by 5% ($17) between 1997 and 2001. |
| [95] [Nigeria] | To better understand morbidity resulting from unsafe abortion and to better assess its current magnitude and characteristics. | Women seeking post-abortion care (PAC) or abortion services and their provider in 33 hospitals (n=2093). | Qualitative: structured questionnaires | The greatest expense, averaging nearly 11,000 naira per patient, was for women treated for serious complications of abortion attempts outside the hospital. This total included about 7,900 naira for the hospital, which usually included physician care, 1,200 naira for supplies and 1,900 naira for medications. In addition, before going to the hospital, these women had paid an average of about 2,900 naira for the abortion attempts that caused the complications. In all, pregnancy termination and treatment cost about 13,900 naira for this group. Women needing treatment of spontaneous abortion paid less than half this amount (5,100 naira). Least expensive was abortion provided in the hospital for women who had made no prior attempts and those whose prior attempts had not resulted in serious complications (3,800 naira). Hospital care associated with abortion in the second trimester was much more costly than care associated with earlier abortions (with the exception of women who had serious complications from previous abortions). For women with complications of abortion attempts, the costs in private hospitals (5,000 naira) were lower than in other types of hospitals (10,300–16,200 naira): it is likely that public and mission hospitals were more costly because they receive patients with more severe complications and provide more comprehensive care. |
| [96] [Myanmar] | Describes the process undertaken by the Department of Health (DOH) in Myanmar to address the issue of abortion complications, by integrating post-abortion care and contraceptive service delivery into existing health care services | Health-providers [n=285] and post-abortion women [n=170]. | Cross-sectional descriptive survey | The cost of treatment was not a big factor in the decision to delay seeking care. Over 95% of women said that the cost of care was reasonable; two-thirds of those interviewed were charged under 6,000 kyat for inpatient care and medication, and on average they had spent 1,500 kyat prior to admission to hospital on transport and medication. An informal community support system exists for transport, and hospital staff keep a fund with donations for treatment of poor patients. |
| [97] [Australia] | To understand rural women’s experiences in obtaining a medical termination of pregnancy (MToP) through a rural primary healthcare service in Victoria, Australia. | Women aged 16 years and over who attended clinic between February 2016 and 2017 for an appointment related to MToP. [n=18] | Qualitative [semi-structured interviews] | Although surgical termination was available close by in the neighbouring regional city, women’s reasons for not using this service included cost, limited appointment availability, and avoiding protestors who picketed the facility. Women reported that the most important aspects of the MToP experience were the supportive services provided by the clinic staff, and the low cost of the MToP. Some women reported indirect costs associated with the process including travel time or missing time from work or education. |
| [98] [Hong Kong] | To describe the experience of teenage women in Hong Kong from deprived backgrounds who seek an abortion and the extent to which their access to legal services is constrained by age, income and class. | Young women [n=29] from deprived backgrounds in Hong Kong who had had an abortion, and key informants [n=4] | Qualitative cross-sectional descriptive | Among those who went to illegal abortion services, the cost of a legal abortion, the legal consequences for their sex partners of being identified, and the requirement of parental consent for public abortion services were key factors deterring them from using legal abortion services. The cost of abortion services was of great concern to all the respondents. Charges for an abortion ranged from a few hundred to more than tens of thousands of Hong Kong dollars. 5/29 respondents, either out of their own savings or with the financial support of family members, were able to afford the costs of private medical services. For the others, the first thing they had to do was to find the money needed for the abortion. Delaying abortion was not uncommon, caused by the time needed to find the money. |
| [99] [Burkina Faso] | to study both costs and consequences of induced and spontaneous abortions and complications | women [n=305] whose pregnancy ended with either an induced or a spontaneous abortion in Ouagadougou. | Cross-sectional descriptive survey | Women with induced abortion paid much more money to obtain abortion and treatment of the resulting complications compared with women with spontaneous abortion: US$89 (44 252 CFA) vs US$56 (27 668 CFA). Women who had had an induced abortion paid significantly more for the abortion procedure and treatment of its complications than women with spontaneous abortion: US$89 (44 252 CFA) vs US$56 (27 668 CFA), respectively (P < 0.001). They paid one and a half times the amount paid by women with spontaneous abortion for ending their pregnancy, and on immediate treatment linked to the abortion procedure before hospitalization: US$56 (28 065 CFA) vs US$37 (18 413 CFA), respectively. Women with induced abortion paid more than one and a half times the amount paid by women who had had a spontaneous abortion for treating complications resulting from their abortions: US$33 (16 187 CFA) vs US$19 (9255 CFA), respectively (P < 0.01). There was no evidence of a significant difference between induced and spontaneous abortion costs by type of facility. Women from low income households paid the highest amount of money for the abortion procedure and for subsequent treatment of its complications: US$105 (52 231 CFA). |
| [100] [Nigeria] | To document the characteristics and health conditions of the clientele of traditional birth attendants (TBAs) in southeastern Nigeria. | TBAs (n=13) and users of TBAs (n=147) in four rural communities. | Qualitative: interviews | 32 participants included ‘low charges’ as a reason they used TBA services. 3 clients were 20 and lower, 9 clients were in the 21-30 age category, 10 were between 31-40, and 10 were in the 41 and older age category. |
| [101] [Kenya] | How, in the context of Kenya's current abortion law as well as severe abortion stigma in the country, do ordinary women perceive and understand abortion safety? How do lay and public health discourses of abortion safety compare?" | A convenience sample of 50 women treated for complications of unsafe abortion at six purposively-selected public facilities in Kenya. | Qualitative cross-sectional descriptive | Pregnancy termination in hospital settings and by high-profile providers was considered very costly and often out of the reach of the poor. Women and young girls may not often have the resources to pay providers in these facilities to keep their abortions secret; women seek inexpensive but unsafe providers. Well-equipped facilities and providers were considered out of the financial reach of most abortion-seekers and thus expensive to use. |
| [102] [United States] | Our study sought to enumerate abortion barriers specifically  among a population of women seeking abortion care in the  latter half of the second trimester, when abortion care is most expensive and difficult  to obtain. | Data from Massachusetts referral program on women seeking second trimester abortion services (n=587). | Regression analysis of secondary data | Difficulty deciding whether to terminate the pregnancy, financial barriers and the woman not having realized she was pregnant until recently were the most common barriers. Though the sample was 89.7% insured at the time of referral to the program, some insured women experienced financial barriers to abortion, such as having private insurance that does not pay for abortion or having experienced a lapse in Medicaid coverage earlier during the pregnancy. |
| [103] [India] | To shed light on the experiences of unmarried young abortion-seekers aged 15–24, compare their experiences with those of their married counterparts, and explore the proximate factors leading to delays in them obtaining abortions into the second trimester. | A survey of abortion seekers and in-depth interviews with selected unmarried survey respondents [n=795 young women were surveyed: n= 549 unmarried, n=246 married]. In-depth interviews with n=26 randomly selected unmarried survey respondents. Sampled from facilities in two poorly developed neighbouring states in north India with weak health systems. | Cross sectional descriptive: survey and qualitative | The study was located in certified clinical settings of an NGO that charges a nominal fee (Rs 199 (US$4) for medical abortion, Rs 399 (US$9) for first trimester surgical abortion, Rs 799 (US$18) for second trimester surgical abortion). Its clinics are preferred to government clinics and other private health facilities by large numbers of poor women because they are reliable, confidential, of high quality and there are no hidden costs (e.g. drugs or tests). |
| [104] [United States] | To examine the breadth of barriers, beyond those related to individual state-level abortion restrictions, that women encounter and any associated consequences. | Patients seeking abortion services at six health facilities providing abortions in the states of Michigan and New Mexico [n=29] | Qualitative [in-depth interviews] | One respondent had been able to find an abortion provider that performed abortions past 20 weeks in a neighboring state, and had borrowed money to buy the plane ticket needed, but upon arriving, she had been turned away for being “too high risk.” |
| [105] [United States] | This paper applies a model of fertility control to estimate the responsiveness of teenage  abortion rates to variations in the local availability of abortion providers, | Data from 254 counties in Texas on teenagers seeking abortion services. | Regression analysis of secondary data | The results indicate that counties with higher travel costs have lower teenage abortion rates: the coefficient sizes imply that a $1.00 increase in travel cost would decrease abortions per woman by 0.86% and per pregnancy by 0.67%, other factors constant. To the degree that abortion services are available at nonlicensed facilities within a county, these coefficients may overestimate the true impact of travel cost on abortion demand–as licensed providers become less available, teenage women may substitute toward having abortions at nonlicensed facilities. |
| [106] [India] | To obtain information about where rural women seek care for abortion complications and about the quality of care they receive | Surveys of formal and informal organizations, institutions and key leaders in four selected villages in rural Uttar Pradesh. Community mapping exercises in four selected villages in rural Uttar Pradesh. 24 focus group discussions with informants from specific population subgroups, including married women and men, and single female and male young adults. 88 in-depth interviews with 53 key informants who were married women that the interviewers identified as being particularly knowledgeable about abortion and post-abortion care issues in their village. In-depth interviews with 38 post-abortion care providers | Mixed methods: Descriptive surveys, community mapping exercises, focus group discussions, and in-depth interviews | Women who experienced abortion complications generally first sought care from untrained or inadequately trained providers in their village. When their medical condition worsened, some women sought the services of providers who were more qualified but less affordable or less conveniently located. Treatment for abortion complications was widely available in the villages studied, but as this treatment was largely inappropriate, it tended to exacerbate rather than alleviate complications, cause delays in patients' seeking appropriate care and increase the total expense associated with treatment by necessitating multiple visits to providers. |
| [107] [United States] | To review laws that directly target abortion providers. | United States | Legal review | Factors causing women to delay abortions until the second trimester include cost and access barriers, late detection of pregnancy, and difficulty deciding whether to continue the pregnancy. Low-income women and women of colour are more likely than are other women to have second-trimester abortions. The cost of abortion is an important factor in access to care because abortions increase in price with weeks of pregnancy and are therefore more expensive later in the second trimester. When associated expenses, such as transportation, overnight lodging (because later second-trimester abortions require more than one day to perform), and child care are added, the price of abortion in the later second trimester rises dramatically. Three quarters of women receiving outpatient abortions to pay for the procedure with their own funds. Women with limited financial resources can find themselves in a vicious cycle: by the time they have secured the money for an abortion performed at one gestational limit, their pregnancy has advanced into the next. Lack of financial support for abortion results in delays that push the procedure into the second trimester. |
| [108] [United States] | To examine differences in abortion service delivery in Hostile, Middle-ground, and Supportive States. | All known abortion- providing facilities in the United States. | Cross-sectional descriptive. | After cost of living adjustments, a first-trimester surgical abortion was most expensive in middle-ground states ($470) and least expensive in supportive states ($402). On average, women paid $27 more for medication abortion at EMA-only facilities than at facilities that also offered surgical abortion. This pattern was consistent across the three policy climates, ranging from $9 more in hostile states to $35 more in supportive ones (after cost of living adjustments). The median charge for an abortion at 20 weeks gestation was $1,195 (data not shown). Clinics in hostile states charged the most, at $1,350 (adjusted for cost of living), and clinics in supportive states charged the least ($964); clinics in middle-ground states charged $1,158. |
| [109] [United States] | To understand how women pay for abortions, including ancillary expenses, in the United States. | Abortion care patients aged 15 or older at six abortion providers located in major cities in Arkansas, California, Georgia, Illinois, New Jersey, and Texas. [n=639] | Cross-sectional descriptive. | On average, women paid $382 for their abortion. 21% of abortion patients had $0 out-of-pocket costs. When women with $0 out-of-pocket costs are excluded, the average amount paid was $485, and up to $3,500 or more. Second-trimester abortions cost more: $854 on average ($652 when those paying $0 are included) compared with $397 ($319, respectively) for first- trimester patients. It was somewhat or very difficult for 41% of respondents to pay for the procedure (52% among women not using health insurance). The majority of those with health insurance did not or could not use it to pay for the procedure; 23%. 59% of women who were not using insurance and who were paying fully out of pocket indicated they obtained money from others, most commonly (60%) the man involved in the pregnancy. Regardless of whether the assistance came from an abortion fund, a male partner, or a family member, women’s most common characterization of having to obtain money from others was to feel grateful. Substantial minorities of women who obtained money from abortion funds and family members characterized the experience as “lifesaving,” although few women who obtained money from men indicated this response. A few women reported negative emotions such as “resentful,” “humiliating,” or “angry”. |
| [110] [United States] | To investigate the following questions: What are providers' experiences with Medicaid reimbursement for abortion in cases of rape, incest and life endangerment? What is the process for applying Medicaid reimbursement? What factors facilitate or hinder reimbursement? | Abortion providers in Florida, Idaho, Kansas, Kentucky, Mississippi, Pennsylvania, South Dakota, and Wyoming, where Medicaid funding is limited (n=25). | Qualitative: purposive interviews | The median costs were $450 for a medication abortion, $425 for a first trimester surgical abortion and $900 for a second-trimester abortion, but costs varied considerably. Respondents reported that the majority of patients pay for abortions out of pocket. |
| [111] [United States] | This study investigates the experiences of women seeking  abortion and their perceptions of a 24-hour waiting period between clinic visits. Using abortion patient survey data, we describe the financial costs women incurred when accessing abortion, assess anticipated emotional response to a change in the law, and identify women who may be particularly affected by a 24-hour waiting period. | Women seeking abortion services at a health clinic in Tucson, Arizona (n=379) | Qualitative: survey | The mean length of time travelled was 58 minutes, and 10% of the population travelled more than 2 hours to reach the clinic. The mean travel time was nearly 30 minutes greater among the participants below the federal poverty level (FPL). Women’s reports verified that a first trimester procedure at the clinic cost $450. More than one-half of participants reported having to miss work; Participants reported additional costs of transportation (33%), missed work (32%), staying overnight (4%), and childcare coverage (11%). Additional transportation and childcare costs were more likely to be forgoing payment for bills and food. Overall, 56% of women reported at least one reason that they were delayed from obtaining an earlier procedure, with the most common reasons being the costs of the procedure (39%), not knowing they were pregnant (30%), and taking time off work (18%). |
| [112] [sub-Saharan Africa] | To provide a basis for continued policy dialogue and reform to address the problem of death due to abortion complications among the East, Central and Southern Africa Health Community countries | Abortion complication patients and health providers at selected districts and tertiary care hospitals in three countries (Uganda, Malawi, Zambia) | Literature review    Reviews of logbook data and interviews | For the majority (53%) of patients interviewed, their spouse or partner was paying for the abortion treatment services received. Relatives (22%) and the patient herself (16%) were the other two major categories of payees. Overall, relatives paid for services at a higher rate (32%) among adolescent patients (£20 year olds) than among patients above 21 years (17%). Differences in payment practices were significant (p=0.015) by facility type. While 62% of patients in non-tertiary centers said a spouse or partner was paying for their treatment services, this was the case for only 37% of patients interviewed from tertiary centers. On the contrary, more clients (26%) from tertiary centers paid for services themselves than patients from non-tertiary facilities (11%). |
| [113] [Global] | Study examines evidence to suggest that the outcome of first-trimester abortions performed by suitably trained non-medical practitioners is comparable in terms of safety and efficacy to abortions performed by doctors. | Literature and data on national averages of costs to obtain an abortion. | Literature review | In Guatemala, where 37% of the population lives on US$2 a day or less, an abortion carried out by a midwife costs around US$38 in rural areas and US$128 in urban areas. In Uganda, where 85% of the population survives on US$1 day or less, the cost of an abortion obtained from a trained professional is around US$6–58, compared with US$6–18 for the services of an unskilled provider. Therefore, poor women who live close to trained mid-level health providers may still not access safe abortion care in many developing countries, as they are unable to afford their services. |
| [114] [Mexico, Colombia, Ecuador, Peru] | To collect information about women’s experiences using misoprostol or methotrexate plus misoprostol under clinical supervision in Mexico, Colombia, Ecuador and Peru. | Women [n=49] who had used either vaginal misoprostol alone up to a maximum of ten weeks of pregnancy LMP or intramuscular methotrexate followed by misoprostol up to eight weeks LMP. | Qualitative cross-sectional descriptive | In many settings, the cost of medical abortion with provider involvement, US$60–130, was less than for surgical abortion, which was important for many of them. In contrast, the cost of the misoprostol tablets, purchased at a pharmacy rather than through a provider, was US$15–78, and differed by place of purchase. |
| [115] [United States] | The purpose this study was to complete a cost effectiveness evaluation for women who received either PGE2 or misoprostol for a second trimester termination of pregnancy with either a living or dead fetus. | Medical records of women seeking abortion care services at Butterworth Hospital Grand Rapids, Michigan (n=78) | Cost comparison and analysis | Patients charged on average $548.21 for PGE2 and $298.86 for Misoprostol, for an average savings of $249.35. |
| [116] [South Africa] | To evaluate the likely costs of unintended pregnancy in South Africa using a deterministic modeling approach | Model estimates based on contraception prevalence and failure rates among women of reproductive age. | A decision analytic model to estimate costs of unintended pregnancies | The estimated cost per abortion was 3094 ZAR |
| [117] [Zambia] | To estimate the costs for women of seeking safe and unsafe abortion and to establish whether the burden of abortion care-seeking costs is equally distributed across the sample. | Women [n=112] receiving care for either safe abortion or post-abortion care at University Teaching Hospital [UTH], Lusaka | Cross-sectional descriptive survey | Women who sought a SA from UTH incurred the lowest costs (US$52.6) vs. PAC following an unsafe abortion (US$82.4). Of those women who sought PAC following an unsafe abortion, women who initiated a medical abortion unsafely incurred higher costs compared to women who used some other unsafe method (US$82.4 vs US$62.5). Women who sought PAC following a non-medical unsafe abortion incurred the highest relative miscellaneous costs. Irrespective of the type of abortion care sought, unofficial payments to health professionals represent a significant component of the costs that women have to pay. For women seeking a safe abortion at UTH, these payments represent 32% of the overall cost of abortion care-seeking. Whilst representing a lower proportion of costs (27%), the unofficial provider payments paid by women who initiated a medical abortion outside of the regulated sector were highest; this group of women also had substantially higher costs related to the purchase of medicines. For both safe abortion and PAC after an unsafe non-medical abortion, costs increase with wealth. |
| [118] [Canada] | This study compares the costs of four options for early medical and surgical abortion in Ontario. Costs are considered from the  perspectives of society, the health care system, and the patient. | Available data on abortion procedures in Ontario. | Cost analysis | The indifference price of mifepristone, at which the costs of mifepristone and methotrexate abortions would be identical, is $36.05 from the patient’s perspective and $59.89 from the societal perspective. In our model, the medical options for early abortion compare favourably with the surgical options in terms of total cost to society, the health care system, and the patient. |
| [119] [South Africa] | To assess women’s costs of accessing second-trimester labor induction and dilation and evacuation (D&E) services at four public hospitals in Western Cape Province, South Africa. | Women seeking second trimester labor induction and D&E services at four public hospitals (n=194). | Cost analysis | Although offered for free in the public sector, women incurred costs while accessing second-trimester abortion services. The median total cost for obtaining a second-trimester abortion, considering all participants, was $21.23 (R144.00). The participants made multiple visits to facilities while seeking an abortion, resulting in repeated transport costs. Induction procedures required more visits and more time and travel than D&E procedures. Few women lost income or paid for childcare during their visits. About one quarter of the women reported having paid for a pregnancy test. Despite public sector guidelines suggesting that the pregnancy test should be done at the facility, many clinics and hospitals in South Africa informally require that women present with a positive pregnancy test result. |
| [120] [South Africa] | To explore women's experiences accessing services and estimate costs incurred for first-trimester abortion at four public hospitals in KwaZulu-Natal Province, South Africa. | Women requesting first-trimester abortion services at one of four public hospitals (n=1,167). | Observational cohort design | 12.2% of all women who had a follow-up visit reported having missed work as a result of their abortion, and 5.4% indicated that they had lost income as a result of obtaining the procedure. The median travel time per visit (round trip) for all women was 50 min [IQR 30–80]. Almost all women (97.2%) reported having to pay for transportation, and 59.6% reported paying for a pregnancy test. The median recurring cost per visit for all women was US$3.23 [IQR 1.57–4.54] (ZAR 46.48 [IQR 22.59–65.33]). Combining recurring, per visit, costs and once-off costs for each woman, the median total cost for the procedure, considering the entire study population, was US$9.99 [IQR 6.46–14.85] (ZAR 143.76 [IQR 92.96–213.69]). Despite appearing low, the cost incurred by women in this study may have proved challenging as the majority of the study population was unemployed and dependent on family members for financial support. In 2010, the average monthly income for an employed, black individual in South Africa was ZAR 2167 [roughly US$151.69 (US 2010)] [23]. Thus, even for many working South Africans, US$9.99 (ZAR 143.76) for an abortion may not be insignificant. |
| [121] [Mozambique] | The purpose of the present study was to give a socio-economic profile of each of the three categories of women and to attempt to calculate the expenditure related to interventions necessary for the two abortion groups in terms of  hospitals cost and individual cost. | Women who had an illegally induced abortion (IA) (n=103), women who had a legally induced abortion (LA) (n=103), and women attending antenatal care (AC) (n=100). | Mixed methods | At an individual level the cost of a legal intervention is consequently unfavourable in comparison with the cost of an illegal one. The average individual cost of intervention was 46 contos for the LA group and 14 contos for the IA group. If salaried days lost (due to abortion-related complications) are also included, the mean total individual cost amounted to 49 contos for the LA group and to 36 contos for the IA group. |
| [122]  [Guadeloupe] | To describe the typical profile, and to assess the motivations of women who underwent illegal abortion with misoprostol in Guadeloupe (French West Indies). | Women who consulted at the Department of Gynecology and Obstetrics at the University Hospital of Pointe-à-Pitre / Abymes after failure or complication of an illegal abortion with misoprostol [n=52] | Descriptive prospective study | The costs of procuring medical abortion drugs illegally are cheaper (32 cents per tablet) than the official cost for treatment in a medical institution (50-60 Euros, although this can be exempted if a women qualifies for complimentary care). The official medical expenses are an important barrier to care for some women. Women who were migrants were more likely to self-use misoprostol because of a lack of access to social security. |
| [123] [Kenya] | To understand the methods married women aged 24–49 and young, unmarried women aged ≤ 20 used to induce abortion, the providers they utilized and the social, economic and cultural norms that influenced women’s access to safe abortion services in Bungoma and Trans Nzoia counties in western Kenya. | Focus groups [n=10] conducted in Trans Nzoia and Bungoma county with un/married and younger/older women in rural and urban settings. | Qualitative cross-sectional descriptive | The cost of an abortion ranges from KS 60 for quinine purchased at a pharmacy to 5,000 KSH (US$ 60) from a doctor, although costs were negotiable with individual doctors. Women in 6/10 groups considered the cost of abortion from public hospitals or private providers to be prohibitive, and used less expensive options, such as traditional drugs and herbs. Even where women knew about a potentially safe option for abortion, the cost was prohibitive and limited them to less expensive options. Most women in western Kenya earn less than 220 KSH (US$ 2.50) per day, so a procedure that costs 950–2,000 KSHs, is prohibitively expensive. Women and young women reported similar costs of abortion services in their communities and both sought less expensive options. |
| [124] [United States] | This paper addresses the gap in  the literature by empirically examining two important public and social policy questions: Do  restrictive state abortion laws increase the price charged by abortion providers? And, if so, does the increase in the price charged by abortion providers, as a result of complying with the restrictive state abortion laws, have a significant negative impact on abortion demand? | Data from the Guttmacher Institute. | Regression analysis of secondary data | The empirical results consistently show that abortion providers in states that fund Medicaid abortions have higher abortion prices most likely because they incur higher expenses from providing Medicaid abortions. The enforcement of two-visit laws and TRAP laws in a state is associated with an increase in the price charged by abortion providers presumably in order to cover the additional expenses of complying with these laws |
| [125] [Bangladesh] | To investigate the knowledge, attitudes and practices regarding mHealth of both MR [menstrual regulation] clients and formal and informal sexual and reproductive healthcare providers in urban and rural low-income settlements in Bangladesh. | MY clients (n=24) and health service providers (n=24) | Qualitative | Although MR is provided free of charge, or at a nominal cost, through the public sector and various non-profits organizations, many women face barriers in accessing safe, affordable MR and post-MR care. 5/24 MR clients stated that obtaining health-related advice over the phone saved them the price of a medical visit and enabled them to avoid paying the cost of transportation to a clinic. Formal and informal providers have to bear the cost of their mobile phone credits in order to communicate with their clients. |
| [126] [Zambia] | To compare the financial costs for women when they have an induced abortion at a facility, with costs for an induced abortion outside a facility, followed by care for abortion-related complications. | Household wealth data at one point in time (T1) and longitudinal qualitative data at two points in time (T1 and T2, three-four months later), in Lusaka and Kafue districts | Prospective quantitative and qualitative. | Younger patients were less likely to know the costs that were paid. Among women who knew what had been cumulatively spent in the process of obtaining an abortion, both on the procedure and on ancillary expenses, approximately one-third of the respondents had spent less than 300 ZMW (<USD 47); two-thirds had incurred expenses between 300 KWZ and 1000 ZMW (USD 47–155); and 2 patients incurred over 1000 ZMW in expenses (>USD 155). Women who had case for unsafe abortion (CUA) spent on average, 30% more than women who obtained a TOP. The average cost spent on a TOP for all services was 396 ZMW (USD 62) vs. CUA 520 ZMW (USD 81). About two-thirds of the costs had been incurred by T1, while for women who had experienced either a TOP or CUA, an additional one-third of the total costs was incurred between T1 and T2. Women who went to a public hospital for a TOP incurred expenses which, on average, were less than half (210 ZMW, USD 33) the expenses of women who went to a private clinic (463 ZMW, USD 74). CUA cost, on average, at the public hospital (542 ZMW, USD 84) than a private clinic (450 ZMW, USD 70). Women who sought either a TOP or CUA at a private clinic paid about the same (463–474 ZMW, USD 72–73). In some cases, other costs were opportunity costs which were incurred as the news of the abortion spread and stigma was enacted on the woman in her social or professional life. |
| [127] [United States] | To document the experience of buying abortion pills from online vendors that do not require a prescription and to evaluate the active ingredient content of the pills received. | Websites [n=18] that sold mifepristone and misoprostol to purchasers in the United States. | Quantitative cross-sectional descriptive | The price for the 18 mifepristone–misoprostol products ranged from $110 to $360 (median $204.50), including shipping and fees; the products without mifepristone cost less. |
| [128] [United States, UK, France, Sweden, India, China] | To review the costs of abortion services | Review | Review | In 1997, the cost of a first trimester non-hospital abortion under local anaesthesia ranged US$150 – 1535 (average US$316). Medical abortion using methotrexate regimens ranged US$100 – 1250 (average US$401). Average cost of a second trimester procedure (US$618), to over US$1100 at 20 weeks. The cost of medical abortion was often set higher than the cost of surgical abortion because of assumptions about the procedure and follow-up. Despite a price differential, many patients would choose to have a medical abortion; however, utilisation would drop if the price difference was > US$50. |
| [129] [Kenya] | To illustrate how the quality of PAC in healthcare facilities is impacted by law and government policy. | Patients [n=21] and providers [n=16] at 16 hospitals in three regions sampled purposively by regional area, level, and reported quality of care. | Qualitative cross-sectional | In smaller facilities patients were not referred for specialized surgical interventions due to the transfer costs. Patient referrals increase the out-of-pocket cost of treatment, particularly referral from public to private facilities. Lack of payment options for patients necessitated the use of brokers, who would assist patients to make payments at a fee. Patients were often required to make cash payments for procedures, restricting services to patients with a credit/debit card. The requirement that patients paid prior to each procedure restricted access to timely care. Inability to pay for services led to multiple referrals. In private facilities, providers reported that patients often could not afford services at all. In addition to direct treatment costs, even in public facilities other indirect costs such as toiletries and bathing accessories increased out-of-pocket expenses. |
| [130] [Kenya] | To evaluate adolescents' behavior regarding induced abortion. | Adolescent girls and boys ages 10-19 years in rural and urban Kenya. | Cross-sectional survey | The amount paid to procure abortion reported ranged from KS 100 to KS 30,000 (US$2 to 500). The cost of abortion services was mostly paid by boyfriends for adolescent girls, and by parents for boys. |
| [131] [Pakistan] | To study the cost of induced abortions (unsafe abortion) and treatment of the complications of such abortions. | Women admitted to four tertiary hospitals in Lahore for complications of unsafe abortion [n=100] | Cross-sectional survey | Average cost of abortion was Rs.1686 (US$ 28) (range Rs.500-7,000); average cost of treating complications was Rs. 4,197 (range Rs. 1600-45,000). |
| [132] [Australia] | To investigate the extent and cost of travel undertaken by women accessing Victorian termination of pregnancy services. | Women receiving privately funded pregnancy termination services [n=1,244] | Multi-centre, cross-sectional observational study. | Women face substantial and immediate costs beyond the service fee, as well as the difficulties associated with poor continuity of care and significant time away from home. |
| [133] [Nigeria] | Evaluation of an intervention to improve the quality of private sector provision of post-abortion care in northern Nigeria. | Private medical doctors [n=458] and nurses and midwives [n=839] | Cross-sectional survey | The cost of procedures ranged from zero cost to a maximum of Naira 10 000 (US $66.7) (mean, Naira 2488±1745, US $16.5±11.6). 570 procedures (22.3%) were carried out gratis. The procedures were most commonly paid for by husbands (43.7%), the women themselves (15.9%), parents (8.9%), other relatives (5.9%), and boyfriends (3.3%). |
| [134] [Nigeria] | To uncover the motivations and experiences of private medical practitioners of abortion services. | Private medical doctors [n=34] | Cross-sectional survey and in-depth interviews | Respondents charge an average 5, 000 naira for an 8-week pregnancy. |
| [135] [Burkina Faso] | To show how economic resources, social networks, social norms and education affect the risk of clandestine abortion. | Case studies [n=2] from health facilities in Ouagadougou. | Qualitative cross-sectional | A case bought Misoprostol for Fr5000, Fr7000 for ultrasound, Fr3000 for PAC and Fr2075 for a prescription. |
| [136] [Chile] | To document the experience of clandestine medical abortion use among university students in Chile | 30 students aged between 17-26 years who had had medical abortions and attended 10 universities in the Santiago Metropolitan Region | Qualitative in-depth interviews | Respondents reported the role of the seller in providing the right pills in the right quantity for an affordable price.  A respondent reported the following interaction with a seller:  “He said: ‘I have 2 types of pills. One that costs $70,000 for 4 misotrol, and one that costs $ 90,000 which includes 6 mifepristone, which I think is the best dose to use. It's up to you’.” |
| [137] [Kenya] | To explore the pathways, decision-making, experiences and preferences of women receiving safe abortion and post-abortion family planning (PAFP) at private clinics in western Kenya. | Women [n=22] who had received an abortion or post-abortion care service at one of nine clinics. | Qualitative cross-sectional | Cost of the service was a consideration, with some facilities having a reputation for being more affordable. For some women, cost was a low consideration relative to the need to get an abortion |
| [138] [Switzerland] | To compare the Swiss Criminal Code about TOP with its implementation by cantons and health professionals, including the cost of a TOP. | Women [n=281] before or after termination of pregnancy | Cross-sectional descriptive | TOP ranged CHF400-3500; average cost CHF1360. Successful medical TOP (average cost 1076 CHF), surgical TOP with general anaesthesia (average cost 1490 CHF); unsuccessful medical TOP, followed by a surgical TOP with general anaesthesia (2312 CHF) (significant differences p = 0.002; ANOVA p = 0.02). Costs vary depending on the size of the health care institution: private doctors' office (400-1270CHF, average: 620 CHF); average-sized, non-university hospital (592-2550 CHF, average: 1247 CHF); university hospital (693-3500 CHF, average: 1529 CHF). |
| [139] [Anguilla, Antigua, St Kitts, Sint Maarten] | To contribute to an improvement in abortion care in the region, we sought to identify practitioners who were qualified, informed and potentially influential | Interviews with physicians (n=26), of whom 16 provided abortion care | Qualitative in-depth interviews | On the costs of abortions, a physician and abortion provider reported:  ‘‘Women have come to me and said, ‘Doctor, I want to have a termination but I cannot afford the $1400 Eastern Caribbean [=US$560]). What can I do?’ And I’ve said, ‘This is what you can do, you can go to the pharmacist, they can give you these tablets, and if you have any problem you come back and see me.’’ |
| [140] [Thailand] | To gather evidence on abortion among women and their partners | Women (n=103) with a history of self-managed misoprostol use attending Department of Obstetrics and Gynecology, Maharaj Nakom Chiang Mai Hospital | Qualitative in-depth interviews | The mean cost of misoprostol reported by women was 663.16 Baht – calculated from self-reports by 88 of the respondents who knew the costs. |
| [141] [Cambodia] | To describe the loss of productive time and income related to abortion care and care-seeking. | 160 women seeking elective abortions or care for complications of abortion from a purposefully selected group of public and private clinics and hospitals. | Cross-sectional survey | The mean productive days lost increased as the number of visits to terminate the pregnancy increased. Average time spent seeking and receiving care for women who required only one visit was 2.3 days. Women who needed three medical or pharmacy visits seeking advice or medication used more than 1 month (33.5 days) of productive time. Women wage earners reported losing the least amount of time obtaining an abortion. Women in the informal sector reported the greatest amount of lost productive time. The mean cost of 2^nd^ trimester services was much higher than the mean cost for women with first trimester pregnancies. Women who first sought care at the office of a private physician reported almost no loss of earnings. Women who first approached private midwives also reported minimal lost earnings, followed closely by the women who went to government facilities. Lost wages that began with care-seeking from pharmacists were substantially higher than amounts lost seeking care from all other types of providers. |
| [142] [Nigeria] | To identify near miss events and the proportion due to unsafe abortion, and the characteristics of women with these events, delays in seeking care and the short-term socioeconomic and health impacts on women and their families. | women of childbearing age with maternal near-miss or at risk of maternal near-miss due to unsafe abortion | Cross-sectional survey | Cost of treating life threatening complications due to unsafe abortion was six times higher than the cost of all attempts to end the pregnancy. Women spent on average 12,586 Naira (US$8316) in all attempts to end the pregnancy; average cost of treating the severe unsafe abortion complications was 74,407 Naira (US$488). Boyfriends (six out of 11), and to a lesser extent family members, were most commonly responsible for paying the cost of the abortion procedure. About half of the women did not know who bore the cost of treatment. |
| [143] [India] | To examine women’s decision-making processes, the types of facility they attended and the extent of post-abortion complications they experienced | Women [n=97] who had had abortions within the previous six months | Cross-sectional descriptive qualitative | Fee for a first trimester abortion ranged Rs.800–1000 (US$20). The typical woman wage labourer in the district earned Rs.1000–1200 per month. The cost was much higher if the pregnancy was advanced or if the woman was unmarried. Unqualified and unsafe abortion providers were cheaper at Rs.200–300 (US$6) for first trimester abortion. Abortion costs of well-equipped and qualified providers was a serious challenge to family resources; the majority of women (and their husbands) were willing to pay the costs because of the lower risk of post-abortion complications. Unmarried women are particularly vulnerable to dangerous abortions from unqualified providers because of their concerns about cost and secrecy, the tendency to delay seeking services and the unwillingness of qualified providers to help them. The perception of poor quality of care in government PHCs and hospitals was a major reason why most women chose private practitioners if they could afford the costs. |
| [144] [United States] | To describe payment for abortion care before new restrictions among a sample of women receiving first and second trimester abortions. | English- and Spanish-speaking women aged 15 and older, with no known fetal anomalies or demise, presenting for abortion care at one of 30 facilities throughout the United States between January 2008 and December 2010 and meeting specific gestational age criteria. | Interviews and regression analysis | Median price of a first trimester abortion $490 ($225-$750, mean $497). No significant differences in prices of first trimester medication versus aspiration abortions. Median price between 14 and less than 20 weeks $750 ($490-$1,500, mean $860). Median price at or after 20 weeks $1,750 ($946-$6,008, mean $1,874). Median out-of-pocket costs when private insurance or Medicaid paid were $18 and $0. Median out-of-pocket cost for women for whom insurance or Medicaid did not pay was $575. For more than half, out-of-pocket costs were equivalent to more than one-third of monthly personal income; closer to two thirds among those receiving later abortions. More than half reported cost as a reason for delay in obtaining an abortion. |
| [145] [Burkina Faso] | To examine the choice of confidants with whom young women share their abortion secret, the probability of leaks and the extent of gossip that exists in a rural and urban social environment. | Key informants [n=13], male and female | Qualitative cross-sectional descriptive | The cost may range from a few thousand CFA francs for traditional potions or abortifacients of varying effectiveness, tens of thousands for an injection by a health worker, and up to 200,000 CFA francs for a curettage in hygienic conditions. The monthly wage for a maid or caretaker is 20,000–40,000 CFA francs; an abortion using a medically safe method (even in unhygienic conditions) is beyond the reach of the poorest population groups. In villages, where the main source of income is the sale of agricultural produce, individuals have little ready cash. Abortionists – even those using medically safe methods – charge no more than 10,000 CFA francs. In most cases, the young women will ask older relatives or the partner for money using a fabricated excuse. |
| [146] [Mexico] | To know whether factors [fear of staff attitudes, ignorance about the law, lack of information on where to access services, fear of breaches in confidentiality, burdensome requirements] were driving women in Mexico City to continue seeking private abortion services despite the availability of low-cost, safe, legal abortion services in the public sector. | Health providers working in lower middle-class neighbourhoods with small community health facilities [n=135 private sector physicians] | Descriptive | Fees charged ranged from a minimum of US$35 to a maximum of US$1,109and varied depending on the method and the clinic. D&C was most expensive ($35-$1,109), MVA ($35-$887) and medical abortion ($37-$443). The cost of a private abortion was high and the use of D&C, ultrasound and general anaesthesia, along with keeping women in the clinic overnight, contributed to this. |
| [147] [Cameroon] | To examine the histories of four young women who became  pregnant and had an abortion in the Anglophone region of the Cameroon Grassfields. | Young women [n=4] who became pregnant and had an abortion in the Anglophone region of the Cameroon Grassfields. | Cross-sectional descriptive qualitative | Costs are highly dependent on the provider, gestational age and type of abortion. |
| [148] [Canada] | A pilot study of a questionnaire aimed at tracking the abortion journeys of individual women to a Canadian abortion clinic | Women [n=1022] attended Toronto Morgentaler Clinic, a private non-profit abortion clinic | Pilot study to test a questionnaire | Approximately 90% of women spent less than 50 CAD on transportation to and from the clinic, 20% spent nothing.  The majority of women had no accommodation expenses, however, those that did predominantly paid more than 100 CAD.  Expenses varied significantly, including one respondent who spend 4445.05 CAD for travel and hotel accommodation. Expenses increased depending on child care needs, if people accompanied, loss of wages. |
| [149] [Canada] | To analyse the travel women undertake to access abortion services at freestanding clinics. | Women [n=1186] seeking abortion care at 17 freestanding abortion clinics. | Cross-sectional survey and mapping | Women's travel costs ranged from “nothing” (15.6%) to more than $100 (CDN) (5.4%). Some women (38%) incurred other expenses ranging from less than $10 (CDN) (9.5%) to more than $100 (CDN) (3.1%). Costs were often doubled as the majority of women reported travelling to the clinic with someone (73.1%). Although abortion is supposedly a funded, medically necessary procedure, 22.1% of women reported that they paid for their own abortions. Of these women, 19% paid for the abortion procedure and for travel to the clinic, likely supplementary expenses such as administrative fees, medication or follow-up contraception rather than for the abortion procedure itself. The 25.3% of women who reported paying more than $300 likely paid for the abortion procedure itself. Whether or not these women would later be reimbursed by their provincial government health care plans for the abortion procedure is unknown. |
| [150] [Australia] | To examine access and equity to induced abortion services in Australia, including factors associated with presenting beyond nine weeks gestation. | Survey of 2,326 women aged 16+ years attending for an abortion at 14 Dr Marie clinics. | Cross-sectional descriptive survey | Abortion costs are substantial, increase at later gestations, and are a financial strain for many women. Under 1% reported an abortion free of cost, and of this group, nearly half cited an external organisation as their source of financial assistance. 97.6% had Medicare coverage, however 5.0% did not use or were unsure about using it to subsidise costs. Among women who used Medicare, the reported median upfront cost of a medical abortion was $560 vs. $470 for a surgical abortion at ≤9 weeks, however the final out-of-pocket direct costs for the patient are the same. Without the Medicare rebate or PBS reimbursement for the cost of the medicine, the median costs for both medical and surgical abortions at ≤nine weeks roughly doubled. As women progressed beyond the first trimester, the cost of the surgical procedure rose at key gestational intervals, whilst the value of the Medicare rebate remained constant. For women who used the rebate, the median out-of-pocket costs for surgical procedures at 13-19 weeks and beyond 19 weeks were three ($1,500) and 16 times ($7,700) greater than the median out-of-pocket costs within the first trimester ($470). About 41% reported indirect costs related to travel and accommodation, GP referrals and medical tests, childcare and lost wages. The median cost for these additional expenses was $150. Women who had difficulty paying (AdjOR: 1.5, 95%CI 1.2-1.9) were more likely to present ≥9 weeks. |
| [151] [Global] | To review the scientific evidence on the consequences of unsafe abortion, highlight gaps in the evidence base, suggest areas where future research efforts are needed, and speculate on the future situation regarding consequences and evidence over the next 5–10 years. | Women who had unsafe abortions. | Review | Unsafe abortion has a number of significant consequences that are much less widely recognized: economic consequences, the immediate costs of providing medical care for abortion-related complications, the costs of medical care for longer-term health consequences, lost productivity to the country, the impact on families and the community, and the social consequences that affect women and families. |
| [152] [Kenya] | To analyze the socioeconomic and reproductive background characteristics of women with incomplete abortions and assess post-abortion health consequences and financial implications for the women and the hospital. | Women seeking help for incomplete abortions at Kenyatta National Hospital. | Cross-sectional survey | Cost of unsafe abortion procedures averaged 470 Kenyan shillings (range 0 to 3000). Total average cost of treating unsafe abortions was 348 KS compared to 87 KS who were treated for spontaneous incomplete abortions. |
| [153]) [Sweden] | To calculate the cost-effectiveness of early medical abortion performed by nurse-midwifes in comparison to physicians in a high resource setting where ultrasound dating is part of the protocol. | Women attending an outpatient clinic of a university hospital in Sweden | Modelling | Comparison of cost of a patient’s time for non-complicated care: cost standard (Euro14); cost intervention (Euro10). |
| [154] [Late Imperial China] | A historiography of abortion in Late Imperial China | 8 legal case studies in historical documents of abortions | Legal case studies | "Cost of abortion in eight legal cases, with place and year:  1) 3 taels of 70-percent cash (= 1.5 shi of husked rice); Jin’gui county, Jiangsu, 1773  2) 1,500-4,500 cash; Yongbei subprefecture, Yunnan, 1774  3) 1,000 cash; Yanghu county, Jiangsu, 1781  4) 4,100 cash; Cangwu county, Guangxi, 1788  5) 4.5 shi of unhusked rice (= 2.25 shi of husked rice); Lingling county,  Hunan, 1800  6) 2,000 cash; Gao’an county, Jiangxi, 1802  7) 5 taels of silver; Guzhou subprefecture, Guizhou, 1815  8) 5 taels of silver; Yuqing county, Guizhou, 1842" |
| [155] [Brazil] | To analyze the trajectory of women that performed a provoked abortion related to a clandestine procedure. | Women hospitalized for post-abortion care in a public hospital in Salvador [n=17] | Qualitative cross-sectional | Respondents reported borrowing money to abort, and spending relatively substantial amounts of money to procure unsafe abortions. |
| [156] [Uganda] | To measure the costs of unsafe abortion and post-abortion care to Ugandan women and their households. | Ugandan women [n=1,338] who had been admitted to one of 27 health facilities for treatment of complications of abortion were interviewed while at the facility. Two to three months later, follow-up interviews completed with n=517 women. | Prospective survey | Respondents who likely had had an unsafe abortion had paid an average of 59,600 shillings (US$23) in out-of-pocket expenses for the procedure, for the treatment of complications prior to arriving at the facility, or both. In addition, on average, women paid 41,800 shillings (US$16) in out-of-pocket costs for treatment of post-abortion complications. At follow-up, women reported having paid an additional 26,700 shillings (US$10) for medical expenses incurred between the first and second interviews. The average total out-of-pocket expenditure per woman was 128,000 shillings (US$49). The amount paid to obtain an unsafe abortion was higher among women who were younger than 20 (68,100 shillings), childless (77,000), unmarried (73,300), attending school (94,500), residing in urban areas (71,500) or in the wealthiest group (81,900 shillings) than among women in the respective reference groups. Expenses women incurred for post-abortion care were higher among women who had spent two or more nights in the health facility (58,900 shillings) than among women with shorter stays (28,900–29,500). Facility and provider characteristics were also strongly associated with the level of expenses incurred. Women who had received post-abortion care at private or nonprofit facilities incurred higher expenses than did women treated at public facilities (153,000 vs. 32,300 shillings), and women who were treated by doctors paid more than did women who received care from providers other than doctors or nursemidwives (46,900 vs. 30,200). Women who did not have any children incurred higher post-abortion care expenses than did women with three or more children (46,800 vs. 37,700). No subgroup differences were observed at the follow-up interviews for post-abortion care expenses. |
| [157] Côte d’Ivoire | To analyse how illegally induced abortion is understood in terms of social processes. | Women seeking care for abortion complications and key informants | Qualitative cross-sectional | D&C abortions could be obtained at hospitals and clinics for between US$ 30-70. |
| [158] [Nepal] | To assess and evaluate the safety, acceptability, and effectiveness of MVA services. | Women seeking abortion services at the largest national maternity hospital in Kathmandu [n=765] | Cross-sectional comparative | The basic fee per patient (excluding other related costs, if any) is Rs 645, representing approximately five days' wages for a female labourer in the Kathmandu Valley. In addition, ultrasound is used in about a quarter of MVA cases (Rs 300). |
| [159] [Thailand] | To document the experiences of women with unwanted pregnancies who accessed the SARP in order to inform programme improvement and expansion. [SARP = Safe Abortion Referral Programme] | Migrants of Burmese ethnicity to Thailand | Cross-sectional qualitative descriptive | Undocumented migrant women stated that without the SARP they would not have been able to access a safe abortion because of both the financial and legal implications of seeking and obtaining care. |
| [160] [United States] | To examine whether the 2011 Ohio law change from an evidence-based regimen to the FDA regimen (as approved in 2000) was associated with the need for additional intervention following medication abortion. Additionally, to examine the number of follow-up visits, continuing pregnancy rate, experience of side effects, proportion of medication abortions (versus other abortion procedures), and average patient charges for medication abortion. | Medication abortion patients from 1 year prior to the law’s implementation (January 2010–January 2011) to 3 years post implementation (February 2011– October 2014) at four abortion-providing health care facilities in Ohio. | Pre/post-observational study with no control group that was not exposed to the law | The average patient charge increased from US$426 in 2010 to US$551 in 2014, representing a 16% increase after adjusting for inflation in medical prices." [p.1] " "The average patient charge for medication abortion rose from US$426 to US$551 between 2010 and 2014, representing a 29% increase in nominal dollars and a 16% increase after adjusting for inflation in medical prices |
| [161] [United States] | To examine the association between distance traveled for an abortion and site of post-abortion care among low-income women. | Claims data from California’s Medicaid programme (2011-12) [n=39,747 abortions] | retrospective cohort study | Costs were consistently higher when subsequent care occurred at an Emergency Department rather than the abortion site (median cost $941 vs. $536, P<.001). |
| [162] [United States] | To assess medication abortion access among California’s public university students. | California’s public university students. | Modelling | The average out-of-pocket cost of medication abortion at the facilities closest to campus was $604. |
| [163] [United States] | To determine total patient costs for medication abortion | Women [n=212] seeking abortion services at a convenience sample of 5 health care practices. | Descriptive cross-sectional survey | The mean total cost for medication abortion was $351 ($0–1,140). Average charge $306 paid by women themselves for the procedure. Three quarters of total costs were direct medical costs and almost one quarter was time away from work and other activities. Although nearly three quarters of the women were insured, only 1% used insurance to cover their abortion—many (44%) did not know if their insurance covered abortion. |
| [164] [India] | To understand gynaecologists’ perspectives on medical abortion and chemists’ marketing strategies and drug distribution. | Gynaecologists and chemists | Qualitative interviews | Total cost of medical abortion estimated to be Rp1,200 (inclusive of 3 ultrasounds); mifepristone tablet (Rp325-930); Misoprostol table (Rp31-60). |
| [165] [Bolivia, Brazil, Ecuador, El Salvador, Mexico, Peru, Egypt, Ghana, Kenya, Malawi, Nigeria, South Africa, Tanzania, Uganda] | To estimate health care system costs of unsafe abortion in Africa and Latin America. |  |  | The average cost of post-abortion care per patient, based on all samples from 20 studies, was $86 (2006 US$); costs ranged from $2 to $390. The average cost per patient for treating low-severity complications was $72, while the average cost for samples of women with all types of complication was $111. Costs varied little by region, ranging from $83 in Africa to $94 in Latin America; the average for Sub-Saharan Africa was $89. Even though the cost per patient in U.S. dollars was highest in Latin America, after conversion to international dollars, the average costs in Sub-Saharan Africa and in Africa as a whole were substantially higher ($228 and $213 vs. $161). This demonstrates that in relation to patients’ purchasing power, abortion complications are considerably more expensive to treat in Africa than in Latin America. |
| [166] [Ethiopia] | To address the knowledge gap that exists in costing unsafe abortion in Ethiopia, estimates were derived of the cost to the health system of providing post-abortion care (PAC). | 14 health facilities in Ethiopia. | Cost analysis | The average direct cost per client, across 5 types of abortion complications, was US $36.21. This total includes the cost of drugs, supplies, material, personnel time, out-of-pocket expenses. |
| [167] [Uganda] | To fill a gap in knowledge in the cost of unsafe abortion, this article presents estimates based on the research conducted in 2010 of the cost to the Ugandan health system of providing post-abortion care (PAC). | 39 health facilities in Uganda | Cost analysis | The average annual PAC cost per client, across five types of abortion complications, was $131. This total includes the cost of drugs, supplies, material, personnel time, out-of-pocket expenses, and direct non-medical costs in the form of overhead and capital costs. |
| [168] [United States] | To understand abortion care in relation to providers of women’s health care | Pregnant people seeking abortions across three clinics in two states in the U.S. Heartland | Qualitative interviews | In the case study of one respondent [pseudonym Cheryl]:  “At the time of the interview, she had already been billed $11,000 for the hospital care for the mass while she was trying to locate an abortion provider. She was acutely aware of the increasing costs related to her refusal: ‘‘I spent probably seventy- five, eighty-five dollars just on medicine for vomiting and pain that I never would have had to have.’’ In the end Cheryl’s expenses totaled over $40,000; Cheryl has no insurance.” |
| [169] [Thailand] | To examine the effects of the current laws through the experiences of women who have undergone illegal abortions. | Women of reproductive age | Qualitative | Most rural women cannot afford or have difficulty accessing safe private abortion services. Women’s risks are stratified along economic lines. |
| [170] [Israel] | This study uses data from the 1974-75 Israel Fertility Survey and the 1987-88 Study of Fertility and Family Formation to examine the changing determinants of abortion among Jewish women in Israel. | Women of reproductive age in Israel | Regression analysis | In cases where government agencies do not cover the costs of the procedure, the woman pays the equivalent of US$250-400, depending on the hospital, for the procedure and associated care. Abortion is widely available in Israel, and 95% of Israeli women have access to moderately priced abortion. |
| [171] [United States, France] | To examine the commercial, political, regulatory, and legislative history of the introduction of mifepristone / misoprostol in France and the United States. | n/a | Review | In the United States, a large majority of women (74%) pay for their abortions with their own money or with funds they obtain from their partners, family or others. Most abortion costs are paid out-of-pocket. The insurance situation in the United States has important implications for the use and accessibility of mifepristone. On the one hand, because few women use insurance to cover the procedure, insurance companies exert little influence over practice patterns or standards of care compared to other surgical or reproductive health procedures; clinics and providers have been free to develop innovative service models for provision of the service that ultimately may have reduced the overall cost of the procedure. On the other hand, the price of the procedure has a wide range. The adjusted cost of providing medical abortion care varies significantly depending upon the practice model used (from $252 to $460 per abortion, median $351). Consequently, the method may be more or less accessible or a more or less attractive alternative to surgical abortion depending upon the practice and pricing model in place. |
| [172] [Nepal] | To review abortion care in Nepal 15 years after it was legalized. | n/a | Review | The landmark 2009 Supreme Court decision in Lakshmi Dhikta v. Nepal centered on a poor, rural woman who was forced to give birth to her sixth child due to her inability to afford the required fees for an abortion (approximately US$20). In the past, government policies mandated a small fee—ranging from 800 to 1200 Nepali rupees (US$8 to 12)—for abortion. This cost did not include pain medications, antibiotics, gloves, or syringes. Abortion was purposely separated from the package of free maternal care services, out of concern that inclusion may promote abortion as a method of contraception. While the landmark 2009 Supreme Court decision established the legal framework for the government to mandate free and accessible abortion services in the public sector, there was no policy to implement safe abortion services until the passage of the Safe Abortion Service Guidelines of 2016. Under these guidelines, all government facilities should provide free abortion services. However, the provider reimbursement scheme outlined in the guidelines is less profitable for providers than it was when women paid out of pocket. |
| [173] [Iran] | To calculate total and out of pocket inpatient costs for seven pregnancy complications including preeclampsia, intrauterine growth restriction (IUGR), abortion, ante-partum hemorrhage, preterm delivery, premature rupture of membranes and post-dated pregnancy | Teaching hospitals in Tehran (n=2) | Mixed methods: descriptive analysis and analysis of variance test | Patients receiving treatment for abortion complications had a mean expenditure of 370.52 USD. They paid 148.77 USD out of pocket. |
| [174] [Latin America] | To summarize the findings of a literature review on women’s experiences with medical abortion in Latin American countries where voluntary abortion is illegal | Studies on women’s experiences with medical abortion in Latin American countries where voluntary abortion is illegal | Literature review | Prices vary across country contexts and are often unaffordable: women report borrowing money from friends and relatives, asking for their salary in advance, working overtime or selling valuable objects.  The internet was a frequent source of access and where men’s involvement existed it was frequently via economic contributions. |

1. Aantjes CJ, Gilmoor A, Syurina EV, Crankshaw TL. The status of provision of post abortion care services for women and girls in Eastern and Southern Africa: a systematic review. Contraception. 2018;98(2):77-88. doi: 10.1016/j.contraception.2018.03.014. PubMed PMID: 130502138. Language: English. Entry Date: In Process. Revision Date: 20180707. Publication Type: Article.

2. Agadjanian V. Is "Abortion Culture" Fading in the Former Soviet Union? Views about Abortion and Contraception in Kazakhstan. Studies in Family Planning. 2002;33(3):237-48.

3. Ahmed S, Islam A, Khanum PA, Barkat e K. Induced abortion: What's happening in rural Bangladesh. Reproductive Health Matters. 1999;7(14):19-29. doi: <https://doi.org/10.1016/S0968-8080(99)90003-4>.

4. Aiken A, Gomperts R, Trussell J. Experiences and characteristics of women seeking and completing at-home medical termination of pregnancy through online telemedicine in Ireland and Northern Ireland: a population-based analysis. BJOG : an international journal of obstetrics and gynaecology. 2017;124(8):1208-15. Epub 2016/10/18. doi: 10.1111/1471-0528.14401. PubMed PMID: 27748001; PubMed Central PMCID: PMCPMC5393954.

5. Aiken ARA, Guthrie KA, Schellekens M, Trussell J, Gomperts R. Barriers to accessing abortion services and perspectives on using mifepristone and misoprostol at home in Great Britain. Contraception. 2018;97(2):177-83. doi: <https://doi.org/10.1016/j.contraception.2017.09.003>.

6. Appiah-Agyekum NN. Medical abortions among university students in Ghana: implications for reproductive health education and management. Int J Womens Health. 2018;10:515-22. doi: 10.2147/ijwh.s160297. PubMed PMID: WOS:000443766800001.

7. Austin N, Harper S. Assessing the impact of TRAP laws on abortion and women's health in the USA: a systematic review. BMJ sexual & reproductive health. 2018;44(2):128-34. Epub 2018/06/21. doi: 10.1136/bmjsrh-2017-101866. PubMed PMID: 29921636.

8. Ayanore MA, Pavlova M, Biesma R, Groot W. Stakeholder's experiences, expectations and decision making on reproductive care: An ethnographic study of three districts in northern Ghana. PloS one. 2017;12(11):e0186908. Epub 2017/11/02. doi: 10.1371/journal.pone.0186908. PubMed PMID: 29091916; PubMed Central PMCID: PMCPMC5665529.

9. Azize-Vargas Y, Avilés LA. Abortion in Puerto Rico: The limits of colonial legality. Reproductive Health Matters. 1997;5(9):56-65. doi: <https://doi.org/10.1016/S0968-8080(97)90006-9>.

10. Babigumira JB, Stergachis A, Veenstra DL, Gardner JS, Ngonzi J, Mukasa-Kivunike P, et al. Estimating the costs of induced abortion in Uganda: a model-based analysis. BMC Public Health. 2011;11(1):904-. PubMed PMID: 104498092. Language: English. Entry Date: 20130222. Revision Date: 20150711. Publication Type: Journal Article.

11. Baird B. Medical abortion in Australia: a short history. Reproductive Health Matters. 2015;23(46):169-76. doi: <https://doi.org/10.1016/j.rhm.2015.10.002>.

12. Baird B. Decriminalization and Women's Access to Abortion in Australia. Health & Human Rights: An International Journal. 2017;19(1):197-208.

13. Banerjee SK, Andersen K. Exploring the pathways of unsafe abortion in Madhya Pradesh, India. Global Public Health. 2012;7(8):882-96. doi: 10.1080/17441692.2012.702777. PubMed PMID: WOS:000308031400007.

14. Banerjee SK, Andersen KL, Warvadekar J. Pathways and consequences of unsafe abortion: a comparison among women with complications after induced and spontaneous abortions in Madhya Pradesh, India. International Journal of Gynecology & Obstetrics. 2012;118:S113-20. doi: 10.1016/S0020-7292(12)60009-5.

15. Banerjee SK, Andersen KL, Warvadekar J, Aich P, Rawat A, Upadhyay B. How prepared are young, rural women in India to address their sexual and reproductive health needs? a cross-sectional assessment of youth in Jharkhand. Reproductive Health. 2015;12:1-10. doi: 10.1186/s12978-015-0086-8. PubMed PMID: 110629005. Language: English. Entry Date: 20151105. Revision Date: 20151107. Publication Type: Article.

16. Banerjee SK, Kumar R, Warvadekar J, Manning V, Andersen KL. An exploration of the socio-economic profile of women and costs of receiving abortion services at public health facilities of Madhya Pradesh, India. BMC health services research. 2017;17(1):223. Epub 2017/03/23. doi: 10.1186/s12913-017-2159-6. PubMed PMID: 28320385; PubMed Central PMCID: PMCPMC5360007.

17. Barua A, Apte H. Quality of Abortion Care: Perspectives of Clients and Providers in Jharkhand. Economic and Political Weekly. 2007;42(48):71-80.

18. Baum SE, White K, Hopkins K, Potter JE, Grossman D. Women's Experience Obtaining Abortion Care in Texas after Implementation of Restrictive Abortion Laws: A Qualitative Study. PloS one. 2016;11(10):e0165048. Epub 2016/10/27. doi: 10.1371/journal.pone.0165048. PubMed PMID: 27783708; PubMed Central PMCID: PMCPMC5082726 case Whole Woman's Health v. Hellerstedt. Dr. Grossman was not compensated for his testimony; Dr. Hopkins was compensated. Lead author Sarah Baum is affiliated with Ibis Reproductive Health which is "an international nonprofit organization with a mission to improve women's reproductive autonomy, choices, and health worldwide". All authors are affiliated with the Texas Policy Evaluation Project (TxPEP), whose purpose is to document and evaluate the impact of reproductive health legislation passed by the Texas Legislature. Both Ibis Reproductive Health and TxPEP conduct rigorous research to assess the impact of reproductive health policies. This does not alter our adherence to PLOS ONE policies on sharing data and materials.

19. Baxerres C, Boko I, Konkobo A, Ouattara F, Guillaume A. Abortion in two francophone African countries: a study of whether women have begun to use misoprostol in Benin and Burkina Faso. Contraception. 2018;97(2):130-6. doi: <https://doi.org/10.1016/j.contraception.2017.10.011>.

20. Belton S, Whittaker A. Kathy Pan, sticks and pummelling: Techniques used to induce abortion by Burmese women on the Thai border. Social Science & Medicine. 2007;65(7):1512-23. doi: <https://doi.org/10.1016/j.socscimed.2007.05.046>.

21. Benson J, Nicholson LA, Gaffikin L, Kinoti SN. Complications of unsafe abortion in sub-Saharan Africa: a review. Health policy and planning. 1996;11(2):117-31. Epub 1996/05/07. PubMed PMID: 10158454.

22. Berer M. Making abortions safe: a matter of good public health policy and practice. Bulletin of the World Health Organization. 2000;78(5):580-92. Epub 2000/06/22. PubMed PMID: 10859852; PubMed Central PMCID: PMCPMC2560758.

23. Berer M. Medical abortion: issues of choice and acceptability. Reproductive Health Matters. 2005;13(26):25-34. PubMed PMID: 106392061. Language: English. Entry Date: 20060203. Revision Date: 20150711. Publication Type: Journal Article.

24. Bessett D, Gorski K, Jinadasa D, Ostrow M, Peterson MJ. Out of Time and Out of Pocket: Experiences of Women Seeking State-Subsidized Insurance for Abortion Care in Massachusetts. Women's Health Issues. 2011;21(3, Supplement):S21-S5. doi: <https://doi.org/10.1016/j.whi.2011.02.008>.

25. Billings DL, Benson J. Postabortion care in Latin America: policy and service recommendations from a decade of operations research. Health policy and planning. 2005;20(3):158-66. Epub 2005/04/21. doi: 10.1093/heapol/czi020. PubMed PMID: 15840631.

26. Blanchard K, Meadows JL, Gutierrez HR, Hannum CPS, Douglas-Durham EF, Dennis AJ. Mixed-methods investigation of women’s experiences with second-trimester abortion care in the Midwest and Northeast United States. Contraception. 2017;96(6):401-10. doi: <https://doi.org/10.1016/j.contraception.2017.08.008>.

27. Bloomer F, O'Dowd K. Restricted access to abortion in the Republic of Ireland and Northern Ireland: exploring abortion tourism and barriers to legal reform. Culture, Health & Sexuality. 2014;16(4):366-80. doi: 10.1080/13691058.2014.886724.

28. Brack CE, Rochat RW, Bernal OA. It's a Race Against the Clock: A Qualitative Analysis of Barriers to Legal Abortion in Bogot, Colombia. International Perspectives on Sexual and Reproductive Health. 2017;43(4):173-82.

29. Brown RW, Jewell RT. The Impact of Provider Availability on Abortion Demand. Contemporary Economic Policy. 1996;14(2):95-106. doi: <http://onlinelibrary.wiley.com/journal/10.1111/%28ISSN%291465-7287>. PubMed PMID: 0384757.

30. Calkin S. Towards a political geography of abortion. Political Geography. 2019;69:22-9. doi: <https://doi.org/10.1016/j.polgeo.2018.11.006>.

31. Campbell M, Sahin-Hodoglugil NN, Potts M. Barriers to Fertility Regulation: A Review of the Literature. Studies in Family Planning. 2006;37(2):87-98.

32. Cano JK, Foster AM. "They made me go through like weeks of appointments and everything": Documenting women's experiences seeking abortion care in Yukon territory, Canada. Contraception. 2016;94(5):489-95. doi: 10.1016/j.contraception.2016.06.015. PubMed PMID: WOS:000386742300012.

33. Casas L, Vivaldi L. Abortion in Chile: the practice under a restrictive regime. Reproductive Health Matters. 2014;22(44):70-81. doi: <https://doi.org/10.1016/S0968-8080(14)44811-0>.

34. Casas-Becerra L. Women prosecuted and imprisoned for abortion in Chile. Reproductive Health Matters. 1997;5(9):29-36. doi: <https://doi.org/10.1016/S0968-8080(97)90003-3>.

35. Chełstowska A. Stigmatisation and commercialisation of abortion services in Poland: turning sin into gold. Reproductive Health Matters. 2011;19(37):98-106. doi: 10.1016/S0968-8080(11)37548-9. PubMed PMID: 104894879. Language: English. Entry Date: 20110617. Revision Date: 20150711. Publication Type: Journal Article.

36. Chor J, Garcia-Ricketts S, Young D, Hebert LE, Hasselbacher LA, Gilliam ML. Well-woman Care Barriers and Facilitators of Low-income Women Obtaining Induced Abortion after the Affordable Care Act. Women's Health Issues. 2018;28(5):387-92. doi: <https://doi.org/10.1016/j.whi.2018.03.009>.

37. Chunuan S, Kosunvanna S, Sripotchanart W, Lawantra J, Lawantrakul J, Pattrapakdikul U, et al. Characteristics of Abortions in Southern Thailand. Pacific Rim International Journal of Nursing Research. 2012;16(2):97-112.

38. Coast E, Murray SF. “These things are dangerous”: Understanding induced abortion trajectories in urban Zambia. Social Science & Medicine. 2016;153:201-9. doi: 10.1016/j.socscimed.2016.02.025.

39. Coast E, Norris AH, Moore AM, Freeman E. Trajectories of women's abortion-related care: A conceptual framework. Social Science & Medicine. 2018;200:199-210. doi: <https://doi.org/10.1016/j.socscimed.2018.01.035>.

40. Cockrill K, Weitz TA. Abortion patients' perceptions of abortion regulation. Women's health issues : official publication of the Jacobs Institute of Women's Health. 2010;20(1):12-9. Epub 2010/02/04. doi: 10.1016/j.whi.2009.08.005. PubMed PMID: 20123172.

41. Cohen J, Ortiz O, Llaguno SE, Goodyear L, Billings D, Martinez I. Reaching Women with Instructions on Misoprostol Use in a Latin American Country. Reproductive Health Matters. 2005;13(26):84-92. doi: <https://doi.org/10.1016/S0968-8080(05)26202-X>.

42. Coles MS, Makino KK, Stanwood NL, Dozier A, Klein JD. How Are Restrictive Abortion Statutes Associated With Unintended Teen Birth? Journal of Adolescent Health. 2010;47(2):160-7. doi: <https://doi.org/10.1016/j.jadohealth.2010.01.003>.

43. Colman S, Joyce T. Regulating Abortion: Impact on Patients and Providers in Texas. Journal of Policy Analysis and Management. 2011;30(4):775-97. doi: <http://onlinelibrary.wiley.com/journal/10.1002/%28ISSN%291520-6688/issues>. PubMed PMID: 1263071.

44. Comendant R. A project to improve the quality of abortion services in Moldova. Reproductive Health Matters. 2005;13(26):93-100.

45. Cooper D, Dickson K, Blanchard K, Cullingworth L, Mavimbela N, von Mollendorf C, et al. Medical abortion: the possibilities for introduction in the public sector in South Africa [corrected] [published erratum appears in REPROD HEALTH MATTERS 2006 May;14(27):5]. Reproductive Health Matters. 2005;13(26):35-43.

46. Creinin MD, Shore E, Balasubramanian S, Harwood B. The true cost differential between mifepristone and misoprostol and misoprostol-alone regimens for medical abortion. Contraception. 2005;71(1):26-30. doi: <https://doi.org/10.1016/j.contraception.2004.07.011>.

47. Crighton E, Ebert M. RU 486 and abortion practices in Europe: From legalization to access. Women Polit. 2002;24(3):13-33. doi: 10.1300/J014v24n03_02. PubMed PMID: WOS:000180540600002.

48. David HP, Baban A. Women's health and reproductive rights: Romanian experience. Patient Education & Counseling. 1996;28(3):235-45.

49. Davey C. Sexual and Reproductive Health and Rights in the United Kingdom at ICPD+10. Reproductive Health Matters. 2005;13(25):81-7. doi: <https://doi.org/10.1016/S0968-8080(05)25182-0>.

50. de Bruyn M. Safe Abortion for HIV-Positive Women with Unwanted Pregnancy: A Reproductive Right. Reproductive Health Matters. 2003;11(22):152-61. doi: <https://doi.org/10.1016/S0968-8080(03)02297-3>.

51. Dennis A, Blanchard K. A Mystery Caller Evaluation of Medicaid Staff Responses about State Coverage of Abortion Care. Women's Health Issues. 2012;22(2):e143-e8. doi: <https://doi.org/10.1016/j.whi.2011.11.001>.

52. Dennis A, Manski R, Blanchard K. Does Medicaid Coverage Matter? A Qualitative Multi-State Study of Abortion Affordability for Low-income Women. Journal of Health Care for the Poor & Underserved. 2014;25(4):1571-85. doi: 10.1353/hpu.2014.0151.

53. Dennis A, Manski R, Blanchard K. A Qualitative Exploration of Low-Income Women's Experiences Accessing Abortion in Massachusetts. Women's Health Issues. 2015;25(5):463-9. doi: <https://doi.org/10.1016/j.whi.2015.04.004>.

54. Díaz-Olavarrieta C, Cravioto VM, Villalobos A, Deeb-Sossa N, García L, García SG. Mexico City’s Legal Abortion Program: health workers’ experiences. Revista Panamericana de Salud Publica. 2012;32(6):399-404.

55. Diniz SG, d'Oliveira AFPL, Lansky S. Equity and women's health services for contraception, abortion and childbirth in Brazil. Reproductive Health Matters. 2012;20(40):94-101. doi: <https://doi.org/10.1016/S0968-8080(12)40657-7>.

56. Dobie SA, Gober L, Rosenblatt RA. Family planning service provision in rural areas: a survey in Washington State. Family planning perspectives. 1998;30(3):139-42, 47. Epub 1998/06/23. PubMed PMID: 9635263.

57. Doran F, Hornibrook J. Rural New South Wales women's access to abortion services: highlights from an exploratory qualitative study. The Australian journal of rural health. 2014;22(3):121-6. Epub 2014/07/22. doi: 10.1111/ajr.12096. PubMed PMID: 25039846.

58. Doran F, Nancarrow S. Barriers and facilitators of access to first-trimester abortion services for women in the developed world: a systematic review. Journal of Family Planning & Reproductive Health Care. 2015;41(3):170-80. doi: 10.1136/jfprhc-2013-100862. PubMed PMID: 109585078. Language: English. Entry Date: 20150923. Revision Date: 20170104. Publication Type: journal article.

59. Doran FM, Hornibrook J. Barriers around access to abortion experienced by rural women in New South Wales, Australia. Rural & Remote Health. 2016;16(1):3538-49.

60. Dragoman M, Davis A. Abortion care for adolescents. Clinical Obstetrics & Gynecology. 2008;51(2):281-9.

61. Duggal R. The political economy of abortion in India: cost and expenditure patterns. Reproductive Health Matters. 2004;12:130-7.

62. Duggal R, Ramachandran V. The Abortion Assessment Project-India: Key Findings and Recommendations. Reproductive Health Matters. 2004;12(24):122-9.

63. Dzuba IG, Winikoff B, Pena M. Medical abortion: a path to safe, high-quality abortion care in Latin America and the Caribbean. The European journal of contraception & reproductive health care : the official journal of the European Society of Contraception. 2013;18(6):441-50. Epub 2013/09/17. doi: 10.3109/13625187.2013.824564. PubMed PMID: 24033184.

64. Ely GE, Hales T, Jackson DL, Bowen EA, Maguin E, Hamilton G. A trauma-informed examination of the hardships experienced by abortion fund patients in the United States. Health Care for Women International. 2017a;38(11):1133-51. doi: 10.1080/07399332.2017.1367795.

65. Ely GE, Hales T, Jackson DL, Maguin E, Hamilton G. The undue burden of paying for abortion: An exploration of abortion fund cases. Social Work in Health Care. 2017b;56(2):99-114. doi: 10.1080/00981389.2016.1263270.

66. Ely GE, Hales TW, Jackson DL, Maguin E, Hamilton G. Where are They from and How Far Must They Go? Examining Location and Travel Distance in U.S. Abortion Fund Patients. International Journal of Sexual Health. 2017c;29(4):313-24. doi: 10.1080/19317611.2017.1316809. PubMed PMID: 126206284. Language: English. Entry Date: 20171123. Revision Date: 20171123. Publication Type: Article.

67. Ely GE, Hales TW, Jackson DL. A cross-cultural exploration of abortion fund patients in the USA and the Republic of Ireland, Northern Ireland and the Isle of Man. Culture, Health & Sexuality. 2018;20(5):560-73. doi: 10.1080/13691058.2017.1361550. PubMed PMID: 129343735. Language: English. Entry Date: 20180906. Revision Date: 20180906. Publication Type: Article. Journal Subset: Allied Health.

68. Esia-Donkon K, Darteh EKM, Blemano H, Asare H. Who Cares? Pre and Post Abortion Experiences among Young Females in Cape Coast Metropolis, Ghana. African Journal of Reproductive Health / La Revue Africaine de la Santé Reproductive. 2015;19(2):43-51.

69. Felkey AJ, Lybecker KM. Utilization of oral contraception: The impact of direct and indirect restrictions on access to abortion. The Social Science Journal. 2014;51(1):44-56. doi: <https://doi.org/10.1016/j.soscij.2013.07.016>.

70. Felkey AJ, Lybecker KM. Do Restrictions Beget Responsibility? The Case of U.S. Abortion Legislation. The American Economist. 2018;63(1):59-70. doi: 10.1177/0569434517692972.

71. Finer LB, Frohwirth LF, Dauphinee LA, Singh S, Moore AM. Timing of steps and reasons for delays in obtaining abortions in the United States. Contraception. 2006;74(4):334-44. doi: <https://doi.org/10.1016/j.contraception.2006.04.010>.

72. Finer LB, Frohwirth LF, Dauphinee LA, Singh S, Moore AM. Reasons U.S. Women Have Abortions: Quantitative and Qualitative Perspectives. Perspectives on Sexual and Reproductive Health. 2005;37(3):110-8.

73. Font-Ribera L, Perez G, Espelt A, Salvador J, Borrell C. Determinants of induced abortion delay. Gac Sanit. 2009;23(5):415-9. doi: 10.1016/j.gaceta.2008.08.001. PubMed PMID: WOS:000270411100010.

74. Foster DG, Jackson RA, Cosby K, Weitz TA, Darney PD, Drey EA. Predictors of delay in each step leading to an abortion. Contraception. 2008;77(4):289-93. doi: <https://doi.org/10.1016/j.contraception.2007.10.010>.

75. Foster DG, Kimport K. Who Seeks Abortions at or After 20 Weeks? Perspectives on Sexual & Reproductive Health. 2013;45(4):210-8. doi: 10.1363/4521013.

76. Foster AM, LaRoche KJ, El-Haddad J, DeGroot L, El-Mowafi IM. "If I ever did have a daughter, I wouldn't raise her in New Brunswick:" exploring women's experiences obtaining abortion care before and after policy reform. Contraception. 2017;95(5):477-84. doi: 10.1016/j.contraception.2017.02.016.

77. Freeman E, Coast E, Murray SF. Men's Roles in Women's Abortion Trajectories in Urban Zambia. International Perspectives on Sexual and Reproductive Health. 2017;43(2):89-98. doi: 10.1363/43e4017.

78. French V, Anthony R, Souder C, Geistkemper C, Drey E, Steinauer J. Influence of clinician referral on Nebraska women's decision-to-abortion time. Contraception. 2016;93(3):236-43. doi: 10.1016/j.contraception.2015.10.005. PubMed PMID: 112473204. Language: English. Entry Date: 20161123. Revision Date: 20180703. Publication Type: journal article.

79. Friedman J, Saavedra-Avendaño B, Schiavon R, Alexander L, Sanhueza P, Rios-Polanco R, et al. Quantifying disparities in access to public-sector abortion based on legislative differences within the Mexico City Metropolitan Area. Contraception. 2018. doi: <https://doi.org/10.1016/j.contraception.2018.11.012>.

80. Gallo MF, Nghia NC. Real life is different: a qualitative study of why women delay abortion until the second trimester in Vietnam. Social Science & Medicine. 2007;64(9):1812-22.

81. Ganatra B, Hirve S. Induced Abortions Among Adolescent Women in Rural Maharashtra, India. Reproductive Health Matters. 2002;10(19):76-85. doi: <https://doi.org/10.1016/S0968-8080(02)00016-2>.

82. Ganatra B, Manning V, Pallipamulla SP. Availability of medical abortion pills and the role of chemists: a study from Bihar and Jharkhand, India. Reproductive Health Matters. 2005;13(26):65-74.

83. Gerdts C, Fuentes L, Grossman D, White K, Keefe-Oates B, Baum SE, et al. Impact of Clinic Closures on Women Obtaining Abortion Services After Implementation of a Restrictive Law in Texas. American Journal of Public Health. 2016;106(5):857-64. doi: 10.2105/AJPH.2016.303134. PubMed PMID: 114349499. Language: English. Entry Date: 20160511. Revision Date: 20181211. Publication Type: Article.

84. Gerdts C, DeZordo S, Mishtal J, Barr-Walker J, Lohr PA. Experiences of women who travel to England for abortions: an exploratory pilot study. European Journal of Contraception & Reproductive Health Care. 2016;21(5):401-7. doi: 10.1080/13625187.2016.1217325. PubMed PMID: 118174771. Language: English. Entry Date: 20170506. Revision Date: 20180430. Publication Type: journal article.

85. Gerdts C, Raifman S, Daskilewicz K, Momberg M, Roberts S, Harries J. Women's experiences seeking informal sector abortion services in Cape Town, South Africa: a descriptive study. BMC Women's Health. 2017;17:1-10. doi: 10.1186/s12905-017-0443-6. PubMed PMID: 125443610. Language: English. Entry Date: 20180723. Revision Date: 20180920. Publication Type: journal article. Journal Subset: Biomedical.

86. Girard F, Nowicka W. Clear and Compelling Evidence: The Polish Tribunal on Abortion Rights. Reproductive Health Matters. 2002;10(19):22-30. doi: <https://doi.org/10.1016/S0968-8080(02)00023-X>.

87. Grindlay K, Lane K, Grossman D. Women's and Providers' Experiences with Medical Abortion Provided Through Telemedicine: A Qualitative Study. Women's Health Issues. 2013;23(2):e117-e22. doi: <https://doi.org/10.1016/j.whi.2012.12.002>.

88. Gresh A, Maharaj P. A qualitative assessment of the acceptability and potential demand for medical abortion among university students in Durban, South Africa. The European journal of contraception & reproductive health care : the official journal of the European Society of Contraception. 2011;16(2):67-75. Epub 2011/01/26. doi: 10.3109/13625187.2010.546534. PubMed PMID: 21261553.

89. Grossman D, Ellertson C, Grimes DA, Walker D. Routine follow-up visits after first-trimester induced abortion. Obstetrics and gynecology. 2004;103(4):738-45. Epub 2004/03/31. doi: 10.1097/01.aog.0000115511.14004.19. PubMed PMID: 15051567.

90. Grossman D, Baum S, Fuentes L, White K, Hopkins K, Stevenson A, et al. Change in abortion services after implementation of a restrictive law in Texas. Contraception. 2014;90(5):496-501. doi: <https://doi.org/10.1016/j.contraception.2014.07.006>.

91. Guttmacher S, Kapadia F, Jim Te Water N, de Pinho H. Abortion Reform in South Africa: A Case Study of the 1996 Choice on Termination of Pregnancy Act. International Family Planning Perspectives. 1998;24(4):191-4. doi: 10.2307/2991980.

92. Henshaw RC, Naji SA, Russell IT, Templeton AA. A prospective economic evaluation comparing medical abortion (using mifepristone and gemeprost) and surgical vacuum aspiration. British Journal of Family Planning. 1994;20(3):64-8. PubMed PMID: 24328058.

93. Henshaw SK. Factors hindering access to abortion services. Family Planning Perspectives. 1995;27(2):54-87. PubMed PMID: 106100315. Language: English. Entry Date: 20081219. Revision Date: 20150711. Publication Type: Journal Article. Journal Subset: Biomedical.

94. Henshaw SK, Finer LB. The accessibility of abortion services in the United States, 2001. Perspectives on Sexual & Reproductive Health. 2003;35(1):16-24. PubMed PMID: 106822821. Language: English. Entry Date: 20050507. Revision Date: 20150820. Publication Type: Journal Article.

95. Henshaw SK, Adewole I, Singh S, Oye-Adeniran B, Hussain R, Bankole A. Severity and cost of unsafe abortion complications treated in Nigerian hospitals. International Family Planning Perspectives. 2008;34(1):40-50.

96. Htay TT, Sauvarin J, Khan S. Integration of Post-Abortion Care: The Role of Township Medical Officers and Midwives in Myanmar. Reproductive Health Matters. 2003;11(21):27-36. doi: <https://doi.org/10.1016/S0968-8080(03)02170-0>.

97. Hulme-Chambers A, Temple-Smith M, Davidson A, Coelli L, Orr C, Tomnay JE. Australian women’s experiences of a rural medical termination of pregnancy service: A qualitative study. Sexual & Reproductive Healthcare. 2018;15:23-7. doi: <https://doi.org/10.1016/j.srhc.2017.11.008>.

98. Hung SL. Access to safe and legal abortion for teenage women from deprived backgrounds in Hong Kong. Reproductive Health Matters. 2010;18(36):102-10. doi: 10.1016/S0968-8080(10)36527-X.

99. Ilboudo PGC, Greco G, Sundby J, Torsvik G. Costs and consequences of abortions to women and their households: a cross-sectional study in Ouagadougou, Burkina Faso. Health Policy & Planning. 2015;30(4):500-7. doi: 10.1093/heapol/czu025.

100. Izugbara CO, Ukwayi JK. The clientele of traditional birth homes in rural southeastern Nigeria. Health Care for Women International. 2003;24(3):177-92. PubMed PMID: 106843171. Language: English. Entry Date: 20030627. Revision Date: 20150818. Publication Type: Journal Article.

101. Izugbara CO, Egesa C, Okelo R. ‘High profile health facilities can add to your trouble’: Women, stigma and un/safe abortion in Kenya. Social Science & Medicine. 2015;141:9-18. doi: <https://doi.org/10.1016/j.socscimed.2015.07.019>.

102. Janiak E, Kawachi I, Goldberg A, Gottlieb B. Abortion barriers and perceptions of gestational age among women seeking abortion care in the latter half of the second trimester. Contraception. 2014;89(4):322-7. doi: <https://doi.org/10.1016/j.contraception.2013.11.009>.

103. Jejeebhoy SJ, Kalyanwala S, Zavier AJF, Kumar R, Jha N. Experience seeking abortion among unmarried young women in Bihar and Jharkhand, India: delays and disadvantages. Reproductive Health Matters. 2010;18(35):163-74. doi: <https://doi.org/10.1016/S0968-8080(10)35504-2>.

104. Jerman J, Frohwirth L, Kavanaugh ML, Blades N. Barriers to Abortion Care and Their Consequences For Patients Traveling for Services: Qualitative Findings from Two States. Perspectives on Sexual & Reproductive Health. 2017;49(2):95-102. doi: 10.1363/psrh.12024.

105. Jewell RT, Brown RW. An economic analysis of abortion: the effect of travel cost on teenagers. The Social Science Journal. 2000;37(1):113-24. doi: <https://doi.org/10.1016/S0362-3319(99)00063-4>.

106. Johnston HB, Ved R, Lyall N, Agarwal K. Where do rural women obtain postabortion care? The case of Uttar Pradesh, India. International Family Planning Perspectives. 2003;29(4):182-7. PubMed PMID: 106745909. Language: English. Entry Date: 20040618. Revision Date: 20151008. Publication Type: Journal Article.

107. Jones BS, Weitz TA. Legal barriers to second-trimester abortion provision and public health consequences. American Journal of Public Health. 2009;99(4):623-30. doi: 10.2105/AJPH.2007.127530.

108. Jones RK, Ingerick M, Jerman J. Differences in Abortion Service Delivery in Hostile, Middle-ground, and Supportive States in 2014. Women's Health Issues. 2018;28(3):212-8. doi: 10.1016/j.whi.2017.12.003. PubMed PMID: 129626512. Language: English. Entry Date: 20180522. Revision Date: 20180522. Publication Type: Article.

109. Jones RK, Upadhyay UD, Weitz TA. At What Cost? Payment for Abortion Care by U.S. Women. Women's Health Issues. 2013;23(3):e173-8. doi: 10.1016/j.whi.2013.03.001.

110. Kacanek D, Dennis A, Miller K, Blanchard K. Medicaid funding for abortion: providers' experiences with cases involving rape, incest and life endangerment. Perspectives on Sexual & Reproductive Health. 2010;42(2):79-86. doi: 10.1363/4207910. PubMed PMID: 105023417. Language: English. Entry Date: 20100730. Revision Date: 20150820. Publication Type: Journal Article.

111. Karasek D, Roberts SCM, Weitz TA. Abortion Patients' Experience and Perceptions of Waiting Periods: Survey Evidence before Arizona's Two-visit 24-hour Mandatory Waiting Period Law. Women's Health Issues. 2016;26(1):60-6. doi: <https://doi.org/10.1016/j.whi.2015.10.004>.

112. Kinoti SN, Gaffikin L, Benson J. How research can affect policy and programme advocacy: example from a three-country study on abortion complications in sub-Saharan Africa. East African medical journal. 2004;81(2):63-70. Epub 2004/05/06. PubMed PMID: 15125088.

113. Kishen M, Stedman Y. The role of Advanced Nurse Practitioners in the availability of abortion services. Best Practice & Research Clinical Obstetrics & Gynaecology. 2010;24(5):569-78. doi: <https://doi.org/10.1016/j.bpobgyn.2010.02.014>.

114. Lafaurie MM, Grossman D, Troncoso E, Billings DL, Chávez S. Women's Perspectives on Medical Abortion in Mexico, Colombia, Ecuador and Peru: A Qualitative Study. Reproductive Health Matters. 2005;13(26):75-83. doi: <https://doi.org/10.1016/S0968-8080(05)26199-2>.

115. Lalley JJ, Jelsema RD, Huzel PS, Ransom SB, Sokol RJ. The cost-effectiveness of misoprostol versus prostaglandin E2 for second trimester termination of pregnancy. Clinical Journal of Women's Health. 2001;1(3):149-56.

116. Le HH, Connolly MP, Yu JB, Pinchevsky Y, Steyn PS. The public health and economic consequences of unintended pregnancies in South Africa. Healthc Low-resource Settings. 2015;3(1):7. doi: 10.4081/hls.2015.5258. PubMed PMID: WOS:000358366800008.

117. Leone T, Coast E, Parmar D, Vwalika B. The individual level cost of pregnancy termination in Zambia: a comparison of safe and unsafe abortion. Health Policy & Planning. 2016;31(7):825-33. doi: 10.1093/heapol/czv138.

118. Limacher JJ, Daniel I, Isaacksz S, Payne GJ, Dunn S, Coyte PC, et al. Early Abortion in Ontario: Options and Costs. Journal of Obstetrics and Gynaecology Canada. 2006;28(2):142-8. doi: <https://doi.org/10.1016/S1701-2163(16)32065-5>.

119. Lince-Deroche N, Constant D, Harries J, Blanchard K, Sinanovic E, Grossman D. The costs of accessing abortion in South Africa: women's costs associated with second-trimester abortion services in Western Cape Province. Contraception. 2015;92(4):339-44. doi: 10.1016/j.contraception.2015.06.029. PubMed PMID: 109494869. Language: English. Entry Date: 20160703. Revision Date: 20180718. Publication Type: journal article. Journal Subset: Biomedical.

120. Lince-Deroche N, Fetters T, Sinanovic E, Blanchard K. Accessing medical and surgical first-trimester abortion services: women's experiences and costs from an operations research study in KwaZulu-Natal Province, South Africa. Contraception. 2017a;96(2):72-80. doi: 10.1016/j.contraception.2017.03.013.

121. Machungo F, Zanconato G, Bergstrom S. Socio-economic background, individual cost and hospital care expenditure in cases of illegal and legal abortion in Maputo. Health & Social Care in the Community. 1997;5(2):71-6.

122. Manouana M, Kadhel P, Koffi A, Janky E. Avortements illégaux par le misoprostol en Guadeloupe. J Gynecol Obstet Biol Reprod. 2013;42(2):137-42. doi: <https://doi.org/10.1016/j.jgyn.2012.10.006>.

123. Marlow HM, Wamugi S, Yegon E, Fetters T, Wanaswa L, Msipa-Ndebele S. Women’s perceptions about abortion in their communities: perspectives from western Kenya. Reproductive Health Matters. 2014;22(43):149-58. doi: 10.1016/S0968-8080(14)43758-3.

124. Medoff M. THE IMPACT OF STATE ABORTION POLICY ON THE PRICE OF AN ABORTION. Behav Soc Iss. 2015;24:56-67. doi: 10.5210/bsi.v.24i0.5635. PubMed PMID: WOS:000360132300004.

125. Messinger CJ, Mahmud I, Kanan S, Jahangir YT, Sarker M, Rashid SF. Utilization of mobile phones for accessing menstrual regulation services among low-income women in Bangladesh: a qualitative analysis. Reproductive Health. 2017;14:1-11. doi: 10.1186/s12978-016-0274-1. PubMed PMID: 120778721. Language: English. Entry Date: 20170123. Revision Date: 20190107. Publication Type: Article.

126. Moore AM, Dennis M, Anderson R, Bankole A, Abelson A, Greco G, et al. Comparing women's financial costs of induced abortion at a facility vs. seeking treatment for complications from unsafe abortion in Zambia. Reproductive Health Matters. 2018;26(52):138-50. doi: 10.1080/09688080.2018.1522195.

127. Murtagh C, Wells E, Raymond EG, Coeytaux F, Winikoff B. Exploring the feasibility of obtaining mifepristone and misoprostol from the internet. Contraception. 2018;97(4):287-91. doi: 10.1016/j.contraception.2017.09.016.

128. Murthy A, Creinin MD. Pharmacoeconomics of medical abortion: a review of cost in the United States, Europe and Asia. Expert opinion on pharmacotherapy. 2003;4(4):503-13. Epub 2003/04/02. doi: 10.1517/14656566.4.4.503. PubMed PMID: 12667113.

129. Mutua MM, Manderson L, Musenge E, Achia TNO. Policy, law and post-abortion care services in Kenya. PloS one. 2018;13(9):18. doi: 10.1371/journal.pone.0204240. PubMed PMID: WOS:000445329700031.

130. Mutungi AK, Wango EO, Rogo KO, Kimani VN, Karanja JG. Abortion: Behaviour of adolescents in two districts in Kenya. East Afr Med J. 1999;76(10):541-6. PubMed PMID: WOS:000087190400002.

131. Naghma e R. Cost of the treatment of complications of unsafe abortion in public hospitals. JPMA The Journal of the Pakistan Medical Association. 2011;61(2):169-72. Epub 2011/03/08. PubMed PMID: 21375169.

132. Nickson C, Smith AMA, Shelley JM. Travel undertaken by women accessing private Victorian pregnancy termination services. Australian & New Zealand Journal of Public Health. 2006;30(4):329-33. PubMed PMID: 105944760. Language: English. Entry Date: 20080125. Revision Date: 20150711. Publication Type: Journal Article.

133. Ogu R, Okonofua F, Hammed A, Okpokunu E, Mairiga A, Bako A, et al. Outcome of an intervention to improve the quality of private sector provision of postabortion care in northern Nigeria. International Journal of Gynecology & Obstetrics. 2012;118:S121-6. doi: 10.1016/S0020-7292(12)60010-1. PubMed PMID: 104299676. Language: English. Entry Date: 20130201. Revision Date: 20170104. Publication Type: journal article.

134. Ordinioha B, Brisibe S. Clandestine abortion in Port Harcourt: providers' motivations and experiences. Nigerian journal of medicine : journal of the National Association of Resident Doctors of Nigeria. 2008;17(3):291-5. Epub 2008/09/16. PubMed PMID: 18788255.

135. Ouedraogo R, Sundby J. Social determinants and access to induced abortion in Burkina Faso: from two case studies. Obstetrics and gynecology international. 2014;2014:402456. Epub 2014/05/03. doi: 10.1155/2014/402456. PubMed PMID: 24790605; PubMed Central PMCID: PMCPMC3976952.

136. Palma Manríquez I, Moreno Standen C, Álvarez Carimoney A, Richards A. Experience of clandestine use of medical abortion among university students in Chile: a qualitative study. Contraception. 2018;97(2):100-7. doi: <https://doi.org/10.1016/j.contraception.2017.09.008>.

137. Penfold S, Wendot S, Nafula I, Footman K. A qualitative study of safe abortion and post-abortion family planning service experiences of women attending private facilities in Kenya. Reproductive Health. 2018;15(1):N.PAG-N.PAG. doi: 10.1186/s12978-018-0509-4. PubMed PMID: 129271442. Language: English. Entry Date: 20180426. Revision Date: 20190107. Publication Type: Article.

138. Perrin E, Berthoud M, Pott M, Toledo Vera AG, Perrenoud D, Bianchi-Demicheli F. Clinical course in women undergoing termination of pregnancy within the legal time limit in French-speaking Switzerland. Swiss medical weekly. 2011;141:w13282. Epub 2011/10/20. doi: 10.4414/smw.2011.13282. PubMed PMID: 22009758.

139. Pheterson G, Azize Y. Abortion Practice in the Northeast Caribbean: “Just write down stomach pain”. Reproductive Health Matters. 2005;13(26):44-53. doi: <https://doi.org/10.1016/S0968-8080(05)26201-8>.

140. Pongsatha S, Morakot N, Tongsong T. Demographic characteristics of women with self use of misoprostol for pregnancy interruption attending Maharaj Nakorn Chiang Mai Hospital. J Med Assoc Thai. 2002;85(10):1074-80. Epub 2002/12/28. PubMed PMID: 12501898.

141. Potdar R, Fetters T, Phirun L. Initial loss of productive days and income among women seeking induced abortion in Cambodia. Journal of Midwifery & Women's Health. 2008;53(2):123-9. doi: 10.1016/j.jmwh.2007.06.010.

142. Prada E, Bankole A, Oladapo OT, Awolude OA, Adewole IF, Onda T. Maternal Near-Miss Due to Unsafe Abortion and Associate Short-Term Health and Socio-Economic Consequences in Nigeria. African Journal of Reproductive Health / La Revue Africaine de la Santé Reproductive. 2015;19(2):52-62.

143. Ramachandar L, Pelto PJ. Abortion Providers and Safety of Abortion: A Community-Based Study in a Rural District of Tamil Nadu, India. Reproductive Health Matters. 2004;12(24, Supplement):138-46. doi: <https://doi.org/10.1016/S0968-8080(04)24015-0>.

144. Roberts SCM, Gould H, Kimport K, Weitz TA, Foster DG. Out-of-Pocket Costs and Insurance Coverage for Abortion in the United States. Women's Health Issues. 2014;24(2):e211-8. doi: 10.1016/j.whi.2014.01.003.

145. Rossier C. Abortion: An Open Secret? Abortion and Social Network Involvement in Burkina Faso. Reproductive Health Matters. 2007;15(30):230-8. doi: <https://doi.org/10.1016/S0968-8080(07)30313-3>.

146. Schiavon R, Collado ME, Troncoso E, Soto Sánchez JE, Zorrilla GO, Palermo T. Characteristics of private abortion services in Mexico City after legalization. Reproductive Health Matters. 2010;18(36):127-35. doi: 10.1016/S0968-8080(10)36530-X.

147. Schuster S. Women's experiences of the abortion law in Cameroon: “What really matters”. Reproductive Health Matters. 2010;18(35):137-44. doi: <https://doi.org/10.1016/S0968-8080(10)35503-0>.

148. Sethna C, Doull M. Far From Home? A Pilot Study Tracking Women’s Journeys to a Canadian Abortion Clinic. Journal of Obstetrics and Gynaecology Canada. 2007;29(8):640-7. doi: <https://doi.org/10.1016/S1701-2163(16)32560-9>.

149. Sethna C, Doull M. Spatial disparities and travel to freestanding abortion clinics in Canada. Women's Studies International Forum. 2013;38:52-62. doi: <https://doi.org/10.1016/j.wsif.2013.02.001>.

150. Shankar M, Black KI, Goldstone P, Hussainy S, Mazza D, Petersen K, et al. Access, equity and costs of induced abortion services in Australia: a cross-sectional study. Australian & New Zealand Journal of Public Health. 2017;41(3):309-14. doi: 10.1111/1753-6405.12641.

151. Singh S. Global consequences of unsafe abortion. Women's health (London, England). 2010;6(6):849-60. Epub 2010/12/02. doi: 10.2217/whe.10.70. PubMed PMID: 21118043.

152. Sjostrand M, Quist V, Jacobson A, Bergstrom S, Rogo KO. Socio-economic client characteristics and consequences of abortion in Nairobi. East African medical journal. 1995;72(5):325-32. Epub 1995/05/01. PubMed PMID: 7555891.

153. Sjostrom S, Kopp Kallner H, Simeonova E, Madestam A, Gemzell-Danielsson K. Medical Abortion Provided by Nurse-Midwives or Physicians in a High Resource Setting: A Cost-Effectiveness Analysis. PloS one. 2016;11(6):e0158645. Epub 2016/07/01. doi: 10.1371/journal.pone.0158645. PubMed PMID: 27362270; PubMed Central PMCID: PMCPMC4928948.

154. Sommer MH. Abortion in late Imperial China: routine birth control or crisis intervention? Late imperial China = Ch'ing shih wen t'i. 2010;31(2):97-165. Epub 2011/02/19. PubMed PMID: 21328808.

155. Souza ZCSdN, Diniz NMF, Couto TM, Gesteira SMdA. Trajectory of women that performed a provoked abortion contained in the discourse of a clandestine procedure. Acta Paulista de Enfermagem. 2010;23(6):732-6. doi: 10.1590/S0103-21002010000600003.

156. Sundaram A, Vlassoff M, Mugisha F, Bankole A, Singh S, Amanya L, et al. Documenting the Individual- and Household-Level Cost of Unsafe Abortion in Uganda. International Perspectives on Sexual & Reproductive Health. 2013;39(4):174-84. doi: 10.1363/3917413.

157. Svanemyr J, Sundby J. The Social Context of Induced Abortions among Young Couples in Côte d'Ivoire. African Journal of Reproductive Health / La Revue Africaine de la Santé Reproductive. 2007;11(2):13-23. doi: 10.2307/25549712.

158. Thapa S, Poudel J, Padhye S. Triaging patients with post-abortion complications: a prospective study in Nepal. Journal of health, population, and nutrition. 2004;22(4):383-98. Epub 2005/01/25. PubMed PMID: 15663171.

159. Tousaw E, La RK, Arnott G, Chinthakanan O, Foster AM. “Without this program, women can lose their lives”: migrant women’s experiences with the Safe Abortion Referral Programme in Chiang Mai, Thailand. Reproductive Health Matters. 2017;25(51):58-68. doi: 10.1080/09688080.2017.1392220. PubMed PMID: 126920505. Language: English. Entry Date: 20171227. Revision Date: 20171227. Publication Type: Article.

160. Upadhyay UD, Johns NE, Combellick SL, Kohn JE, Keder LM, Roberts SCM. Comparison of Outcomes before and after Ohio's Law Mandating Use of the FDA-Approved Protocol for Medication Abortion: A Retrospective Cohort Study. PLoS Medicine. 2016;13(8):1-23. doi: 10.1371/journal.pmed.1002110. PubMed PMID: 117765205. Language: English. Entry Date: 20180725. Revision Date: 20180725. Publication Type: journal article.

161. Upadhyay UD, Johns NE, Meckstroth KR, Kerns JL. Distance Traveled for an Abortion and Source of Care After Abortion. Obstetrics & Gynecology. 2017;130(3):616-24. doi: 10.1097/AOG.0000000000002188. PubMed PMID: 124770936. Language: English. Entry Date: 20170919. Revision Date: 20170902. Publication Type: journal article. Journal Subset: Biomedical.

162. Upadhyay UD, Cartwright AF, Johns NE. Access to Medication Abortion Among California's Public University Students. Journal of Adolescent Health. 2018;63(2):249-52. doi: 10.1016/j.jadohealth.2018.04.009. PubMed PMID: 131430013. Language: English. Entry Date: 20180901. Revision Date: 20180901. Publication Type: Article. Journal Subset: Allied Health.

163. Van Bebber SL, Phillips KA, Weitz TA, Gould H, Stewart F. Patient costs for medication abortion: results from a study of five clinical practices. Women's Health Issues. 2006;16(1):4-13. PubMed PMID: 106452100. Language: English. Entry Date: 20060609. Revision Date: 20150711. Publication Type: Journal Article.

164. Visaria L, Barua A, Mistry R. Medical Abortion in India: Role of Chemists and Providers. Economic and Political Weekly. 2008;43(36):35-40.

165. Vlassoff M, Walker D, Shearer J, Newlands D, Singh S. Estimates of health care system costs of unsafe abortion in Africa and Latin America. International Perspectives on Sexual & Reproductive Health. 2009;35(3):114-21. doi: 10.1363/ipsrh.35.114.09. PubMed PMID: 105267647. Language: English. Entry Date: 20100212. Revision Date: 20150819. Publication Type: Journal Article.

166. Vlassoff M, Fetters T, Kumbi S, Singh S. The health system cost of postabortion care in Ethiopia. International Journal of Gynecology & Obstetrics. 2012;118:S127-33. doi: 10.1016/S0020-7292(12)60011-3. PubMed PMID: 104299678. Language: English. Entry Date: 20130201. Revision Date: 20170104. Publication Type: journal article.

167. Vlassoff M, Mugisha F, Sundaram A, Bankole A, Singh S, Amanya L, et al. The health system cost of post-abortion care in Uganda. Health Policy & Planning. 2014;29(1):56-66. doi: heapol/czs133. PubMed PMID: 103809528. Language: English. Entry Date: 20150508. Revision Date: 20150710. Publication Type: Journal Article. Journal Subset: Biomedical.

168. Weitz TA, Cockrill K. Abortion clinic patients' opinions about obtaining abortions from general women's health care providers. Patient Education & Counseling. 2010;81(3):409-14. doi: 10.1016/j.pec.2010.09.003. PubMed PMID: 104963200. Language: English. Entry Date: 20110401. Revision Date: 20150711. Publication Type: Journal Article.

169. Whittaker A. Reproducing inequalities: abortion policy and practice in Thailand. Women & Health. 2002;35(4):101-19. PubMed PMID: 106803363. Language: English. Entry Date: 20030207. Revision Date: 20150820. Publication Type: Journal Article.

170. Wilder EI. Socioeconomic and Cultural Determinants of Abortion among Jewish Women in Israel. European Journal of Population / Revue Européenne de Démographie. 2000;16(2):133-62.

171. Winikoff B, Hassoun D, Bracken H. Introduction and provision of medical abortion: a tale of two countries in which technology is necessary but not sufficient. Contraception. 2011;83(4):322-9. doi: <https://doi.org/10.1016/j.contraception.2010.08.009>.

172. Wu W-J, Maru S, Regmi K, Basnett I. Abortion care in Nepal, 15 years after legalization: Gaps in access, equity, and quality. Health and human rights. 2017;19(1):221.

173. Yavangi M, Sohrabi MR, Riazi S. Out of pocket payment for obstetrical complications: a cost analysis study in iran. International journal of preventive medicine. 2013;4(11):1296-303. Epub 2014/01/10. PubMed PMID: 24404365; PubMed Central PMCID: PMCPMC3883255.

174. Zamberlin N, Romero M, Ramos S. Latin American women's experiences with medical abortion in settings where abortion is legally restricted. Reprod Health. 2012;9(1):34. Epub 2012/12/25. doi: 10.1186/1742-4755-9-34. PubMed PMID: 23259660; PubMed Central PMCID: PMCPMC3557184.
