# Supplementary material for: The microeconomics of abortion: A scoping review and analysis of the economic consequences for abortion care-seekers
Source: PLoS One. 2021 Jun 9;16(6):e0252005. doi: 10.1371/journal.pone.0252005 (PMC8189560; doi:10.1371/journal.pone.0252005)
Supplement: S2 Appendix — (DOCX) [file pone.0252005.s002.docx]

## S2 Appendix. Summary of studies reporting microeconomic impacts

| **Author, year [country]** | **Aim/objective(s)** | **Population** | **Study type** | **Summary of main findings** |
| --- | --- | --- | --- | --- |
| [1] [Bangladesh] | To find out where women go for induced abortion in rural Bangladesh today, their contraceptive practice prior to and after getting pregnant, their reasons for choosing abortion, who makes the decision for abortion, what complications they develop and where they go for treatment for these | Women seeking abortion services | Mixed methods using semi-structured questionnaire | Money was obtained by borrowing from relatives and selling jewelry. The woman and her family are now living in extreme poverty |
| [2][Ireland] | (1) to examine the factors affecting whether women in Ireland choose to access abortion by travelling or by using online telemedicine; and (2) to explore their experiences in accessing care through each pathway | Women (n=38) identified through three organisations: Women on Web, Abortion Support Network, For Reproductive Rights Against Oppression, Sexism and Austerity.  Criteria: aged over 18, had an abortion within 8 years of study, lived in Ireland at time of abortion, had travelled or used telemedicine to access abortion care. | Qualitative in-depth interviews | One respondent (aged 35) who took out a loan to afford to travel to receive abortion care in England commented:  “If you’re feeling desperate and your support system is non-existent, you do very desperate things. There was this forum online talking about having a hot bath and drinking a bottle of vodka, or going in there with a long, sharp instrument. I tried the hot bath. And there’s a fitness class that’s on a mini trampoline, and people told me that if you go on that within the first 6 to 8weeks, you miscarry. So, I tried that religiously for 2weeks, but it didn’t help. It was just pure desperation” |
| [3] [France] | To determine whether women undergoing repeat abortions are exposed to risk factors which might be amenable to preventative measures, and the methods employed by carers in these cases | Women who had undergone two abortion up to 1997 (n=30) and interviews with the care team | Survey of women and interviews with the care team | Guilt, depression and psychological distress were found in 21 patients out of 30 after the second abortion. Patients undergoing repeat abortions request psychological help, but the medical manpower available is well below what is required to meet their needs. |
| [4] [India] | To contextualize the decision to abort in terms of local cultural practices, women's employment in newly emerging peri-urban informal sector, and new forms of encoding masculinities in four villages in northern Tamil Nadu where the fertility rate is rapidly declining. | Women aged between 45-65 and 25-45 who live in the study villages that are marked by high incidence of abortion and industrial estates for pharmaceutical companies | Qualitative:  FGDs with women in-depth interviews with key informants | Women's decision to abort is often a process of accommodating the pressures of cultural values and beliefs. Women's decision to abort seems to be linked to a vector of factors like childcare as their exclusive responsibility, marital conflicts, son preference and belief in astrology and local religion. Since women do not have control over these social conditions, their "choice" of abortion is basically to deal with these situations. The logic of family limitation through abortion as promoted by the state population policy might be influencing women's consciousness on abortion. But how women arrive at the decision to abort is not based on the received notions of family planning or birth control, but on the basis of local, social and cultural conditions and relations that define their lives. In the case of young unmarried working women, as the study shows, what they attempt to do by means of abortion is to negotiate the harsh realities of work and the increasing control over their sexuality at the workplace and at home through their sense of reproductive entitlement. While abortion by unmarried girls may signify subtle sexual and reproductive strategies or even a "counter hegemonic morality" to the existing social conditions, it also simultaneously indexes women's lack of control over the oppressive work conditions and social norms. Given this interlocked nature of labor rights and reproductive rights, it is important to seek labor rights such as the right to equal wages, enabling work conditions and right to unionization, as part of seeking reproductive rights. |
| [5] [Ghana] | To explore and understand the reasons why women terminate their pregnancies and their experiences of seeking services in order to know what and how to reform services to reduce unsafe abortion. | Women admitted to the hospitals with incomplete abortions (n=131) | Semi-structured hospital-based survey | When asked what their partners’ reaction to the pregnancy was, more than half of respondents said their partners did not like the pregnancy. The 67 women who said their partners were not happy with the pregnancy were asked whether they knew the reasons for their partners’ reaction. Almost half of these respondents said their partners feared the reaction of the girls’ parents and other socio-cultural problems. One-third mentioned financial constraints, 12% said it was to avoid disrupting the girls’ educational or career opportunities and 6% said their partners doubted their paternity. The 53 respondents who reported their partners were happy with the pregnancy were asked the reasons why they terminated their pregnancy despite this. Fifteen percent said they aborted because they were not yet ready to have a child or they were nursing another child. Other reasons given were similar to those cited for the partners not wanting the pregnancy: financial constraints, academic/career pursuits, and a range of socio-cultural issues like not being married to the man responsible for the pregnancy, not ready to be second wives, unstable marriages and having different religious inclinations from partners. The great majority of respondents were young and single presenting a typical pattern, found in other studies, of younger women wanting to delay childbearing until they are married or have furthered their education. |
| [6][Ghana] | To explore the factors that are likely to influence abortion decisions among University students in Ghana and their knowledge and perceptions on abortion. | Randomly sampled students of the University of Ghana (n=142) | Qualitative: FGDs | Evidence from this study suggests that the main factor considered by students in their decision to abort is the likely effect of the pregnancy and the birth of the child on their education. Pregnant students, together with their partners or parents, weigh the prospects and the investments and sacrifices that have been made in their education with the impending termination or delay in education as a result of the pregnancy. Specifically, students consider the length of time left to complete school, their desire or prospects for further education, and the adverse effect of the pregnancy and the newborn child on their studies in their decisions to abort. Consistent with the findings of Hubbard, the present study showed that students with good academic grades are more likely to abort to stay in school than poor performing students who had little academic prospects. Students with scholarships or under sponsorships, as well as those with funding difficulties, are also more likely to abort to avoid the effects the pregnancy may have on the length and completion of their education and their funding opportunities. Additionally, students who have other options of continuing their course, including opportunities to convert to part-time, off-campus distance students, or even deferring the course for the duration of the pregnancy, are also less likely to have an abortion because of their education. |
| [7] [United States] | To conduct a systematic review of TRAP (Targeted Regulation of Abortion) laws and their impact on abortion trends and women’s health | Women exposed to TRAP policy | Systematic review | TRAP laws may not need to close clinics to have an impact on health and other outcomes: some laws may instead increase service costs or decrease availability of appointment slots, both of which could increase the time it takes for a woman to obtain an abortion. An increase in gestational age at presentation may limit the number of providers willing to perform an abortion (particularly if the pregnancy has entered the second trimester) and increase out-of-pocket costs to patients. While women with adequate resources are generally able to obtain an abortion with minimal difficulty, regardless of local policies, access-oriented barriers to abortion may introduce special challenges to low-income, young and/ or rural women, as these women may be less able to manage increases in cost and distance. |
| [8][Sweden] | To explore what women who have had a pregnancy termination due to a detected fetal malformation perceive as being important in their encounters with caregivers | Pregnant women with fetal malformation attended four clinics specializing in ultrasound in the Stockholm area of Sweden | Semi-structured interviews | A respondent describes the impact that cost had on seeking psychological support:  “I wanted a more professional psychological help and phoned a therapist, but the high cost, 1200 Swedish crowns per hour was not feasible in the circumstances of me sick-listed and [having] no insurance coverage because a fetus does not count as a legal person before gestational week 22” |
| [9] [Brazil] | To determine social and behavioral consequences of pregnancy and how these differed according to the pregnancy outcome (live birth or abortion) 1 year after the event. | A cohort of pregnant teens who sought  prenatal care at the Adolescent Clinic (n=367), and a cohort  of girls of the same age who attended the same  hospital but who were admitted through the emergency  services with complications from abortion (n=196) | Cohort analytic | Induced abortion patients were the most likely to be enrolled in school (68%). About two-thirds of the girls whose pregnancies were intended and those who miscarried had already left school before the first interview. The primary reason for leaving school as stated by all groups, except the girls with induced abortions who quit school to work, was their pregnancy or their marriage (39% among the adolescents with intended and unintended pregnancies). Others stated that they simply did not like school (25% overall). Induced abortion patients were the most likely to be working (33%)." Employment status did not change for any group but school enrollment dropped from more than half of the teens enrolled at baseline to only 31% at 1 year. The vast majority of these girls dropped out; a few may have finished the equivalent of high school. The largest percentage of school attendees at 1 year was among the induced abortion patients. Compared with adolescents whose pregnancies were intended (the reference group), the probability that girls with induced abortions would be in school at one year was 6.9 times as great, girls with spontaneous abortions were five times as likely to be enrolled, and adolescents whose pregnancies were unintended were three times as likely to be in school. Age was inversely associated with school enrollment (the odds of being in school decreased as age increased). Adolescents enrolled in school at baseline increased their odds of staying in school almost six-fold. However, the likelihood of being in school decreased by half if the adolescent had a sister who also had experienced a pregnancy during adolescence. |
| [10] [United States] | To explore qualitatively the experiences of women who were most affected by restrict abortion laws in Texas: those who had to travel farther to reach a facility and those desiring medication abortion | Women recruited from ten abortion clinics across Texas. The purposive sample included women who obtained or strongly preferred medication abortion or traveled ≥50 miles one way to the clinic. (n=20) | Qualitative, in-depth interviews | Women who completed the in-depth interviews not only spoke about the types of costs associated with attending multiple visits, including lost wages for themselves and their support network, and paying for child care, gas or public transportation, but also how poverty often compounded the barriers they faced. This led some to consider not having the abortion, and one woman ultimately decided to continue her pregnancy at least in part because of the obstacles she encountered. |
| [11] [Benin and Burkina Faso] | To document the means women use to obtain abortions in the capital cities of Benin and Burkina Faso  To learn whether or not use of misoprostol has become an alternative to other methods of abortion and the implications for future practice | Women in Cotonou (n=21) and in Ouagadougou (n=13), including 5 secondary school students | Qualitative in-depth interviews | The requirement of a prescription to access misoprostol in Benin often leads to purchasing through informal sellers such as the Adjegounlè drugs market in Dantokpá international market. |
| Berer 2000 [Global] | To examine the changes in policy and health service provision required to make abortions safe | Literature review with multiple populations | Literature review | In Bangladesh, women tend to wait until complications become severe before seeking help, increasing both the cost and complexity of treatment. Furthermore, women attending untrained providers have been found to make more visits for care and spend more overall than women attending trained providers in the first place. |
| [12] [United States] | To learn about women’s experiences applying for subsidized insurance and to identify barriers to obtaining insurance or its use for abortion services | English-speaking women who met the eligibility requirements for subsidized insurance programs in Massachusetts: Women whose household income is less than 300% FPL, who are uninsured, who are American citizens or have been permanent residents for more than 5 years, and who are Massachusetts residents (n=39) | Systematic, qualitative interviews with women | A 19-year-old who first contacted the funds at 23 weeks’ gestation, described “timing out” of an in-state surgical abortion when she was unable to become enrolled right away; nor would MassHealth cover abortion care provided by out-of-state providers. She ultimately completed her pregnancy, intending to give the baby up for adoption, but explained that she was very disappointed by MassHealth. Delays also limited women’s ability to obtain medication abortion. As medication abortion is typically provided through the 63rd day after the woman’s last menstrual period, several women reported considerable anxiety that they might not obtain insurance in time. Although at least one woman was able to obtain and use Commonwealth Care for a medication abortion in a timely way, the approaching deadline prompted abortion funds to provide grants to at least two desperate women. Both women were later deemed eligible for MassHealth, suggesting that the costs for abortion should have been covered by insurance. A third woman, aged 18, reported being so fearful of “surgical abortion” that she ultimately decided to continue her pregnancy after her struggles with MassHealth put her past the limit for medication abortion. She first applied for insurance at 6 weeks’ gestation; by the time of follow-up 37 days later, she was still unsure if she had been approved for MassHealth. The delays caused by attempts to enroll in subsidized insurance had a disproportionate impact on women who sought medication abortion, forcing them to pay out of pocket or placing their preferred method for termination out of reach. |
| [13] [United States] | To assess women's interest in and support for three alternative models of MA provision: (1) advance provision with a prescription from a clinician in case of future need, and nonprescription (2) OTC access from a pharmacy and (3) online access, using a representative sample of United States women | A national, probability-based representative sample of United States women ages 18–49 (n=7022) | Online survey | Perceived advantages of alternative forms of medication abortion provision reported for advance provision, over the counter and online access included: could be less expensive (35% advance provision, 34% OTC access, 32% online access). Perceived disadvantages included: could be more expensive (16% advance provision, 20% OTC access, 18% online access). |
| [14] [Latin America and Caribbean] | To review results from 10 major PAC operations research projects conducted in public sector hospitals in seven Latin American countries, completed and published 1991-2002 | Review of operations research, that is, studies designed to measure the effectiveness of an intervention in achieving service delivery outcomes | Review paper | Long waits before and after treatment are often a major source of PAC patient dissatisfaction. Switching to MVA and reorganizing services can contribute to shorter average lengths of stay for clients. |
| [15] [Ireland] | To consider abortion tourism in Ireland, both north and south, and how the moral conservatism present in both jurisdictions has impacted on attitudes and access to abortion | Women seeking abortion serves in restricted setting, i.e. Ireland | Mixed methods | The moral conservatism in the Republic of Ireland and Northern Ireland is apparent in the role of religious and political institutions. Both fail to acknowledge the repeated evidence of the public and professional support for abortion law reform and the evidence of the continuance of abortion tourism. Their comments on abortion demonstrate that they fail to grasp the complex set of circumstances women find themselves in when faced with a crisis pregnancy. In denying women their agency, these comments reinforce perceived traditional feminine roles as fertile, caring and inevitable mothers, in effect forcing them into ‘reproductive labor.’ |
| [16][Colombia] | To identify the key barriers to legal abortion, and to explore the ways they may work separately and together to delay the receipt of high quality, legal abortion care | Women who had obtained a legal abortion in Bogotá, Colombia (n=17). Eligible for inclusion if aged 18 or older, had obtained an abortion in the past 12 months and exhibited verbal proficiency in Spanish | Qualitative: In-depth interviews with women | Women experienced other logistic barriers. Fifteen of the 17 women had to take time off from work or school to obtain their abortion, and nine had to arrange for child care, all of which delayed the abortion. |
| [17] [United States] | To estimate directly the responsiveness of abortion demand to county-level variations in travel-cost component of the full cost of abortion services | Abortion providers in Texas | Log-linear regressions using data that were obtained from health facilities on each abortion performed and data on the localities of these facilities | Through their modelling they estimated that raising the cost of travel for abortion care by 1 USD would decrease abortions per number of women by 1.31 percent and abortions per pregnancy by 0.97 percent. |
| [18] [United States] | To examine how partners figure in women’s abortion decisions, and identifies factors associated with identifying partner as a reason (PAR) for abortion | Women recruited from 30 U.S. abortion facilities where no facility nearby offered care at a later gestational age of pregnancy. Study eligibility included being pregnant, English or Spanish speaking, 15 years or older, not having any known fetal anomalies or demise, and belonging to one of three groups: 1) Women just over the facility’s gestational age limit and denied an abortion (n=231), 2) women just under the limit and who received an abortion (n= 452), and 3) women receiving a first trimester procedure (n = 273) | Mixed methods using prospective longitudinal survey and in-depth interviews | One man involved as a partner in a pregnancy report wanting to go back and finish school to make enough money to raise a child. |
| [19] [Thailand] | To obtain data regarding: pregnancy history; number of abortions and cost of abortion related treatments; abortion complications, impacts and related health care services; reasons for having an unsafe abortion; and, circumstances related to an unsafe abortion from women who had undergone a recent abortion (spontaneous, therapeutic and unsafe) | Thai females, of any age, who: were able to speak, write and understand Thai; had experienced an abortion, regardless of chronological age, gestational age or type; and admitted to one of the study site hospitals. | Structured self-report questionnaire | Being a student in an educational program was one of the most frequently stated social reasons for undergoing an unsafe abortion. |
| [20] [United States] | To explore abortion patients’ perspectives on state regulations, including mandating waiting periods and the provision of state-authored information, and prohibiting private and public insurance coverage for abortion | Abortion patients | Qualitative: Semi-structured interviews | The reasons for choosing abortion among the women in our sample were diverse, but for most they matched the general categories identified in recent research that having a child would interfere with a woman’s education, work or ability to care for dependents. |
| [21] [South Africa] | To report whether a significant proportion of women seeking abortion in public sector services would be early enough in pregnancy to be eligible for medical abortion and to investigate the hypothetical acceptability of medical abortion among women, policymakers and providers and whether women would attend for follow-up. | People (n=673) attending abortion services in 8 facilities across three provinces in South Africa | Cross sectional survey | Financial concerns were the second most cited reason among rural women for delaying seeking abortion related care. 20% of women cited financial reasons. |
| [22] [United States] | To document the effects of abortion-clinic closures on clinic access, abortions, and births using variation generated by a law that shuttered nearly half of Texas' clinics | Women of reproductive age and children | Regression analysis | The distance the average Texas woman had to travel to reach an abortion clinic increased from 21 miles in the quarter prior to House Bill 2 to 44 miles in the quarter immediately after. Women who had to travel more than 100 miles (one-way) to reach a clinic increased from 5% to 15%, with the largest increases in travel distances occurring in the central-western region of Texas. Travel distance to the nearest clinic was unchanged for women whose nearest abortion clinic was already located in a major city because at least one clinic remained open in these cities. |
| [23][Romania] | To explore the psychosocial antecedents and consequences of the Romanian pronatalist policies banning importation of contraception, prohibiting abortion, and imposing tax on childless couples | Women, aged between 18 and 55 years in 1998, the year before the December 1989 revolution (n=50) | Qualitative: in-depth interviews | While the reality of the unwanted pregnancy was usually a stressful moment for most women, the decision to abort was made relatively quickly, seldom involving ethical concerns. Many women indicated that their motivation was determined by socioeconomic conditions that would not allow them to have additional children within their standard of living. The most frequent socio- economic reasons mentioned were the lack of adequate housing and the chronic shortage of food. Four women said that a child would interfere with their studies or their profession; another four said that they did not want to be a single parent. There was fear that the pregnancy would be discovered, especially in cases of women working in a factory where compulsory gynecological examinations took place at least every 3 months. |
| [24] [United States] | To answer: 1) What do women know about the cost of abortion and the availability of Medicaid coverage for abortion? 2) Where do women obtain this information? and 3) What are women’s experiences paying for care? | Women seeking abortion care in study states: 1) was age 18 or older, 2) had an abortion within the past two years, 3) resided in one of the four study states at the time of the abortion, and 4) was low- income, which we defined as meeting the Medicaid income qualifications of the state where she had the abortion (n=98) | Qualitative: in-depth interviews with low income women who had abortions | Policies regarding Medicaid coverage of abortion affect the lives of women and their families in numerous ways. Restrictive coverage policies appear to force women to take measures to raise money for an abortion that may put their health and wellbeing at risk, promote short and longer-term financial instability, and increase the difficulty of implementing an abortion decision, thereby interfering with women’s reproductive life plans. Restrictive Medicaid coverage policies also appear to have ripple effects on children, partners, and parents of women seeking abortion services. We believe that a nationally representative study is needed to test our emergent hypothesis that low-income abortion clients in states without Medicaid coverage of abortion experience significantly more emotional and financial harm than clients in states where coverage is available. More research is needed to quantify the extent of emotional and financial duress placed on women and their families, and how this duress affects individuals and families over time. |
| Diniz 2012 [Brazil] | To address equity in health and health care in Brazil, examining unjust disparities between women and men, and between women from different social strata, with a focus on services for contraception, abortion and pregnancy | Review paper from Brazil | Review paper | Safe abortions are only provided in the private sector illegally; they are prohibitively expensive for poorer women but affordable for those with a higher income. Women with lower income tend to use more affordable methods, such as misoprostol (again, illegally, from the black market), turning to public services for treatment when there are problems. In 2008, there were 215,000 hospitalizations in SUS for complications of abortion (or miscarriage), especially from hemorrhage and infection. |
| [25] [United States] | To identify factors, from a systematic literature review, that facilitate and hinder access to abortion services for women in developed countries in relation to first-trimester abortions, from the perspective of both the woman and the service provider | Multiple populations within systematic review | Systematic review | 85% of women said that they would be willing to pay for an earlier abortion |
| [26] [United States] | To synthesize published literature and current practices for adolescent abortion care | Adolescents aged 19 years and younger | Descriptive analysis of current evidence and practice | Adolescents faced with an unintended pregnancy give several reasons for their decision to have an abortion; these reasons are similar across diverse groups of young women. Adolescents feel unprepared for motherhood both psychologically and financially and are concerned that childbearing will adversely affect educational attainment, career opportunities, and personal relationships with their partners and family members. Observational research supports these concerns by demonstrating better educational outcomes among adolescents choosing abortion compared with those continuing pregnancy. One study found that those undergoing abortion were more likely to complete high school than those who carried their pregnancies to term. Another 25-year longitudinal study found that those adolescents undergoing abortion achieved higher levels of education than those carrying to term, even after adjustment for family, social, and baseline educational differences. The availability of affordable abortion services determines if a young woman can obtain an abortion at all and how quickly. Compared with adults, barriers to care may particularly affect adolescents with limited resources. Obtaining an abortion may require significant travel because most counties in the United States (more than 80%) have no abortion provider. In most states, abortion is not covered by state Medicaid programs and poor teens may have difficulty funding the cost of care. Delays experienced by adolescents lead to increased costs and risk, as later abortions are more technically difficult and expensive. |
| [27][United States] | To explore abortion-related hardships in a previously understudied group: patients in the United States who received ﬁnancial pledges from the National Network of Abortion Funds’ (NNAF) Tiller Memorial Fund, to pay for an unaffordable abortion | National sample of US patients who received a ﬁnancial assistance pledge to help cover abortion procedural costs (n=3,999) | Descriptive, exploratory, cross- sectional analysis of administrative health care data of patients | A total of 19 categories of hardship emerged. On average, persons in the sample experienced 2.29 hardships (SD 1.13), with a range from 0 to 7 hardships. Out of the total sample of 3,999 cases, 82.8% (N 3,311) experienced at least one hardship. The most frequently occurring hardships involved having multiple children, deﬁned as two or more (N 1,511; 37.8%); currently being on some form of public assistance (e.g. food assistance) (N 1,482; 37.1%); traveling distances greater than 50 miles to obtain an abortion procedure (N 1,151; 28.8%); not being on birth control (N 967; 24.2%); or having birth control failure (N 893; 22.3%); currently unemployed and seeking employment (N 785; 19.6%); and having unstable or partial housing (N 747; 18.7%). These findings are consistent with other studies, and the authors make linkages to the broader economic hardships faced by this population. Some of the less-researched hardships identiﬁed in this study are problems with employment/current unemployment, marginal housing or homelessness, and medical problems. These results are, however, similar with ﬁndings suggesting that unemployment, partner unemployment, problems paying for housing and problems paying for life necessities were common in abortion patients and people with a recent history of abortion. A modest proportion of the patients from the sample also experienced a variety of medical conditions, including obesity, and preexisting mental health and substance use diagnoses, which is consistent with studies suggesting a link between unplanned pregnancy and obesity, existing mental health problems, and substance use, in adults and adolescents. |
| [28][United States, Republic of Ireland, Northern Ireland, Isle of Man] | To examine the experiences of abortion fund patients in the USA and Republic of Ireland, Northern Ireland and Isle of Man to compare abortion fund patient experiences across these developed nations for the first time | Abortion fund cases for Republic of Ireland, Northern Ireland, and Isle of Man combined 6340 cases (n=3995 from the USA and 2345 from the RI, NI and IM) | Cross-sectional descriptive analysis | There is evidence that factors related to patients’ access to abortion are similar across these countries, which include similarities in the number of children that patients have. These findings demonstrate the commonality of the parenting conundrum faced by economically vulnerable patients who are trying to gather the resources to access abortion across all of these nations. These patients are typically already parents, which suggests that they are already devoting resources to rearing existing children, which is consistent with other research from Biggs, Gould and Foster (2013) indicating that patients seek abortion so they can focus on other children. Additional results suggest that fund recipients are faced with a dearth of available resources, despite the help offered by the funds. Even with abortion fund assistance, these patients had notable resource deficits when trying to access abortion services. |
| [29][Iran] | To provide direct estimates of abortion levels in Tehran for both all women and selected subgroups: estimate the abortion rate and the proportion of known pregnancies that end in abortion for the population of married women in Tehran, to examine variations by women's demographic and socioeconomic characteristics, and to assess reported reasons for having an abortion and the contraceptive method used at the time of conception | Women seeking abortion in Tehran, married women aged 15-49 (n=2,934) | Cross-sectional survey | Twenty-one percent of abortions performed in the five years preceding the survey were undertaken for a socioeconomic reasons: 19% for economic difficulty and 3% for a spousal relationship problem (divorce, separation or a partner's drug addiction.) Fifteen percent of respondents who knew another woman who had had an abortion in the previous year said the main reason for them undergoing the procedure was a socioeconomic issue. |
| [30] [Ghana] | To explore the pre and post experiences of young people (aged 12 to 24) who had their abortion three months prior to the study | Young people (aged 12 to 24) who had their abortion three months prior to the study | Qualitative: pre/post in-depth interviews | Reasons given for young females’ decision to abort abortion are consistent with literature. Principally, inability to care for the babies owing to household poverty, fear of dropping out of school, societal/community stigma and shame were mentioned. |
| [31] [Spain] | To describe the determinants of the IVE [voluntary pregnancy interruption] delay until the second trimester of pregnancy in the city of Barcelona, between 2004 and 2005 | Women who reside in the city of Barcelona who obtained abortions for physical or mental health issues between 2004 and 2005 (n=9.175) | Cross-sectional study | Women with an unfavorable socioeconomic position take more time in learning that they are pregnant. In Catalunya, the pregnancy test is not covered by the National Health System (SNS) and its price is between 10 and 20 euros. In Spain, there is no uniform criteria that determines what kind of IVE is financed by the SNS, and in practice, public centers conduct some of IVEs that result from fetal malformation, while those conducted because of women's health are conducted in private centers. |
| [32] [United States] | To analyze data on women who sought and received an abortion at or after 20 weeks’ gestation for reasons other than fetal anomaly or life endangerment | People who were seeking abortions after 20 weeks gestation across 16 sites (n=272) and people who presented for first trimester abortions (n=169) | Mixed methods – qualitative data from interviews and quantitative data for logistic regression | Gives the case study of Amber [a pseudonym] whose gestational age meant that she had to seek 600 USD from an abortion fund, 600 USD from her ex-boyfriend and 300 USD of her own money to obtain abortion related care. This occurred after contacting three clinics to find a provider of care. |
| [33][Canada] | To document women's experiences obtaining abortion care in New Brunswick before and after the Regulation 84-20 amendment (Regulation 84-20 has historically restricted funded abortion care to procedures deemed medically necessary by two physicians and performed in a hospital by an obstetrician-gynecologist); identify the economic and personal costs associated with obtaining abortion care; and examine the ways in which geography, age and language-minority status condition access to care | New Brunswick residents who obtained abortions (n=33) | Qualitative: semi-structured in-depth interviews | One respondent decided to have an abortion because she was not in a financial position to care for another child. |
| [34] [United States] | To describe women’s experiences seeking abortion care shortly after clinics closed and document pregnancy outcomes of women affected by these closures | Women who sought abortion care at Texas clinics that were no longer providing services and Texas resident women seeking abortion in Albuquerque, New Mexico. (n=23) | Qualitative: in-depth interviews | Most women, especially those in West Texas and the Lower Rio Grande Valley (LRGV), spent more money and time than they would have before HB2 to obtain an abortion after their local clinics closed. Women also described being uncomfortable, lonely and feeling sick while traveling far from home. Women also reported that they needed to ask for more help than they would have if there had been a local provider, and in the process, they had to reveal their abortion decision to people they might not have told otherwise. Some discussed needing to borrow a vehicle or ask someone to drive them to their appointments, and others mentioned that they had to borrow money from friends to help pay for the abortion and travel costs. A few women compromised their desire for privacy in order to make arrangements to obtain an abortion. After being turned away from closed clinics or having appointments canceled most women who had to arrange transportation or could not take time off of work on the day of the next available appointment were delayed even further. Two women who wanted an abortion did not obtain one despite attempting to schedule an appointment. Insufficient information, time and money led these women to the decision to carry their pregnancies to term. |
| [35] [Vietnam] | To understand the determinants of delaying obtaining abortion until the second trimester | Clients presenting for an abortion at 13–24 weeks of gestation (n=60) | Qualitative: semi-structured interviews | Respondents described factors that delayed their accessing or receiving adequate abortion services. Four women described difﬁculties in obtaining leave from an employer or taking time off from agricultural labor that had prevented them from obtaining health care earlier. Women might have hesitated in making a decision because of a conﬂict between wanting to continue the pregnancy and their economic or educational situations. About half of the women identiﬁed inadequate resources as the primary factor for ending their pregnancy. Fifteen women speciﬁcally described negative consequences to their educational or employment opportunities from having a child at the present time. Other respondents described constraints on employment opportunities from childbearing. Women might have been initially ambivalent about the pregnancy as a result of discordant demands from cultural mores, economic realities, or educational aspirations, and this ambivalence could have delayed their decision to seek an abortion. |
| [36] [Zambia] | To examine sales practices, knowledge, and behavior of pharmacy workers regarding medical abortion in 2009 and 2011 in Zambia, where hostile and stigmatizing attitudes still result in high rates of unsafe abortion. | Pharmacy workers at government-certified pharmacies in the intervention areas (76 pharmacies in November 2009 and 80 in November 2011) | Descriptive cross-sectional design | In 2009, 35 (46%) clients left pharmacies with more information or with the opportunity to purchase medical abortion drugs; however, in 2011, this proportion was higher with 53 (66%) mystery clients reporting this (P = 0.0110). |
| [37] [India] | To study the impact of prenatal sex selection on the well‐being of girls by analyzing  changes in children’s nutritional status and mortality during the years since the diffusion of sex selective  abortion in India. | Households in India | Econometric analysis of national survey data | An increase in the practice of sex‐selective abortion appears to be associated with a reduction in the incidence of malnutrition among surviving girls. This negative association is stronger for girls born in rural households and at higher birth parities. We find no evidence that sex‐selective abortion leads to selection of girls into families of higher SES. We do find some evidence of a larger reduction in family size for girls than for boys and we also find some suggestive evidence of better treatment of girls as reflected in breastfeeding duration. On the other hand, sex‐selective abortions do not appear to be associated with a reduction in excess female child mortality. We estimate the model using our main sample (the last two children born within 3 years prior to each survey round) as well as for samples stratified by rural/urban residence and parity (i.e., the same samples used for the nutritional analysis). There is a disadvantage for girls in breastfeeding duration that widens with age. For example, girls are one percentage point less likely than boys to be breastfed for at least 12 months, about 4 percentage points less likely to be breastfed for at least 18 months and almost 6 percentage points less likely to be breastfed for at least 24 months. Girls’ disadvantage in breastfeeding duration is larger in rural areas and in parities higher than one. |
| [38] [Burkina Faso] | To study both costs and consequences of induced and spontaneous abortions and complications | Women whose pregnancy ended with either an induced or a spontaneous abortion (n=305) | Cross-sectional study that collected cost data from the patient perspective | The average expenditure associated with abortion represented 15% of the GDP per capita for women with induced abortion and 9% of the GDP per capita for those with spontaneous abortion. Additionally, 12% percent of the sample of women incurred subsequent economic consequences, as expressed by their need to resort to measures such as reducing expenses on essential needs or using their entire savings, etc., to pay for their hospitalization. Compared with women with spontaneous abortion, women who had had an induced abortion seemed to have faced unaffordable abortions costs with a higher proportion of these women bearing the economic consequences associated with catastrophic health care payments (16% vs 11%). Moreover, the statistical difference between women with induced abortion and those with spontaneous abortion as to unaffordability of costs associated with abortions seems to suggest that access to abortion and/or post-abortion care may have impoverished much more women with induced abortion compared with women with spontaneous abortion. |
| [39] [United States] | To examine the breadth of barriers, beyond those related to individual state-level abortion restrictions, that such women encounter and any associated consequences | Patients seeking abortion services  (n=29) | Qualitative / in-depth interviews | Participants described 15 barriers they encountered while traveling to obtain care. We grouped like barriers into five groups: travel-related logistical issues, system navigation issues, limited clinic options, financial issues, and state or clinic restrictions. Barriers to obtaining abortion reported by women who traveled to receive services, and number of women (n=29) reporting each, by barrier group: Making arrangements after appointment was scheduled (e.g., for transportation, accommodations, child care and work schedule changes) = 27; Financial issues (including below sub-groups) = 25; Need to raise money for procedure and related costs (e.g., travel, logistics) = 20; Lack of insurance coverage = 13 ; Difference in procedure costs between clinics = 8 |
| [40] [United States] | To estimate the responsiveness of teenage abortion rates to variations in the local availability of abortion providers using a model of fertility control. | Teenage girls in Texas | Analysis of abortion survey data analyzed through logistic regression | The coefficients on poverty are statistically significant and negative, implying that higher poverty rates are associated with  lower teenage abortion rates. The poverty measure could be correlated with eligibility for income assistance programs, and teenagers who are eligible for income assistance have  lower opportunity costs of giving birth. Counties with a higher proportion of high-school educated residents also have higher teenage abortion rates, reflecting higher opportunity costs associated with having a child while enrolled in high school. Female employment conditions have no statistically significant effect on abortions per woman, but counties with higher percentages of females employed have lower abortion rates per pregnancy. This result is unexpected, because better employment conditions raise the opportunity cost of children and, therefore, should increase rates of abortions. Counties with higher proportions of married households have lower abortion rates per woman but higher abortion rates per pregnancy. The difference in coefficient signs could indicate that teenagers from married households are less likely to become pregnant, although they are more likely to abort if a pregnancy occurs. The religious affiliation measures are statistically insignificant, with the exception that counties with higher proportions of Catholics have lower rates of abortion per pregnancy. More urbanized counties and those with higher percentages of white residents are shown to have lower abortion rates. The coefficient sign on family planning clinics indicates that counties with relatively more clinics tend to have higher abortion rates; however, this coefficient is only statistically significant for abortions per woman. This result may reflect a supply response to household preferences for children: family planning clinics tend to locate where there are higher pregnancy rates |
| [41] [United States] | Unclear objective: To address a set of laws targeting abortion providers, which have a significant capacity to reduce access to and quality of abortion care in the United States | Legal review for whole United States | Review paper | The second type of regulation unnecessarily requires abortions to be performed in ASC facilities set up for more sophisticated and intrusive surgical procedures. These costly requirements may force many providers to stop offering services or to raise their prices to levels prohibitive for some women seeking care. The costs and burdens stemming from the imposition of ASC requirements have hindered or prevented physicians in some states from providing abortions. For pregnant women, the corresponding effect of the laws and physicians’ response to them has been to hinder (and possibly preclude) timely access to safe and legal abortion services. |
| [42][United States] | To examine the characteristics of women having abortions at 13 weeks or later | A national sample of 9493 women obtaining abortions in 2008 | Regression analyses. Data analyses of the 2008 Abortion Patient Survey | 1/3 of people relied on health insurance to pay for their abortions.  Women using health insurance were twice as likely to have an abortion at 16+ weeks than those paying out of pocket and women in poverty were more likely to have later abortions than women who are poor. |
| [43] [United States] | To determine which characteristics and circumstances were associated with obtaining very early and second-trimester abortions | 8380 non-hospital abortion patients from the 2014 Abortion Patient Survey | Regression analyses | 45% of patients paid out of pocket for care, and Medicaid was the second most common method of payment.  14% of women used private insurance, 13% used financial assistance, which refers to discounts provided by clinics or subsidies available at some facilities. |
| [44] [United States] | To determine which characteristics are associated with prior abortion | 8380 non-hospital abortion patients from the 2014 Abortion Patient Survey | Regression analyses | The odds of having a prior abortion were higher for those who paid for the procedure using public or private health insurance (OR: 1.47; 95% CI: 1.29– 1.69) / received financial assistance (OR: 1.32; 95% CI: 1.15–1.52), compared to patients who paid for the abortion out of pocket. |
| [45] [United States] | To (1) describe risk factors for obtaining a second trimester abortion and (2) to further elucidate associations between several risk factors and delayed requests for abortion among women presenting to a high-volume family planning clinic in the Midwest | English-speaking women aged 18 years and old presenting for surgical abortion for all indications except fetal malformation, in a Chicago based clinic. | A patient survey with regression analyses | Of women aged 18-21, 64.4% had to pay out-of-pocket for their procedures. |
| [46][Egypt] | To examine the economics of abortion safety in Egypt. | Egyptian  women who sought to terminate their pregnancies (n=18) | Qualitative | Abortions categorized as being the “most safe” in the study were primarily available to upper class women, who had biomedical abortions performed by their own private gynecologists. Women in the study overwhelmingly preferred biomedical abortions, which they considered ``cleaner.” Almost universally the reason that women gave for using indigenous methods was that they could not afford biomedical abortions. |
| [47] [India] | To examine the complex interplay between reproductive experiences and women’s empowerment | Women in Madhya Pradesh, India (n=2,435) | Logistic regression using household based probability survey | In the model with multiple reproductive events, the association between abortion successes and violence remains statistically significant after controlling for initial empowerment conditions, it loses its significance in the model including only abortion. This is probably attributable to the significant negative relationship between mistimed pregnancies and violence. This underscores the importance of examining multiple reproductive events simultaneously to better reflect the influences on women’s empowerment. |
|  |  |  |  |  |
|  |  |  |  |  |
| [48] [Zambia] | To estimate the costs for women of seeking safe and unsafe abortion and to establish whether the burden of abortion care-seeking costs is equally distributed across the sample | Women receiving care for either safe abortion or post-abortion care at University Teaching Hospital, Lusaka | Cross-sectional survey | We considered women and their household’s financial (e.g. having to find someone to cover work, or selling assets) coping mechanism in response to care seeking. However few women discussed these, possibly because the average duration of the stay was 3.5 h for SA vs 4.5 for PAC and if they stayed overnight the average number of nights was 0.77 (0.68 for SA vs 0.88 for PAC) which meant they could get on with their life more easily (e.g. less need to look for extra childcare). On average, women missed around 2.2 days of either school or work (2 for SA vs 4.8 for PAC) which includes time to get to and from UTH and time spent at UTH as well as time spent ill due to an unsafe abortion. |
| [49][South Africa] | To explore women's experiences accessing services and estimate costs incurred for first-trimester abortion at four public hospitals in KwaZulu-Natal Province, South Africa | Women seeking abortion care: women were eligible if they were 18–49 years old, reporting 12 weeks' gestation or less based on their last menstrual period, and presenting at the facility to request an abortion (n=1,167) | Observational, cohort design and aimed to assess and compare clinical and acceptability outcomes | Women's reasons for choosing to have an abortion were varied. Over half (58.9%) said that they could not afford to have a(nother) child because either they or their partner were “not working.” Almost a quarter of the women (23.7%) said that they could not manage a child while studying, and many cited not being “ready” for or not wanting (more) children (43.4%). Public assistance for child support is available but is widely seen as insufficient.  Ninety-one women (12.2%) who had a follow-up visit reported having missed work as a result of their abortion, and 5.4% of all women indicated that they had lost income as a result of obtaining the procedure. The median travel time per visit (round trip) for all women was 50 min [IQR 30–80]. Almost all women (97.2%) reported having to pay for transportation, and 59.6% reported paying for a pregnancy test. Among women who had a follow-up visit (n=780), 95.3% reported having to buy sanitary pads or pain medication. A few women (2.7%) also reported that they had paid for additional items such as food/drink, other doctor's visits and other supplies. Women who reported that their primary source of income was employment, or a grant were also more likely to present early. In contrast, women coming from households that never experienced food insecurity were less likely to present early for an abortion. Interestingly, having a long travel time to the facility or having to pay out-of-pocket for a pregnancy test was not significantly associated with presenting late. |
| [50] [Mozambique] | To compare socio-economic characteristics of women with illegally induced abortion (IA), legally induced abortion  (LA), and women attending antenatal care (AC) by use of a case referent design | IA women (n = 103) were recruited at the emergency ward at the department  of gynecology, Maputo Central Hospital, LA women (n = 103) at the out-patient  gynecology ward of the same hospital and AC women (n = 100) at a peripheral antenatal care clinic | Qualitative interviews and cost data | The profile of the IA and the LA patients, respectively, differed in various aspects. The IA patients were significantly younger and less likely to be in a stable relationship. The IA patients were also at a disadvantage as far as schooling, habitation and household size are concerned, and had employment with low wages. |
| [51] [United States] | To examine the impact of state policies along with other barriers when they seek abortion care | Women in South Carolina | Qualitative | The greatest logistical barriers occurred as women prepared for their abortion appointments. They described the financial burden of paying for the abortion, arranging transportation and negotiating time off work for the appointment and aftercare. Though the interviewer asked participants about child-care arrangements, they did not consider this aspect of preparation to be a major challenge. The majority of women reported difficulty paying out of pocket for the abortion. Many described making adjustments to assemble the funds, including putting off other payments, such as a car loan; refraining from “extras,” such as meals in restaurants; and dipping into a “Christmas gift fund.” Multiple women said the abortion expense was a temporary setback or onetime expense, in contrast to the long-term financial commitment of raising a child. For some women, the fee required their whole paycheck, meaning they would be “short” or “tight” on money for a few weeks. Others said they could afford to make the payment, but “just barely.” Several participants noted that they had had less money in general lately because of lost wages resulting from pregnancy-related symptoms. Some women borrowed from or were given money by friends, family or the man involved in the pregnancy. |
| [52][Norway] | To estimate the benefits of increasing abortion access to teenagers in Oslo | Women in Oslo | Analysis of national data using a difference in difference approach and others | There is compelling evidence that the expansion of abortion access in Oslo for cohorts born after 1950 led to a relative reduction in teenage fertility rates. Access to abortion as a teenager reduces the likelihood of becoming a teenage mother. The coefficient of −0.03 implies that abortion access reduces the probability by 3 percentage points. This is a substantial effect, given that the baseline teenage fertility rate is about 18%. Abortion access also causes these women to postpone when they have their first child by almost a half year and reduces the total number of children before age 20 by about 0.03. Interestingly, there is no evidence of a negative effect of teenage abortion access on completed fertility; if anything completed family size is slightly higher among women who had abortion access. These findings imply that abortion access causes women to postpone fertility rather than reduce it. This is consistent with the fact that the abortion access is at a sufficiently young age that a lack of access does not prevent most women from attaining their desired family size. The effect on the probability of remaining childless is small and statistically insignificant using the rest of Norway as a control. Using Bergen as the control county, I find a positive effect of abortion access as a teenager on the probability of remaining childless. This finding is consistent with the idea that abortion access allows women who have a desired family size of zero to attain that goal. I do not find any effect on the likelihood of finishing high school or on years of education, but I do find a positive effect on obtaining a college degree that is about 1.8% and statistically significant at the 5% level. This is substantial as the baseline probability of achieving a college degree is only 24%. I find a smaller positive effect (also significant at the 5% level) on obtaining an advanced degree of about 0.8 of a percentage point. Given that only about 3% of these cohorts obtain a higher degree, this is a large effect, but the confidence interval is quite large. I conclude that abortion access led to increases in educational investments for these women. There is no evidence for any effect on high school graduation. However, children of mothers who had access to abortion are about 2% more likely to take the academic track rather than the vocational track. I see little evidence for a positive effect of maternal abortion availability on education of their children in terms of having some college. |
| [53] [Zambia] | To compare the financial costs for women when they have an induced abortion at a facility, with costs for an induced abortion outside a facility, followed by care for abortion-related complications | Women seeking care at two public hospitals and two private clinics, one each in Lusaka and in Kafue districts in Zambia. The data were collected from women (n = 38) obtaining a legal termination of pregnancy (TOP), or care for unsafe abortions (CUA). | Mixed methods: household wealth data at one point in time (T1) and longitudinal qualitative data at two points in time (T1 and T2, three-four months later), in Lusaka and Kafue districts, between 2014 and 2015. | Identifying the source of funds women use to pay for abortion-related care enriches our understanding of the financial consequences for women of having to spend that money on abortion-related care. Women having TOPs differed in how they paid for their abortions depending on their education level. More educated women paid for the abortion with their personal funds whereas less educated women relied on families or partners to raise the funds. When men denied paternity or responsibility, the woman was left with fewer options to pay for the abortion and manage any complications that arose. It was more common among women getting TOP (compared to those who had CUA) for her, the husband, a relative, or a friend, to borrow money or sell items to provide funds to pay for the abortion. When funds were harder to come by, women in all wealth categories were more likely to have an unsafe abortion. Women who obtained CUA tended to be one to three people removed from the money that was used to pay for their abortion. (One person removed would mean that the woman had to ask for help from someone who lent her the money; three people removed would mean that she asked someone who asked someone who asked someone else and that third person provided the assistance.) In our sample, younger, less educated women experiencing post-abortion complications were most likely to be furthest from the money source; money for their abortion-related expenses came from family members borrowing money on their behalf. School-going girls often used money previously allocated for food or school-related expenses provided by their families to cover the abortion-related costs. In contrast, older women found ways to access money themselves, or borrowed directly from family. Delays in raising the funds increased the cost as well as the risk of the procedure. Women expressed frustration and anxiety about the duration of time it took them to raise the money. |
| [54] [Kenya] | To illustrate how the quality of PAC in healthcare facilities is impacted by law and government policy | Patients (n=21) and health providers (n=16) | Cross-sectional study with in-depth interviews | Women were aware of service disruptions, and they often presented to public facilities only when their conditions were too grave for further self-management and when they could not afford private facilities. Emergency care was significantly impeded by the unavailability of the right service providers for certain procedures, especially doctors, and during the night, weekends or public holidays. These capacity gaps were also evident in the number of patients who required multiple evacuations, often leading to longer hospital stays and increased the cost of care to both the patients and the healthcare system. |
| [55] [Kenya] | To evaluate the adolescents' behavior regarding induced abortion | Adolescent girls and boys ages 10-19 (n=1820 adolescent, 1048 school girls, 580 school boys, and 192 post abortion patients) | Cross-sectional prospective study | More than 70% of the girls who aborted lost school-time ranging from a few days to more than one year, and three (6.4%) of the boys' girlfriends and two (1.1%) of the post-abortion girls actually discontinued school. |
| [55] [Kenya] | To evaluate the adolescents' behavior regarding induced abortion | Adolescent girls and boys ages 10-19 (n=1820 adolescent, 1048 school girls, 580 school boys, and 192 post abortion patients) | Cross-sectional prospective study | More than 70% of the girls who aborted lost school-time ranging from a few days to more than one year, and three (6.4%) of the boys' girlfriends and two (1.1%) of the post-abortion girls actually discontinued school. |
| [56] [Nigeria] | To identify near miss events (using WHO criteria) and the proportion due to unsafe abortion among women of childbearing age in eight large secondary and tertiary hospitals across the six geo-political zones. To explore the characteristics of women with these events, delays in seeking care and the short-term socioeconomic and health impacts on women and their families. | Women of childbearing age with maternal near-miss or at risk of maternal near-miss due to unsafe abortion (n=137 maternal near miss cases, 12 interviews with those due to unsafe abortion) | Cross-sectional hospital-based study | Women were asked whether they or anyone in their household lost any income during the time they were hospitalized. Four women responded that they lost between 4,000 and 65,000 Naira (US$26-$426), whereas nine reported that someone else from their household had lost between 6,000 and 100,000 Naira (US$39-$656). Since the minimum monthly salary in Nigeria was 18,000 Naira at the time the study was conducted (US$118)17, these costs represent a huge amount of money for these women and their households. |
| [57] [United Kingdom] | To determine the acceptability, efficacy and costs of medical termination of pregnancy (MTOP) compared with surgical termination of pregnancy (STOP) at less than 14 weeks’ gestation, and to understand women’s decision-making processes and experiences when accessing the termination service. | Women accepted for termination of pregnancy (TOP) under the relevant Acts of Parliament with pregnancies < 14 weeks’ gestation on the day of abortion. (n= 1877 women, 349 in the  randomized arms and 1528 in the preference arms) | Randomized preference trial | There were no differences in time taken to return to work between groups (medical termination of pregnancy and surgical termination of pregnancy); around 90% had returned to work and normal activity by 2 weeks. |
| [58] [United States] | To examine the impact of the increase from a 24-hour mandatory waiting period to a 72-hour waiting period on the proportion of women who obtain an abortion | Women attending three family planning clinics in Utah | Assessment of data from an abortion information consultation | One half of women negatively impacted by the increase in mandatory waiting to 72 hours reported that they had to take extra time off work. 47% reported loss of wages, 18% excess childcare costs, 30% increased transport costs and 27% in additional expenditures and lost wages by a family member or friend. |
| [59] [Australia] | To examine access and equity to induced abortion services in Australia, including factors associated with presenting beyond nine weeks gestation | Women aged 16+ years attending for an abortion at 14 Dr Marie clinics (n=2,326) | Cross-sectional survey | About 22.5% reported experiencing trouble with financial costs as a challenge in accessing the service. Separately, one in three (34.0%) women reported that they found it difficult/very difficult to pay for the abortion. Just over two-thirds of survey respondents (68.1%) obtained financial assistance from one or more sources to help pay for the abortion. The husband/partner/ man involved in the pregnancy was the most commonly cited source (80.0%) of help, followed by a family member (16.0%). About 64.0% answered the question on having to forego regular payments to cover abortion costs. Among them, just over a third (35.1%) had to forego one or more payments, most often for bills (71.2%), followed by food and groceries (35.5%). " [p.312] "About 16.0% of respondents were undergoing an abortion after 9 weeks, and therefore no longer eligible for the medical option. In a multivariate model (Table 2) adjusted for age, country of birth and educational status, we found that women who had to travel four or more hours (OR: 3.0, 95%CI 1.2-7.3), who had no knowledge of the medical option (OR: 2.1, 95%CI 1.4-3.1), and who experienced difficulties in financing the abortion (OR: 1.5, 95%CI 1.2-1.9) had significantly higher odds of presenting later than nine weeks gestation. Also, women who identified as Aboriginal and/or Torres Strait Islander (OR: 2.1, 95%CI 1.2-3.4) and who received a government pension as main income (OR: 1.5, 95%CI 1.0-2.1) had higher odds of later presentation. |
| [60] [Global] | To review the scientific evidence on the consequences of unsafe abortion, highlight gaps in the evidence base, suggest areas where future research efforts are needed, and speculate on the future situation regarding consequences and evidence over the next 5–10 years. | Women who obtained unsafe abortions | Literature review | There are types of economic costs that have not been studied because of the difficulty in collecting data to document these costs. The cost of medical care for longer-term consequences of unsafe abortion, which include chronic reproductive tract infections and infertility, has not been rigorously studied so far. In part, this is because estimates of the numbers of women who will experience these consequences are themselves difficult to make; an additional factor is the difficulty of estimating the medical costs needed to treat these serious long-term health consequences because the use of the necessary medical technology has been so infrequent. Another aspect of the economic cost of unsafe abortion for which documentation is relatively weak is the impact on women, families and households. The steps that women take to obtain an unsafe abortion, to seek medical care for complications (including intermediate care before reaching the hospital, transportation and out-of-pocket expenses while in a facility), and costs after obtaining post-abortion treatment (for example for drugs or supplies) are themselves costly, and these are out of pocket expenses that the household incurs. Loss of productive time because of the health complications can also be an important consequence for the household. Again, this is difficult to quantify given the disagreement on approximating the monetary value of women’s labor and time, particularly for women who are not earning an income. Efforts for a more standardized approach to estimate the cost of post-abortion care found a need for more attention to be directed to finding ways, specific to each country context, for following up post-abortion patients. Such efforts are important given that out-of-pocket expenses and lost productivity do not end with the patient’s discharge from a hospital or health facility, and a follow-up interview is essential for measuring these aspects of the economic impact of the unsafe abortion. This pilot project produced model questionnaires and a study design that are available for adaptation and use by others. The next step is to apply these at the national level, which is underway in Uganda. |
| [61] [Kenya] | To analyze socioeconomic and reproductive background characteristics of women with incomplete abortions and assess post-abortion health consequences and financial implications for the women and the hospital. | Women seeking help for incomplete abortions  (n=281, 91 spontaneous abortions, 152 suspected induced abortions, 28 admittedly induced abortions) | Interviews | Total salary loss due to treatment for unsafe abortions was 195 KS compared to 175 KS for spontaneous incomplete abortions |
| [62] [India] | To examine reasons why girls who seek abortions are always at higher risk for delay in care seeking | Unmarried young people (10-24 years of age) seeking abortion care at the study clinic (n=34) | Cross-sectional descriptive study | Financial problems were another reason for delay. One respondent reported the pregnancy progressing to the second trimester by the time they had raised funds for the abortion which was a pregnancy resulting from rape |
| [63] [Uganda] | To measure the costs of unsafe abortion and post-abortion care to Ugandan women and their households, examining micro-level costs in three ways: women’s out-of-pocket expenditures for abortion and treatment of post-abortion complications, the impact of the complications and associated expenses on the children in the household (if any) and on the productivity of women and other family members, and the households’ economic responses to the consequences of unsafe abortion: sales of assets, incurrence of debt, and changes in income and consumption | Ugandan women who had been admitted to one of 27 health facilities for treatment of complications (n=1,338) | Prospective hospital-based survey | Social and economic outcomes: At the first interview, 60% of the 666 women who had children reported that their children were eating less, were unable to attend school or both as a result of the abortion. In addition, 73% of the women who likely had had an induced abortion stated that they or someone else in their household were already experiencing some loss of productivity. During the follow-up interviews, 34% of the 420 women who likely had had an induced abortion reported that they had experienced an economic impact from their abortion complications. Most of our independent variables were associated with one or more negative consequences. Overall, higher proportions of women who had more serious complications (i.e., had spent a night or more in a health facility) than those with less serious complications reported having experienced negative consequences—their children had suffered, they or someone in their household had lost productive employment or their economic circumstances had deteriorated. The proportion of women who reported that their children had suffered negative consequence declined with increasing age, from 92% among teenage respondents to 62% among 20–29-year-olds and 51% among older women. Women with one or two living children were more likely than women of higher parity to report that their children had been negatively affected (69% vs. 49%); however, those with at least one child were more likely than childless women to have suffered some loss of productivity (76–77% vs. 66%). The proportion of women who reported that they, their children, or someone in their household had suffered negative consequences was consistently higher among married women than among unmarried women. Women who were not attending school were more likely than those who were to report loss of productivity (75% vs 64%). Those with no more than a primary education were more likely than better-educated women to report lost productivity (77% vs. 70%). Negative consequences for children: The odds that a woman’s children had already experienced negative consequences—had had less food than usual, had missed school or both—at the time she was being treated for abortion complications were higher among women who had spent a night or more at a health facility than among women who had not had an overnight stay (odds ratios, 1.6–1.8). The likelihood of negative consequences was substantially lower among children of women aged 20 or older than among those of teenage respondents (0.2 for both older age-groups), and lower among children of women with at least three living offspring than among those of lower-parity women (0.4). Children of married women had more than twice the odds of having suffered negative consequences than did children of unmarried women (2.2). Finally, and not unexpectedly, children of women in the two wealthiest groups were less likely than those of the poorest respondents to have had any negative consequences as a result of their mother’s post-abortion complications (0.4–0.5). •Loss of productivity. Only two characteristics were associated with the respondent or other household members having experienced reduced productivity (inability to work, to earn or both) because of the respondent’s abortion-related morbidity. The odds of productivity loss among women who had spent one or more nights in a health facility (and hence probably had relatively severe complications) were more than twice those of women who had been treated as outpatients (odds ratios, 2.2–2.4). In addition, loss of productivity was more common in households of married women than in those of unmarried women (1.6). Deterioration in economic circumstances: Among women who were re-interviewed 2–3 months after receiving post-abortion care, two characteristics were associated with deterioration in their economic circumstances—that is, with whether they and their family had lost economic assets, incurred debt, lowered their consumption, or had to work more or give up their jobs in response to their illness. Women who had spent one night at the health facility had higher odds of experiencing economic deterioration than did women who had not had an overnight stay (odds ratio, 2.8) and women who had incurred higher post-abortion care expenses (i.e., those in the highest three quintiles) were more likely than those with lower expenses to have seen their economic circumstances worsen (1.6). |
| [64] [United States] | To describe the characteristics associated with being turned away because of provider gestational age limit, and the efforts women make to obtain an abortion | Women recruited in the Turnaway Study by 30 abortion providers across the US | Descriptive statistics through open and closed ended surveys | 36.5% of first trimester patients and 58.3% of turnaways experienced delays from travel and procedure costs. 37.8% of first trimester patients and 48.1% of turnaways reported delays due to insurance problems.  The most commonly cited reason for a delay was “money” or “finances”.  Of the 21.6% of turnaways who considered having an abortion, 85.4% reported travel and procedure costs as a reason for not obtaining one. |
| [65] [United States] | To investigate whether abortion enables women to achieve personal life goals | Women seeking abortion services  (n=757) | Regression analysis and qualitative methods | The 757 participants in this analysis reported a total of 1,304 one-year plans. The most common one-year plans were related to education (21.3 %), employment (18.9 %), other (16.3 %), and change in residence (10.4 %). Most goals (80 %) were aspirational, defined as a positive plan for the next year. First Trimesters (women who presented in the first trimester and received abortions) and Near-Limits (women who presented up to 2 weeks under the limit and received abortions) were over 6 times as likely as Parenting Turnaways (women with children who presented up to 3 weeks over the facility’s gestational age limit and were turned away) to report aspirational one-year plans [Adjusted Odds Ratio (AOR) = 6.37 and 6.56 respectively, p < 0.001 for both]. Among all plans in which achievement was measurable (n = 1,024, 87 %), Near-Limits (45.6 %, AOR = 1.91, p = 0.003) and Non-Parenting Turnaways (47.9 %, AOR = 2.09, p =0.026) were more likely to have both an aspirational plan and to have achieved it than Parenting Turnaways (30.4 %). Conclusions: These findings suggest that ensuring women can have a wanted abortion enables them to maintain a positive future outlook and achieve their aspirational life plans. |
| [66][United States] | To assess the incidence of abortion-related emergency department (ED) visits in the United States (U.S.): Estimated the proportion of visits that were abortion-related and described the characteristics of patients making these visits, the diagnoses and subsequent treatments received by these patients, the sociodemographic and hospital characteristics associated with the incidents and observation care only (defined as receiving no treatments), and the rate of major incidents for all abortion patients in the U.S. | Women seeking emergency care for abortion related issues (n=27,941) | Retrospective observational study | Women using Medicaid had higher odds of major incidents than those not using Medicaid and lower odds of observation care. In this context, insurance type may be a proxy for socioeconomic status, as women requiring Medicaid are low income and as a result, face a multitude of barriers to accessing health care and are known to have poorer health status, including multiple chronic conditions, than women with private insurance. Women who were self-pay were less likely to have major incidents and more likely to receive observation care only, suggesting that patients without healthcare coverage may not have been given treatments to reduce patient costs. |
| [67] [Nepal] | To assess the impact of providing accessible abortions on pregnancy decisions and sex-selection | Combined fertility histories using quantitative data | Difference-in-difference | The decreased cost of a first-trimester abortion might impact the decision to have a gender-blind abortion by women categorized as near-indifferent between perceived foetus sex. |
| [68] [Global] | To estimate the health system costs of post-abortion care in Africa and Latin America | PAC patients | Cost estimation | The indirect economic costs of unsafe abortion—those borne by households, by sectors outside the health care system and by the wider economy— are also essentially unmeasured. |
| [69] [Canada] | To compare the loss of ability to work in 62 women having medical and 69 having surgical abortions | Women seeking abortion services (n=62 women having medical and 69 having surgical abortions) | Prospective cohort questionnaire study | Outcomes included the number of days lost from work outside the home and inside the home before and after the abortion appointment. The mean total loss from work inside the home was 10.1 days for the surgical group and 5.3 days for the medical group (P < .05). The mean total loss from work outside the home was not significant at 4.0 days for the surgical group and 2.5 days for the medical group. |
| [70] [Latin America] | To summarize the findings of a literature review on women’s experiences with medical abortion in Latin American countries where voluntary abortion is illegal | Studies on women’s experiences with medical abortion in Latin American countries where voluntary abortion is illegal | Literature review | Difficulty in accessing misoprostol related to regulations and government control over sales create differing levels of obstacles, with stricter controls pushing women towards the black market where prices are higher.  In settings where prescriptions are required, strategies for accessing including obtaining a prescription from a non-ObGyn specialist or asking a man or older woman to obtain a prescription under the pretense that it is for their use. |

1. Ahmed S, Islam A, Khanum PA, Barkat e K. Induced abortion: What's happening in rural Bangladesh. Reproductive Health Matters. 1999;7(14):19-29. doi: <https://doi.org/10.1016/S0968-8080(99)90003-4>.

2. Aiken ARA, Johnson DM, Broussard K, Padron E. Experiences of women in Ireland who accessed abortion by travelling abroad or by using abortion medication at home: a qualitative study. BMJ sexual & reproductive health. 2018. Epub 2018/07/05. doi: 10.1136/bmjsrh-2018-200113. PubMed PMID: 29972360; PubMed Central PMCID: PMCPMC6237647.

3. Alouini S, Uzan M, Méningaud JP, Hervé C. Knowledge about contraception in women undergoing repeat voluntary abortions, and means of prevention. European Journal of Obstetrics & Gynecology and Reproductive Biology. 2002;104(1):43-8. doi: <https://doi.org/10.1016/S0301-2115(02)00060-X>.

4. Anandhi S. Women, Work and Abortion: A Case Study from Tamil Nadu. Economic and Political Weekly. 2007;42(12):1054-9.

5. Aniteye P, Mayhew S. Attitudes and Experiences of Women Admitted to Hospital with Abortion Complications in Ghana. African Journal of Reproductive Health / La Revue Africaine de la Santé Reproductive. 2011;15(1):47-55.

6. Appiah-Agyekum NN, Sorkpor C, Ofori-Mensah S. Determinants of abortion decisions among Ghanaian university students. International journal of adolescent medicine and health. 2015;27(1):79-84. Epub 2014/08/26. doi: 10.1515/ijamh-2014-0011. PubMed PMID: 25153553.

7. Austin N, Harper S. Assessing the impact of TRAP laws on abortion and women's health in the USA: a systematic review. BMJ sexual & reproductive health. 2018;44(2):128-34. Epub 2018/06/21. doi: 10.1136/bmjsrh-2017-101866. PubMed PMID: 29921636.

8. Asplin N, Wessel H, Marions L, Georgsson Öhman S. Pregnancy termination due to fetal anomaly: Women's reactions, satisfaction and experiences of care. Midwifery. 2014;30(6):620-7. doi: <https://doi.org/10.1016/j.midw.2013.10.013>.

9. Bailey PE, Bruno ZV, Bezerra MF, Queiróz I, Oliveira CM, Chen-Mok M. Adolescent pregnancy 1 year later: the effects of abortion vs. motherhood in Northeast Brazil. Journal of Adolescent Health. 2001;29(3):223-32. doi: <https://doi.org/10.1016/S1054-139X(01)00215-4>.

10. Baum SE, White K, Hopkins K, Potter JE, Grossman D. Women's Experience Obtaining Abortion Care in Texas after Implementation of Restrictive Abortion Laws: A Qualitative Study. PloS one. 2016;11(10):e0165048. Epub 2016/10/27. doi: 10.1371/journal.pone.0165048. PubMed PMID: 27783708; PubMed Central PMCID: PMCPMC5082726 case Whole Woman's Health v. Hellerstedt. Dr. Grossman was not compensated for his testimony; Dr. Hopkins was compensated. Lead author Sarah Baum is affiliated with Ibis Reproductive Health which is "an international nonprofit organization with a mission to improve women's reproductive autonomy, choices, and health worldwide". All authors are affiliated with the Texas Policy Evaluation Project (TxPEP), whose purpose is to document and evaluate the impact of reproductive health legislation passed by the Texas Legislature. Both Ibis Reproductive Health and TxPEP conduct rigorous research to assess the impact of reproductive health policies. This does not alter our adherence to PLOS ONE policies on sharing data and materials.

11. Baxerres C, Boko I, Konkobo A, Ouattara F, Guillaume A. Abortion in two francophone African countries: a study of whether women have begun to use misoprostol in Benin and Burkina Faso. Contraception. 2018;97(2):130-6. doi: <https://doi.org/10.1016/j.contraception.2017.10.011>.

12. Bessett D, Gorski K, Jinadasa D, Ostrow M, Peterson MJ. Out of Time and Out of Pocket: Experiences of Women Seeking State-Subsidized Insurance for Abortion Care in Massachusetts. Women's Health Issues. 2011;21(3, Supplement):S21-S5. doi: <https://doi.org/10.1016/j.whi.2011.02.008>.

13. Biggs MA, Ralph L, Raifman S, Foster DG, Grossman D. Support for and interest in alternative models of medication abortion provision among a national probability sample of U.S. women. Contraception. 2018. doi: <https://doi.org/10.1016/j.contraception.2018.10.007>.

14. Billings DL, Benson J. Postabortion care in Latin America: policy and service recommendations from a decade of operations research. Health policy and planning. 2005;20(3):158-66. Epub 2005/04/21. doi: 10.1093/heapol/czi020. PubMed PMID: 15840631.

15. Bloomer F, O'Dowd K. Restricted access to abortion in the Republic of Ireland and Northern Ireland: exploring abortion tourism and barriers to legal reform. Culture, Health & Sexuality. 2014;16(4):366-80. doi: 10.1080/13691058.2014.886724.

16. Brack CE, Rochat RW, Bernal OA. It's a Race Against the Clock: A Qualitative Analysis of Barriers to Legal Abortion in Bogot, Colombia. International Perspectives on Sexual and Reproductive Health. 2017;43(4):173-82.

17. Brown RW, Jewell RT. The Impact of Provider Availability on Abortion Demand. Contemporary Economic Policy. 1996;14(2):95-106. doi: <http://onlinelibrary.wiley.com/journal/10.1111/%28ISSN%291465-7287>. PubMed PMID: 0384757.

18. Chibber KS, Biggs MA, Roberts SCM, Foster DG. The Role of Intimate Partners in Women's Reasons for Seeking Abortion. Women's Health Issues. 2014;24(1):e131-e8. doi: <https://doi.org/10.1016/j.whi.2013.10.007>.

19. Chunuan S, Kosunvanna S, Sripotchanart W, Lawantra J, Lawantrakul J, Pattrapakdikul U, et al. Characteristics of Abortions in Southern Thailand. Pacific Rim International Journal of Nursing Research. 2012;16(2):97-112.

20. Cockrill K, Weitz TA. Abortion patients' perceptions of abortion regulation. Women's health issues : official publication of the Jacobs Institute of Women's Health. 2010;20(1):12-9. Epub 2010/02/04. doi: 10.1016/j.whi.2009.08.005. PubMed PMID: 20123172.

21. Cooper D, Dickson K, Blanchard K, Cullingworth L, Mavimbela N, von Mollendorf C, et al. Medical abortion: the possibilities for introduction in the public sector in South Africa [corrected] [published erratum appears in REPROD HEALTH MATTERS 2006 May;14(27):5]. Reproductive Health Matters. 2005;13(26):35-43.

22. Cunningham S, Lindo JM, Myers C, Schlosser A. How Far Is Too Far? New Evidence on Abortion Clinic Closures, Access, and Abortions. 2017.

23. David HP, Baban A. Women's health and reproductive rights: Romanian experience. Patient Education & Counseling. 1996;28(3):235-45.

24. Dennis A, Manski R, Blanchard K. Does Medicaid Coverage Matter? A Qualitative Multi-State Study of Abortion Affordability for Low-income Women. Journal of Health Care for the Poor & Underserved. 2014;25(4):1571-85. doi: 10.1353/hpu.2014.0151.

25. Doran F, Nancarrow S. Barriers and facilitators of access to first-trimester abortion services for women in the developed world: a systematic review. Journal of Family Planning & Reproductive Health Care. 2015;41(3):170-80. doi: 10.1136/jfprhc-2013-100862. PubMed PMID: 109585078. Language: English. Entry Date: 20150923. Revision Date: 20170104. Publication Type: journal article.

26. Dragoman M, Davis A. Abortion care for adolescents. Clinical Obstetrics & Gynecology. 2008;51(2):281-9.

27. Ely GE, Hales T, Jackson DL, Bowen EA, Maguin E, Hamilton G. A trauma-informed examination of the hardships experienced by abortion fund patients in the United States. Health Care for Women International. 2017a;38(11):1133-51. doi: 10.1080/07399332.2017.1367795.

28. Ely GE, Hales TW, Jackson DL. A cross-cultural exploration of abortion fund patients in the USA and the Republic of Ireland, Northern Ireland and the Isle of Man. Culture, Health & Sexuality. 2018;20(5):560-73. doi: 10.1080/13691058.2017.1361550. PubMed PMID: 129343735. Language: English. Entry Date: 20180906. Revision Date: 20180906. Publication Type: Article. Journal Subset: Allied Health.

29. Erfani A. Induced Abortion in Tehran, Iran: Estimated Rates and Correlates. International Perspectives on Sexual and Reproductive Health. 2011;37(3):134-42.

30. Esia-Donkon K, Darteh EKM, Blemano H, Asare H. Who Cares? Pre and Post Abortion Experiences among Young Females in Cape Coast Metropolis, Ghana. African Journal of Reproductive Health / La Revue Africaine de la Santé Reproductive. 2015;19(2):43-51.

31. Font-Ribera L, Perez G, Espelt A, Salvador J, Borrell C. Determinants of induced abortion delay. Gac Sanit. 2009;23(5):415-9. doi: 10.1016/j.gaceta.2008.08.001. PubMed PMID: WOS:000270411100010.

32. Foster DG, Kimport K. Who Seeks Abortions at or After 20 Weeks? Perspectives on Sexual & Reproductive Health. 2013;45(4):210-8. doi: 10.1363/4521013.

33. Foster AM, LaRoche KJ, El-Haddad J, DeGroot L, El-Mowafi IM. "If I ever did have a daughter, I wouldn't raise her in New Brunswick:" exploring women's experiences obtaining abortion care before and after policy reform. Contraception. 2017;95(5):477-84. doi: 10.1016/j.contraception.2017.02.016.

34. Fuentes L, Lebenkoff S, White K, Gerdts C, Hopkins K, Potter JE, et al. Women's experiences seeking abortion care shortly after the closure of clinics due to a restrictive law in Texas. Contraception. 2016;93(4):292-7. doi: 10.1016/j.contraception.2015.12.017.

35. Gallo MF, Nghia NC. Real life is different: a qualitative study of why women delay abortion until the second trimester in Vietnam. Social Science & Medicine. 2007;64(9):1812-22.

36. Hendrickson C, Fetters T, Mupeta S, Vwallika B, Djemo P, Raisanen K. Client–pharmacy worker interactions regarding medical abortion in Zambia in 2009 and 2011. International Journal of Gynecology & Obstetrics. 2016;132(2):214-8. doi: <https://doi.org/10.1016/j.ijgo.2015.07.008>.

37. Hu L, Schlosser A. Does sex-selective abortion improve girls' well-being? evidence from India. 2010.

38. Ilboudo PGC, Greco G, Sundby J, Torsvik G. Costs and consequences of abortions to women and their households: a cross-sectional study in Ouagadougou, Burkina Faso. Health Policy & Planning. 2015;30(4):500-7. doi: 10.1093/heapol/czu025.

39. Jerman J, Frohwirth L, Kavanaugh ML, Blades N. Barriers to Abortion Care and Their Consequences For Patients Traveling for Services: Qualitative Findings from Two States. Perspectives on Sexual & Reproductive Health. 2017;49(2):95-102. doi: 10.1363/psrh.12024.

40. Jewell RT, Brown RW. An economic analysis of abortion: the effect of travel cost on teenagers. The Social Science Journal. 2000;37(1):113-24. doi: <https://doi.org/10.1016/S0362-3319(99)00063-4>.

41. Jones BS, Weitz TA. Legal barriers to second-trimester abortion provision and public health consequences. American Journal of Public Health. 2009;99(4):623-30. doi: 10.2105/AJPH.2007.127530.

42. Jones RK, Finer LB. Who has second-trimester abortions in the United States? Contraception. 2012;85(6):544-51. doi: <https://doi.org/10.1016/j.contraception.2011.10.012>.

43. Jones RK, Jerman J. Characteristics and Circumstances of US Women Who Obtain Very Early and Second Trimester Abortions. PloS one. 2017;12(1):15. doi: 10.1371/journal.pone.0169969. PubMed PMID: WOS:000396167300057.

44. Jones RK, Ingerick M, Jerman J. Differences in Abortion Service Delivery in Hostile, Middle-ground, and Supportive States in 2014. Women's Health Issues. 2018;28(3):212-8. doi: 10.1016/j.whi.2017.12.003. PubMed PMID: 129626512. Language: English. Entry Date: 20180522. Revision Date: 20180522. Publication Type: Article.

45. Kiley JW, Yee LM, Niemi CM, Feinglass JM, Simon MA. Delays in request for pregnancy termination: comparison of patients in the first and second trimesters. Contraception. 2010;81(5):446-51. doi: <https://doi.org/10.1016/j.contraception.2009.12.021>.

46. Lane SD, Jok JM, El-Mouelhy MT. Buying safety: the economics of reproductive risk and abortion in Egypt. Social Science & Medicine. 1998;47(8):1089-99. PubMed PMID: 107173043. Language: English. Entry Date: 19990301. Revision Date: 20150711. Publication Type: Journal Article.

47. Lee-Rife SM. Women's empowerment and reproductive experiences over the lifecourse. Social Science & Medicine. 2010;71(3):634-42. doi: 10.1016/j.socscimed.2010.04.019. PubMed PMID: 105065306. Language: English. Entry Date: 20100827. Revision Date: 20150711. Publication Type: Journal Article.

48. Leone T, Coast E, Parmar D, Vwalika B. The individual level cost of pregnancy termination in Zambia: a comparison of safe and unsafe abortion. Health Policy & Planning. 2016;31(7):825-33. doi: 10.1093/heapol/czv138.

49. Lince-Deroche N, Fetters T, Sinanovic E, Blanchard K. Accessing medical and surgical first-trimester abortion services: women's experiences and costs from an operations research study in KwaZulu-Natal Province, South Africa. Contraception. 2017a;96(2):72-80. doi: 10.1016/j.contraception.2017.03.013.

50. Machungo F, Zanconato G, Bergstrom S. Socio-economic background, individual cost and hospital care expenditure in cases of illegal and legal abortion in Maputo. Health & Social Care in the Community. 1997;5(2):71-6.

51. Margo J, McCloskey L, Gupte G, Zurek M, Bhakta S, Feinberg E. Women's Pathways to Abortion Care in South Carolina: A Qualitative Study of Obstacles and Supports. Perspectives on Sexual & Reproductive Health. 2016;48(4):199-207. doi: 10.1363/psrh.12006.

52. Molland E. Benefits from Delay? The Effect of Abortion Availability on Young Women and Their Children. Labour Economics. 2016;43:6-28. doi: <http://www.sciencedirect.com/science/journal/09275371>. PubMed PMID: 1596811.

53. Moore AM, Dennis M, Anderson R, Bankole A, Abelson A, Greco G, et al. Comparing women's financial costs of induced abortion at a facility vs. seeking treatment for complications from unsafe abortion in Zambia. Reproductive Health Matters. 2018;26(52):138-50. doi: 10.1080/09688080.2018.1522195.

54. Mutua MM, Manderson L, Musenge E, Achia TNO. Policy, law and post-abortion care services in Kenya. PloS one. 2018;13(9):18. doi: 10.1371/journal.pone.0204240. PubMed PMID: WOS:000445329700031.

55. Mutungi AK, Wango EO, Rogo KO, Kimani VN, Karanja JG. Abortion: Behaviour of adolescents in two districts in Kenya. East Afr Med J. 1999;76(10):541-6. PubMed PMID: WOS:000087190400002.

56. Prada E, Bankole A, Oladapo OT, Awolude OA, Adewole IF, Onda T. Maternal Near-Miss Due to Unsafe Abortion and Associate Short-Term Health and Socio-Economic Consequences in Nigeria. African Journal of Reproductive Health / La Revue Africaine de la Santé Reproductive. 2015;19(2):52-62.

57. Robson S, Kelly T, Howel D, Deverill M, Hewison J, Lie M, et al. Randomised preference trial of medical versus surgical termination of pregnancy less than 14 weeks' gestation (TOPS). Health Technology Assessment. 2009;13(37):1-148. doi: 10.3310/hta13530.

58. Sanders JN, Conway H, Jacobson J, Torres L, Turok DK. The Longest Wait: Examining the Impact of Utah's 72-Hour Waiting Period for Abortion. Women's Health Issues. 2016;26(5):483-7. doi: 10.1016/j.whi.2016.06.004.

59. Shankar M, Black KI, Goldstone P, Hussainy S, Mazza D, Petersen K, et al. Access, equity and costs of induced abortion services in Australia: a cross-sectional study. Australian & New Zealand Journal of Public Health. 2017;41(3):309-14. doi: 10.1111/1753-6405.12641.

60. Singh S. Global consequences of unsafe abortion. Women's health (London, England). 2010;6(6):849-60. Epub 2010/12/02. doi: 10.2217/whe.10.70. PubMed PMID: 21118043.

61. Sjostrand M, Quist V, Jacobson A, Bergstrom S, Rogo KO. Socio-economic client characteristics and consequences of abortion in Nairobi. East African medical journal. 1995;72(5):325-32. Epub 1995/05/01. PubMed PMID: 7555891.

62. Sowmini CV. Delay in termination of pregnancy among unmarried adolescents and young women attending a tertiary hospital abortion clinic in Trivandrum, Kerala, India. Reproductive Health Matters. 2013;21(41):243-50. doi: <https://doi.org/10.1016/S0968-8080(13)41700-7>.

63. Sundaram A, Vlassoff M, Mugisha F, Bankole A, Singh S, Amanya L, et al. Documenting the Individual- and Household-Level Cost of Unsafe Abortion in Uganda. International Perspectives on Sexual & Reproductive Health. 2013;39(4):174-84. doi: 10.1363/3917413.

64. Upadhyay UD, Weitz TA, Jones RK, Barar RE, Foster DG. Denial of Abortion Because of Provider Gestational Age Limits in the United States. American Journal of Public Health. 2014;104(9):1687-94. doi: 10.2105/AJPH.2013.301378.

65. Upadhyay UD, Biggs MA, Foster DG. The effect of abortion on having and achieving aspirational one-year plans. BMC Women's Health. 2015;15(1):102. doi: 10.1186/s12905-015-0259-1.

66. Upadhyay UD, Johns NE, Barron R, Cartwright AF, Tapé C, Mierjeski A, et al. Abortion-related emergency department visits in the United States: An analysis of a national emergency department sample. BMC Medicine. 2018;16(1):N.PAG-N.PAG. doi: 10.1186/s12916-018-1072-0. PubMed PMID: 130183546. Language: English. Entry Date: 20181017. Revision Date: 20181017. Publication Type: journal article. Journal Subset: Biomedical.

67. Valente C. Access to Abortion, Investments in Neonatal Health, and Sex-Selection: Evidence from Nepal. Journal of Development Economics. 2014;107:225-43. doi: <http://www.sciencedirect.com/science/journal/03043878>. PubMed PMID: 1430628.

68. Vlassoff M, Walker D, Shearer J, Newlands D, Singh S. Estimates of health care system costs of unsafe abortion in Africa and Latin America. International Perspectives on Sexual & Reproductive Health. 2009;35(3):114-21. doi: 10.1363/ipsrh.35.114.09. PubMed PMID: 105267647. Language: English. Entry Date: 20100212. Revision Date: 20150819. Publication Type: Journal Article.

69. Wiebe ER, Janssen P. Time lost from work among women choosing medical or surgical abortions. Women's Health Issues. 2000;10(6):327-32. doi: <https://doi.org/10.1016/S1049-3867(00)00061-X>.

70. Zamberlin N, Romero M, Ramos S. Latin American women's experiences with medical abortion in settings where abortion is legally restricted. Reprod Health. 2012;9(1):34. Epub 2012/12/25. doi: 10.1186/1742-4755-9-34. PubMed PMID: 23259660; PubMed Central PMCID: PMCPMC3557184.
