# Supplementary material for: The microeconomics of abortion: A scoping review and analysis of the economic consequences for abortion care-seekers
Source: PLoS One. 2021 Jun 9;16(6):e0252005. doi: 10.1371/journal.pone.0252005 (PMC8189560; doi:10.1371/journal.pone.0252005)
Supplement: S3 Appendix — (DOCX) [file pone.0252005.s003.docx]

## S3 Appendix. Summary of studies reporting microeconomic benefits/value

| **Author, year [country]** | **Aim/objective(s)** | **Population** | **Study type** | **Summary of main findings** |
| --- | --- | --- | --- | --- |
| [1] [Bangladesh] | To find out where women go for induced abortion in rural Bangladesh today, their contraceptive practice prior to and after getting pregnant, their reasons for choosing abortion, who makes the decision for abortion, what complications they develop, and where they go for treatment for these complications | All women seeking abortion-related care at six health facilities (two rural hospitals and four static clinics) in two rural sub-districts of Bangladesh in 1996-1997 (n=143) | Semi-structured questionnaire with interviews | Twenty-two of the women (15.4%) said they were too poor to support another child. |
| [2] [Ireland] | (1) to examine the factors affecting whether women in Ireland choose to access abortion by travelling or by using online telemedicine; and (2) to explore their experiences in accessing care through each pathway | Women (n=38) identified through three organisations: Women on Web, Abortion Support Network, For Reproductive Rights Against Oppression, Sexism and Austerity.  Criteria: aged over 18, had an abortion within 8 years of study, lived in Ireland at time of abortion, had travelled or used telemedicine to access abortion care. | Qualitative in-depth interviews | Telemedicine was considered a method to mitigate the negatives of travelling for abortion care in Ireland. A respondent (age 40, 5 children) explained:  “I didn’t consider travelling at all. I don’t really get why people travel when the pill is available online. Travel just seemed very invasive and unnecessary and the pill just seems like such a sensible option.” |
| [3] [United Kingdom] | To examine reasons for seeking abortion services outside the formal healthcare system in Great Britain, where abortion is legally available | Women resident in England, Scotland, and Wales who requested at-home medication abortion through online telemedicine initiative Women on Web in 2016-2017 (n=519) | Mixed methods | "I have restrictions that could jeopardise my safety here. The pregnancy is a result of adultery. I do not want to defy certain family members and bring shame on them knowing that I depend on them. This would put my life at risk and mean that I would have to flee with no job and no skills. This [abortion] is beyond doubt the safest options for me," (p. 6). "I'm in a controlling relationship, he watches my every move, I'm so scared he will find out, I believe he's trying to trap me and will hurt me. I can't breathe. If he finds out, he wouldn't let me go ahead, then I will be trapped forever. I cannot live my life like this," (p.6). |
| [4] [Ireland] | To examine the characteristics and experiences of women in Ireland and Northern Ireland seeking at-home medical termination of pregnancy (TOP) using online telemedicine | Women who requested at-home medical TOP through online telemedicine initiative Women on Web from 2010-2015 (n=5,650). Women who completed TOP in 2010-2012 (n=1,023) | Logistic regression and content analysis of women’s evaluations | Among the 3,500 women for whom information was available on their reasons for termination, 62.1% said they cannot cope with a child at this point in their life, and 43.5% said they have no money to raise a child. "‘You gave me another chance to continue my personal battle as a single mother to create a good quality of life for my child, working hard in three jobs to make ends meet and create opportunities and potential for her future,’" (p.1212). The experiences of women in Ireland and Northern Ireland with few economic and social resources suggest a grave inequity in their ability to access safe TOP. Women noted their inability to afford travel abroad, to take time away from children and work, to arrange travel without their families’ knowledge, or to travel at all due to migrant status. For some women the need to travel would have caused long delays in accessing care, and for others, it would have rendered care completely impossible. Online telemedicine goes at least part of the way towards resolving this disparity in reproductive health and rights. As one woman explained: ‘There is no way I could have afforded to travel to England, pay for the procedure, stay in a hotel, and have someone there to support me," (p. 1212). |
| [5] [Turkey] | To show that early medical abortion could be introduced safely in Turkey to improve women’s access to services | Women between the ages of 18 and 49 who had an intrauterine pregnancy up to 56 days LMP, lived or worked within one hour of study site, willing to return for at least one follow-up visit and no known allergies to mifepristone or misoprostol (n=470) | Descriptive clinical study | Women opted for home use of misoprostol rather than clinic use since transportation was a problem (7.6%). Among women preferring surgical abortion, 48% said the most important reason for this preference was that it takes a short time. Seven in ten surgical abortion patients said that one of the best features of surgical abortion is that it is done quickly. Among medical abortion patients, 62% reported no surgery as one of the best features, and 25% reported being at home as one of the best features. |
| [6] [Canada] | To potentially cast light on the role of culture in sex selection among South and East Asian immigrants in Canada | Canadian residents who participated in the 2001 and 2006 censuses | Analysis of census microdata | High and rising sex ratios lead one to ask what causes parents to prefer sons to daughters. One argument emphasizes socioeconomic and institutional factors. Absent couples’ ability to save or to rely on national pensions, the poor count heavily on children for old-age support, a task that under patriarchal norms falls on sons. In India, high and rising dowry payments are argued to place families with daughters at a disadvantage, and families may depend on males for physical protection. In both India and China, however, sex ratios are highest in the richest areas, and for India a strong education gradient is evident, with better-educated parents favoring sons more extensively. These observations cast doubt on sex selection being the result of economic necessity alone. |
| [7] [France] | To determine whether women undergoing repeat abortions are exposed to risk factors that might be amenable to preventative measures and the methods employed by carers in these cases | 30 women who had undergone two abortions prior to or in 1997 at the Family Planning Centre of Hospital Jean Verdier in Bondy, France.  The care team: two gynaecologists, two marital counsellors, two nurses and a senior nursing officer | Evaluation using a questionnaire for women and interviews with the care team | These immigrants are generally economically successful and transplanted into a developed country in which many of the commonly invoked motives for selecting sons (poverty, old-age support, one-child policy) are rendered irrelevant. |
| [8] [United States] | To compare women's needs and preferences in abortion to those in birth  To examine ways in which women's needs and preferences in abortion care differ from intrapartum care | Women who had individually experienced both birth and abortion (n=20) | Semi-structured intensive interviews and a validated Individual Level Abortion Stigma scale (ILAS) assessment | Women value determining the outcome of their pregnancy and being respected as decision-makers by providers. |
| [9] [Ghana] | To explore and understand the reasons why women terminate their pregnancies and their experiences of seeking services in order to know what and how to reform services to reduce unsafe abortion | Ghanaian women who had been admitted with complications from induced abortion (n=131) | Semi-structured hospital-based survey | Almost all respondents said their pregnancies were unplanned and two-thirds said their pregnancy was unwanted. When asked for what reason the pregnancy was unwanted, economic reasons (25%) were the second largest reason. Almost one-third of respondents, however, said they did want their pregnancy. Of these, 34% said that they had aborted because of economic problems. Among the 67 women who said their partners were unhappy with the pregnancy, one third said their partners mentioned financial constraints, and 12% said it was to avoid disrupting the girls’ educational or career opportunities. The 53 respondents who reported their partners were happy with the pregnancy were asked the reasons why they terminated their pregnancy despite this. Fifteen percent said they aborted because they were not ready to have a child or were nursing another child. Other reasons given included financial constraints, academic/career pursuits, and a range of socio-cultural issues like not ready to be second wives and unstable marriages. |
| [10] [Ghana] | To explore factors that are likely to influence abortion decisions among university students in Ghana  To explores university students’ knowledge and perceptions on abortion | Randomly sampled students at the University of Ghana (n=142) | Focus group discussions | Economic considerations appear to be the fourth most important consideration of the students. Interestingly, the availability of financial support from their partner, friends, or family during the term of the pregnancy and after the birth of the baby appears to be of much concern to the students. Additionally, the indirect costs of the pregnancy and childbirth including how the pregnancy and the birth of the baby is going to affect their income and spending levels, sources of income, cost of living, standard of living, economic activities engaged in, as well as other opportunity costs of the pregnancy, have an influence on their decisions. Apart from that, emphasis was also placed on the direct costs of the pregnancy and childbirth, such as the costs of healthcare and medication, feeding, clothing, and housing. Evidence from this study suggests that the economic circumstances of both partners and, to an extent, the families of both partners are key in decisions to abort. |
| [11] [Brazil] | To determine social and behavioral consequences of pregnancy and how these differed according to the pregnancy outcome (live birth or abortion) one year after the event | Pregnant teens who sought prenatal care at the Adolescent Clinic, and a cohort of girls of the same age who attended the same hospital but were admitted to emergency services with complications from abortion (total n=418) | Cohort studies | Adolescents who had induced abortions were 6.87 times likelier (C.I. 2.95-16.01) to be a student one year after the baseline than adolescents with intended pregnancies (reference group). |
| [12] [India] | To understand the pathways through which unsafe abortion leads to post-abortion complications and to describe the experiences of women seeking care for post-abortion complications in Madhya Pradesh | Women of reproductive age who were seeking care for post-abortion complications between August and December 2007 at one of 10 hospitals | Analysis of data from structured and provider-friendly patient registers  A semi-structured questionnaire for conducting a detailed interview with participating women | When asked why they had chosen to terminate the pregnancy, nearly three quarters of respondents indicated they did not want another child at this time (73%). Other common reasons included inability to afford another child (8%), husband not wanting another child (7%) or health problems (5%). |
| [13] [Cuba] | To explore why abortion persists as a widespread form of fertility control in Cuba despite the country’s stable low fertility and high contraceptive-use rates | Patients presenting for an appointment a large obstetrics hospital in one of the municipalities of Havana  Men who were accompanying their partners | Small-scale qualitative study using a series of in-depth interviews | Respondents’ decisions to terminate a pregnancy were often influenced by their poor economic circumstances. Carrying through with an unplanned pregnancy was not considered to be a viable option. Many lived in a multigenerational household within a small residence, and commonly reported a lack of physical space and privacy as a key factor in their decision to obtain the abortion. Bearing and rearing an “extra” (unplanned) child in light of their financial circumstances made no sense to them. In some instances, participants expressed regret about having to terminate the pregnancy and indicated a desire to have a larger family if they were living under better circumstances. Despite often wanting to have a family, the impracticality of having a child under their current circumstances rendered abortion the logical decision. |
| [14] [Thailand] | To examine the need for provision of reproductive services to displaced Burmese migrant workers along the border with Thailand | Burmese women with post-abortion complications (n=43).  Burmese lay midwives from urban and rural areas (n=15).  Male partners of women with post-abortion complications (n=10). Health workers (n=20).  Community members | Ethnographic study including a retrospective review of medical records, directed group discussions, semi-structured interviews, and informal conversations | Many Burmese migrant women are in Thailand to earn money. Hence, concerns about earning capacity, debts, the need to send remittances, the costs of raising a child and threats of sacking and deportation by bosses if pregnant were common reasons given by women having abortions. Some women spoke of the pressure they received from their families to abort because of their economic situation. Women’s right to work for equal pay, right to work during a pregnancy or any notions of maternity leave is non-existent for Burmese workers in Thailand. As most women interviewed did not have a work permit, they were a particularly vulnerable population. |
| [15] [Indonesia] | To examine the experience of premarital pregnancy and induced abortion among young, single women in Lombok, Eastern Indonesia | Single women and their families, and health care providers in Mataram, the capital city of Lombok, between August 1996 and February 1998 | Ethnography | Marriage prospects, abandonment by their partner, single motherhood, early cessation of education, and an interrupted income or career were clearly undesirable options. For single women who lacked the choice of marriage, only abortion allowed them to maintain their status as ‘good women’ and to avoid compromising their futures. |
| [16] [United States] | To understand the support for and interest in various alternative provision models for medical abortion among a diverse group of women, including women who may prefer not to access or have limited access to facility-based abortion care | A U.S. national, probability-based representative sample of women ages 18–49 online | Multivariable analyses | For advance provision, OTC and online access, women reported ‘could be less expensive’ as a potential advantage (35%, 34% and 32%, respectively). For advance provision, OTC and online access, women reported ‘could be more expensive’ as a potential disadvantage (16%, 20% and 18%, respectively). |
| [17] [Colombia] | To identify the key barriers to legal abortion, and to explore the ways they may work separately and together to delay the receipt of high quality, legal abortion care | Women who had obtained a legal abortion in Bogotá, Colombia in the last 12 months; were aged 18 or older; and exhibited verbal proficiency in Spanish (n=17) | In-depth interviews | One participant, a 39-year-old single mother, said that her partner had ended their relationship when she told him she was pregnant. She could not financially support a second child without the help of a partner and was very upset to be ending the unexpected pregnancy. Another participant said she was not emotionally or economically ready for a baby, but because she physically was able to give birth, she struggled with the decision to terminate. The women whose abortion had been paid for by their health insurance company said issues with the company had delayed their abortion. Health insurance representatives abruptly hung up when participants mentioned abortion, did not return their phone calls or told women that abortion was not covered. Inconsistent instructions from the companies regarding necessary authorizations further delayed these women from getting approval and obtaining a timely abortion. Some women were delayed by as much as two months. Insurance companies acted as a barrier to timely access to legal, safe abortion care, even though they are legally obligated to authorize the procedure. Religious sentiments appeared to underlie the behavior of company representatives. |
| [18] [Norway] | Aims:  (1) To identify the most important reasons for having an induced abortion  (2) To study the relationship between the reasons for abortion and emotional distress 6 months and 2 years after the abortion  (3) to identify the most important predictors for emotional distress | 80 women who had induced abortions at the Department of Gynaecology of the Buskerud Hospital, Norway | Semi-structured interviews and questionnaires | Negative feelings towards abortions were statistically significantly associated with women who sought abortions for financial reasons. |
| [19] [global] | To present the reasons women give for obtaining induced abortions in 14 countries | Three types of data sources from 14 countries: (1) official statistics; (2) population-based surveys of reproductive-age women (15–49 years); and (3) facility-based data collected from abortion patients | Descriptive quantitative survey data | In six of the 13 countries for data existed on the main reason for abortion, the most commonly reported reason for having an abortion was socioeconomic concerns, cited by a plurality of women (ranging from 27% to 40%). In five countries, limiting childbearing was the most frequently reported reason, ranging from 20% in Nepal to 64% in Azerbaijan. In Belgium, the most frequently cited reasons were partner-related (23%) and socioeconomic concerns (23%), and in Kyrgyz Republic, risk to maternal health was commonly reported (44%). |
| [20] [United States] | To examine how partners figure in women’s abortion decisions, and to identify factors associated with identifying partner as a reason (PAR) for abortion | Pregnant women aged 15 years or older recruited from 30 U.S. abortion facilities (January 2008-December 2010), where no facility nearby offered care at a later gestational age of pregnancy. Participants were English or Spanish speaking, no known fetal anomalies or demise, and belonged to one of the following three groups: 1) Women just over the facility’s gestational age limit and denied an abortion (n=231); 2) women just under the limit and who received an abortion (n=452); and 3) women receiving a first trimester procedure (n=273) | Mixed methods study using in-depth interviews and baseline data from the on going Turnaway Study, a prospective longitudinal study | Twenty-six percent of women said that their partner was unable or unwilling to support them in having a baby. Reasons for feeling this way included the following: partner was not financially able to provide for a child; and partner did not support the woman in raising their existing children and was therefore unlikely to support her with a new baby. Thirty-nine percent of women identifying lack of partner support in having a baby also emphasized their own financial inability to raise a child at the time. |
| [21] [Thailand] | To obtain data from women from southern Thailand who had undergone a recent abortion (spontaneous, therapeutic and unsafe) regarding: pregnancy history; number of abortions and cost of abortion related treatments; abortion complications, impacts and related health care services; reasons for having an unsafe abortion; and, circumstances related to an unsafe abortion | Thai females, of any age, who: were able to speak, write and understand Thai; had experienced an abortion, regardless of chronological age, gestational age or type; and were admitted to one of the study site hospitals | Descriptive study involving a questionnaire | Unsurprisingly, a higher proportion of younger women, compared to older women, identified socio-economic problems as reasons for an abortion. Most likely this was because younger women do not have the same social or economic resources available to older women. |
| [22] [United States] | To explore abortion patients’ perspectives on abortion regulations (e.g., mandating waiting periods, mandating the provision of state-authored information, prohibiting private and public insurance coverage for abortion) | Abortion patients at the only three high-volume abortion facilities in two states in the U.S. South and Midwest (n=20) | Qualitative study using semi-structured interviews with patients and providers | The reasons for choosing abortion among the women in our sample were diverse, but for most they matched the general categories identified in recent research ‘...that she could not afford a baby now..." |
| [23] [United States] | To answer the following questions:   1. What do women know about the cost of abortion and the availability of Medicaid coverage for abortion? 2. Where do women obtain this information? 3. What are women’s experiences paying for care? | Low-income women, defined as meeting the Medicaid income qualifications of the state where they had the abortion, aged 18 years or older who had an abortion within the past two years and who resided in one of the four study states at the time of the abortion | Two similarly-focused studies using in-depth interviews | When full coverage of abortion in the Medicaid program is available, there is little to no need for women or others in their lives to make financial sacrifices, and there is rarely a scramble for money that provokes feelings of indignity or delays abortion care. More work is needed to determine if the findings about the benefits of available abortion coverage hold true across the few states where this coverage is available and if there are other unidentified benefits. |
| [24] [Mexico] | To identify the perceptions and opinions of people who provide abortion services in Mexico City, three years after implementation of elective abortion legal reforms | 19 health workers assigned to the legal abortion programs at a clinic and a hospital in Mexico's Federal District | In-depth interviews | The majority of the participants considered that the services influence the reduction of maternal mortality rates that result from poor abortion practices, and facilitate access to services mainly to poor women. |
| [25] [New Zealand] | To examine the extent to which young women who had an abortion prior to age 21 showed advantaged outcomes when compared with women who became pregnant but did not seek an abortion | A birth cohort of young women in New Zealand studied to the age of 25 years (n=492) | 25-year longitudinal study with interviews at ages 15, 16, 18 and 21 about their pregnancy and abortion experience since the previous assessment | Prior to adjustment for confounding factors, compared with women who had become pregnant but had not had an abortion, those who had had an abortion had relatively advantaged outcomes on most measures of educational achievement, income, avoidance of welfare dependence and partnership relationships. At first sight, these findings may suggest benefits of abortion. However, subsequent analyses suggested that the differences were largely explained by the fact that those who had sought abortion were a more socially and educationally advantaged group prior to pregnancy. When due allowance was made for pre-pregnancy factors, only the educational differences between pregnant women seeking and not seeking abortion remained statistically significant. Furthermore, educational outcomes were similar among those who had had an abortion and those who had not become pregnant. Our results clearly suggest that having an abortion mitigated the educational disadvantage associated with early pregnancy. For the women in our sample, perhaps the choice to have an abortion allowed greater freedom to pursue educational goals. At the same time, similar advantages did not extend to the areas of income, welfare dependence and partnership outcomes. |
| [26] [Spain] | To: (a) know the prevalence of abortions in the immigrant population of an urban center of primary care health in the state of Murcia; (b) know the personal attitude before this population's abortions; and (c) analyze this population's socio-economic and religious characteristics and to value their possible relationship with the abortion | Women at a primary care center in San Andreas State of Murcia (n=230) | Descriptive traverse study | The fundamental reasons for which the women aborted are the lack of financial means, the pressure to which they are subjected by their partner or their family to abort, and in the third place because pregnancy and maternity constitute an inconvenience for their personal projects. The lack of financial means is related to their social instability, sometimes in an irregular situation, job instability, fear of losing their job due to the fact of being pregnant, fear of not having enough means to care for the children, or not being able to fulfill the commitment acquired sometimes to send money to their countries of origin, according to data coinciding with other studies.  In fact, 30% of the women surveyed live alone or with children, without a partner or other supportive family members. It is a reality that in Spain there are no adequate social benefits for maternity protection in terms of working hours, maternity leave, enough subsidized day care centers, aid for families with children, etc. Seventy percent of respondents who have aborted believe that they would not do it again and the reasons given are greater stability in their social, economic and family situation and having suffered the consequences of abortion. Twenty-seven percent would abort again and of the reasons given the first is social instability. The authors believe that immigrant women see their maternity determined according to socioeconomic patterns linked to their labor and social status of 'labor' subject to demands of temporality and precariousness. |
| [27] [United States] | To understand women's reasons for having abortions among women who have already made the decision to have an abortion | Survey: women arriving for a pregnancy termination (in English and Spanish)  In-depth interviews: all abortion patients had a chance to participate; recruitment was not based on social or demographic characteristics; was limited to fluent English speakers | Structured survey and in-depth interviews | Among the survey respondents, the two most common reasons for having an abortion were “having a baby would dramatically change my life” (74%) and “I can’t afford a baby now” (73%). The most common sub-reason given was that the woman could not afford a baby now because she was unmarried (42%). Thirty-eight percent indicated that having a baby would interfere with their education, and the same proportion said it would interfere with their employment. In a related vein, 34% said they could not afford a child because they were students or were planning to study. In the in-depth interviews, the three most frequently stated reasons were the same as in the structured survey: the dramatic impact a baby would have on the women’s lives or the lives of their other children (32 of 38 respondents), financial concerns (28/38), and their current relationship or fear of single motherhood (21/38). Roughly equal proportions of women in both the 1987 and 2004 surveys indicated that a baby would dramatically change their lives, that they could not afford a baby now, that they did not want to be a single mother or had problems with their relationship, and that they were not ready for a child or another child. On the other hand, smaller proportions of women in 2004 than in 1987 said that having a baby would interfere with their job or career (38% vs. 50%). In both 1987 and 2004, unreadiness for a child or another child and inability to afford a baby were each mentioned by about one-quarter of women as their most important reason for having an abortion. In contrast, the proportions reporting fear of single motherhood or relationship problems, and reporting that a child would interfere with school or career, both declined. Among women who gave at least two reasons, the most common pairs of reasons were inability to afford a baby and interference with school or work; inability to afford a baby and fear of single motherhood or relationship problems; and inability to afford a baby and having completed childbearing or having other people dependent on them. |
| [28] [Guadalupe] | To analyze, in the Guadeloupian context, the characteristics of underage people who ask for an abortion | Minor patients who have had an abortion | Descriptive, retrospective study reviewing medical records and interviewing the psychologist | Continuation of studies was the most frequently cited motive for the decision of abortion. A pregnancy and a child clearly appear as a brake on this objective of training. It is now recognized that the evolution of the statute woman's social status leads her to delay the age of motherhood, in favor of continuing education. The second reason most often found was the young age. Indeed, some of our patients felt they were too young to become mothers. |
| [29] [United States] | To analyze data on women who sought and received an abortion at or after 20 weeks’ gestation for reasons other than fetal anomaly or life endangerment | People who were seeking abortions after 20 weeks gestation across 16 sites (n=272) and people who presented for first trimester abortions (n=169) | Mixed methods – qualitative data from interviews and quantitative data for logistic regression | Provides the following case study of Angel [a pseudonym] in Maryland, who they describe as representing 47% of the sample:  “At the time of her abortion, she had a 10-month-old daughter, whom she cared for full-time while she looked for paid employment. Her husband had recently been incarcerated, leaving her with no household income. As Angel explained, her daughter was her top priority. When she realized she was pregnant, at 22 weeks, her principal concern was for her daughter. Angel’s experience of being a new mother interfered with her ability to detect her pregnancy and, moreover, convinced her that having another child was a bad idea. She believed that having another child would compromise the care she could give her infant daughter: “I knew I couldn’t continue with [the pregnancy]. My daughter isn’t even a year.” Deciding to have the abortion was very easy for Angel. |
| [30] [India] | To explore adolescent women's access to abortion services, decision-making on abortion, determinants of provider choice and extent of morbidity experienced. | Women (primarily those currently married) who had undergone an induced abortion in the study area in an 18-month reference period during 1996–1998 | Questionnaires and a qualitative in-depth time-line of sequence of events | Adolescent married women mentioned their previous child being too young as the most common reason for having an abortion, while the adult women cited not wanting any more children. A small number of adolescent abortions were to delay a first birth; all 14 women who aborted their pregnancies for this reason were either employed, studying in college or doing a vocational training course. |
| [31] [United Kingdom] | To measure women's preferences and strength of preferences (expressed in terms of willingness-to-pay) for medical abortion versus surgical vacuum aspiration | Women prior to having, and following, termination of early pregnancy (n=50) at a gynaecology out-patient clinic in Aberdeen Royal Hospitals NHS Trust between August 1993 and February 1994 | Willingness to pay technique | Thus, 48 participants gave a total willingness to pay (WTP) value of £4,211. The median WTP value (with 25th and 75th percentiles) was £50 (£26, £100), whilst the mean WTP (with 95% confidence interval) was £87 (£44, £130). Of the women who preferred the medical method, the total value given was £3,530, the median value for this group being £50 (£30, £100) whilst the mean was £103 (£43, £163). Of those preferring the surgical method the total value given was £681, the median value for this group being £50 (£5, £100), whilst the mean was £48 (£29, £67). The amounts given for each group are similar. There are, however, a minority of women who gave higher values for the medical method, and thus for those women their preference for that method was more intense. The results of the regression analysis reveal that the rating of the importance of choice and social class grouping are the best predictors of WTP. It seems that those with a greater ability to pay do not have systematically different preferences from those with less ability to pay. In general, values offered at follow up for both medical and surgical methods are similar, as well as similar to the valuations for the pre-termination preferences. |
| [32] [South Africa] | To examine the acceptability of medical abortion among young people in Durban, South Africa.  To investigate the potential demand for and applicability of the method among women in South Africa | Sexually active women at the University of Durban and under 30 years of age (n=20) | Qualitative in-depth interviews | Medical abortion presents the opportunity to save hugely in financial and time resources for medical staff. |
| [33] [United States] | To examine the impact of increased abortion availability on the average living standards of children through a selection effect | States with early adoption of abortion access vs. states with later adoption of access | ‘‘Differences-indifferences’’ strategy to estimate the effect of abortion legalization on average living circumstances | The most important change in government fertility policy over the past 30 years was the legalization of abortion under the Roe v. Wade decision. The change had a signiﬁcant effect on the living circumstances of the cohorts who were born after legalization. Subsequent cohorts were less likely to be in single-parent households, and as a result less likely to live in poverty, and less likely to receive welfare. In addition, these cohorts experienced lower infant mortality. In particular, for the marginal child not born due to increased abortion access, the odds of living in a single-parent family would have been roughly 60% higher, the odds of living in poverty nearly 50% higher, the odds of welfare receipt 45% higher, and the odds of dying as an infant 40% higher. Perhaps more importantly, these ﬁndings may also have implications for the lifelong prospects of the average child born after legalization. The children not born due to abortion availability would have grown up in adverse living circumstances that other studies have shown may be detrimental to later prospects. Of course, this conclusion is complicated by the fact that we cannot necessarily apply the effects on the average child of living in poverty (for example) to the effects on the marginal child who would live in poverty if their pregnancy were not terminated. However, as these cohorts age, researchers will be able to directly observe outcomes such as educational attainment, income, and family structure. The evidence strongly suggests that abortion is used by women to avoid bearing children who would grow up in adverse circumstances. |
| [34] [United Kingdom] | To describe and compare health outcomes two years after medical abortion or vacuum aspiration. | 140 women who participated in a randomized study in the Grapian Region, Scotland. | Structured interviews | 90% of women in the medical groups of the randomized study and 86% in the surgical groups would be willing to pay for their method – a median of 311 GBP (range 40-500 GBP). This was considered proxy for importance of method choice. |
| [35] [Australia] | To understand better rural women’s experiences in obtaining a medical termination of pregnancy (MToP) through a rural primary healthcare service in Victoria, Australia | Women aged 16 years and over who attended the clinic between February 2016 and 2017 for an appointment related to MToP | Semi-structured interviews | Women’s reasons for choosing MToP in preference to surgical termination varied greatly but the most common responses highlighted the convenience and flexibility of MToP and the ability to time it around work and life commitments; the less invasive nature of the process; and that the clinic was the closest location providing MToP outside of urban areas. |
| [36] [United States] | To determine if women's Temporary Assistance for Needy Families (TANF) participation may magnify the cues that state policy choices, the accessibility of abortion providers, and public attitudes send to all women about abortion | All respondents had borne at least one child; the sample disproportionately captured unmarried women, women of color, urban women, and those of low socioeconomic status because the study sampled from hospitals where large numbers of single mothers delivered | Probit regression | In pro-choice states, new TANF recipients are estimated to be statistically significantly more likely to resolve a pregnancy in abortion than are other comparable low-income women. The analysis suggests that this relation weakens and reverses itself as the state’s stance on abortion grows more pro-life. If TANF works at all as an alternative to abortion, it works that way only for the very most economically marginal women. This analysis addresses speculation that welfare is pro-life, reducing abortion by improving poor women’s economic capacity to choose childbirth.  The results provide evidence for this hypothesis, but that evidence is found only in states where public opinion, policy choices, and a scarcity of abortion providers signal a preference for birth over abortion. In other words, the state abortion rights climate moderates the relation between welfare and pregnancy decision-making. The study argues that this finding may be due to interstate differences in the types of economic resources that welfare provides to disadvantaged women and to the types of messages (about the acceptability and desirability of abortion) that women may receive. It also argues that those messages are magnified through participation in the TANF program. In theory, public assistance in states with pro-life stances on abortion reduces the material cost of childbearing by offering a set of basic resources that other disadvantaged women may struggle to secure. The state does not limit welfare recipients’ abortion access any more than its policies constrain access for other disadvantaged women, but neither does it offer resources like Medicaid that may help disadvantaged women obtain abortions. This study’s results imply that expanding eligibility for TANF may reduce abortions, at least in states where a pro-life stance prevails. But these results should be read very cautiously since data limitations prevent this study from testing some very plausible alternative explanations. One question that these results invite concerns the consistency of low-income women’s pregnancy decisions with their true reproductive preferences. |
| [37] [Nigeria] | To profile the characteristics and health conditions of the clientele of traditional birth homes (TBHs) in four rural communities in southeastern Nigeria | Users of TBHs and traditional birth attendants (TBAs) in rural southeastern Nigeria | Qualitative interviews | Local girls and women are attracted to the TBHs largely because of the sociocultural and economic responsiveness of TBH services. Women are attracted to TBHs because the services are low cost, the women require privacy about their conditions, the TBHs are close by, and the women are confident in the abilities of TBHs. Rural women are bound by poverty, culture, and local values in their choices of services. |
| [38] [India] | To shed light on the experiences of unmarried young abortion-seekers aged 15–24, compare their experiences with those of their married counterparts, and explore the proximate factors leading to delays in them obtaining abortions into the second trimester | Survey of abortion seekers aged 15-24 years at facilities in two poorly developed neighbouring states in north India with weak health systems, Bihar and Jharkhand (n=795)  In-depth interviews with selected unmarried survey respondents (n=26) | Survey and in-depth interviews | Thirty-seven percent of married (n=246) respondents reported economic reasons for abortion. |
| [39] [United States] | To examine the breadth of barriers, beyond those related to individual state-level abortion restrictions, that such women encounter and any associated consequences | Patients seeking abortion services (n=29) | In-depth interviews | "Concerned about how a pregnancy and giving birth would impact her role as caregiver for a sick family member, her own health and her job, Julia had decided to terminate the pregnancy." Because women often cite reasons for abortion such as an inability to afford a child, the desire to better care for existing children, and the wish to maintain their job or finish school, it stands to reason that being prevented from having a wanted abortion could adversely affect mental health and wellbeing. The authors’ findings support this idea. |
| [40] [Vietnam] | To explore the circumstances of abortions from women's perspectives | All women in the two villages who had had an abortion during 1991 | Analyses of public health data and interviews | The most common reasons given for having an abortion were financial, such as wishing to save money to build a house or wanting to avoid being fined for having too many children. One woman recalled: ‘My husband was even more concerned than me. He was afraid of having to pay the fine if we had a fifth child. He complained that the family was not rich enough to feed so many mouths.... He wanted me to have an abortion, and finally I complied with his request.’ |
| [41] [Romania] | To combine quantitative data from a previous report of the research with women's own words about the following issues: their decisions to have an abortion, the impact of abortion restrictions under the Ceausescu government, and their needs and desires for improved reproductive health services. To present gynaecologists' views of abortion restrictions and needs for improved family-planning services to make a compelling case for the need for safe, legal, comprehensive abortion care in Romania and elsewhere | Women who were seeking or obtained an abortion | Qualitative interviews and focus groups | Interestingly, most women's decisions to terminate their pregnancies were based not on their fear of immediate or delayed physical complications, but rather on their immediate economic difficulties or simply their (and/or their spouse's) unpreparedness for another child. Respondents' expressions of economic difficulties ranged, including a woman who said, ‘We have a new apartment and we have to work.’ |
| [42] [United States] | To fill in the gaps about how women pay for abortion services. To provide insights into why women with private health insurance do not use it to pay for abortion services and the role of financial assistance in subsidizing these costs. To examine the ancillary expenses that many abortion patients incur and how women feel about requesting financial support | Clinic patients seeking an abortion or having an abortion follow-up appointment at purposively sampled facilities, aged 15 or older, and able to speak English or Spanish | Survey | Substantial minorities of women who obtained money from abortion funds and family members also characterized the experience as “lifesaving,” although few women who obtained money from men indicated this response. |
| [43] [India] | To seek information from a survey about the experiences of unmarried young women in India who seek to terminate an unintended pregnancy | Unmarried women seeking abortion | Survey of women seeking abortion | One factor that is clearly associated with prompt abortion-seeking is the support of the partner. Our findings showed that partners were the leading source of support for young women. Indeed, women whose partner did not fully support them were more likely than those whose partner gave both emotional and financial support to have had a second-trimester abortion rather than one in the first trimester. We also found that young women who confided in and were accompanied to the facility by their mother were more likely to obtain a second-trimester abortion than a first-trimester procedure, and this may be explained by how women seek assistance. For example, we  hypothesize that young women are most likely to confide in their partner, and if he does not provide emotional or financial support, or if the respondent is not in a position to involve her partner, she would then turn to her mother, other family members or friends. Hence, women who had a supportive partner were more likely than others to have obtained first-trimester abortion, whereas lack of partner support—and consequent reliance on the mother and others—delayed the abortion-seeking process into the second trimester. It is notable that young women were reluctant to confide in their father or other male relatives, and that overall these family members were less likely to offer support than were partners, mothers, other female relatives ends. |
| [44] [sub-Saharan Africa] | To provide a basis for continued policy dialogue and reform to address the problem of death due to abortion complications among the East, Central and Southern Africa Health Community countries | Abortion complication patients and health providers at selected districts and tertiary care hospitals in three countries (Uganda, Malawi, Zambia) | Literature review    Reviews of logbook data and interviews | In Uganda, 76% of the providers (80% of the doctors) indicated economics as a reason for women seeking abortion. Overall, there was a significant difference (p=0.001) between the proportion of doctors (60%) versus nurses (25%) who felt that economics was a reason why women in their hospital seek an abortion. |
| [45] [Netherlands] | What are the most important motivations and reasons for delay of women who have had a second trimester abortion? Are there specific sub-groups of women who are more likely to have later abortions? Are there aspects of abortion service delivery that cause delays in obtaining an abortion until the second trimester? | Consecutive files of women at three different clinics in 2007: at clinic A, 100 women with a pregnancy of 18+ weeks, of whom 70 came from abroad; at clinic B, 94 mostly Dutch women with an abortion between 12–22 weeks of pregnancy, of whom 15 came from abroad; and at clinic C, 60 women up to 14 weeks of pregnancy, of whom 8 came from abroad | Review of clinical records and registry data | Reasons for having an abortion that were much less frequent include spacing of children, study/work, no wish for children, older age, health or financial problems. Often several reasons were mentioned, for example, too early, no finances, still a student and no stable relationship were often combined. |
| [46] [United States] | To examine the impact of state policies along with other barriers when women seek abortion care | Women in South Carolina who obtained an abortion | Interviews | Many women spontaneously described multifaceted reasons for their decision. Most described being unprepared to care for a new child in the manner they would like. Often their unreadiness was related to unstable finances, employment or housing, or to their relationship with the man involved in the pregnancy. A 28-year-old white woman who lived in a rural area noted that her boyfriend had three roommates and that she didn’t “make enough to pay all of my bills [while living] at my grandmother’s house, so there’s no way that we would have been able to afford a little one at the moment.” A 20-year-old multiracial woman who lived in an urban area shared similar concerns: “[I’m] already struggling with my first child. In all reality, I got to look at the what-ifs and how things are going to be along down the line. Working a minimum-wage job and trying to take care of me and my child and [not having] my own place is already really hard. I know how it would be with two [children], so that’s really it.” A few women focused on a desire to preserve the capacity to continue their education or career. Some women said that they either felt unready for or were finished with childbearing. |
| [47] [United States] | To estimate the demand for abortion by teenagers (ages 15–19) for the year 1992 | Teenagers and their abortion demand | Pooled time-series estimates | Teenage women have 46 more abortions per 1,000 pregnancies in Medicaid states. Labor force participation is significantly positive while Christian Fundamentalism is significantly negative. Consistent with the cross-section results, marital status is not a significant determinant in a teenager’s abortion decision. Teenage abortion decreases during recessions and increases during expansions. Education is not a significant determinant of teenage abortion demand. The AFDC benefits variable is positive and statistically significantly different from zero. In the long run, decreases in AFDC benefits decrease teenage abortion demand. This result is consistent with the work of Akerloff, Yellen, and Katz (1996) who argue that decreases in the financial incentives for having a child may result in a long-run modification in teenage sexual behavior. |
| [48] [United States] | To analyse abortion demand by black women | Black women in cross-sectional state data from 1992 | Econometric analysis of time series data | Abortion is a normal good with respect to income for black women. The income elasticity of demand, evaluated at the sample mean, is 1.25. The estimated black income elasticity of demand figure is more than 50% higher than the figure reported for all childbearing women. This suggests that black abortion demand is considerably more responsive to income changes than it is for white women. This finding bears out Kenneth Clark's argument that increases in income cause changes in black personal and family aspirations making abortion increasingly a more acceptable and desirable option. |
| [49] [Mozambique] | How do women in Maputo evaluate method choices and experiences? How do individual women choose whether or not to pursue a home-based abortion method and how might their perspectives vary based on their personal circumstances, prior experiences, the quality of care rendered, and the clinical outcome? What do women and nurses think about participating in clinical abortion research? | Women seeking abortion in Maputo | Interviews with women and nursing professionals | Nurses identified demanding work or travel as perceived barriers to misoprostol use and study participation. Pregnant women who entered the misoprostol study were more likely to be studying and living in rented housing raising the possibility that the monetary reimbursement (US$14) could have played a role in their method choice. Although infrequently mentioned by women, nurses noted that some women voiced concerns regarding potential study involvement. Nurses noted that some women were anxious about possible hidden motives behind the reimbursement. |
| [50] [Norway] | To estimate the benefits of increasing abortion access to teenagers in Oslo | Teenage women in Oslo | Analysis of national data using a difference in difference approach and others | Starting in 1969, teenagers in Oslo had access to abortion services that were generally denied to teenagers in other parts of Norway. Mapping into birth cohorts, this implies that cohorts born between 1951 and 1954 had much better access as teenagers in Oslo than in the rest of the country. This abortion access translated into lower rates of teen fertility, higher age at first birth and a small increase in completed family size. Evidence also shows that greater abortion access led to higher educational attainment, and teen abortion access led to higher labor market attachment at younger ages but lower attachment after about age 35. These findings are consistent with abortion leading to a delay in fertility that increases labor market attachment at younger ages but decreased it later. Finally, early abortion access led to lower teen pregnancy rates and welfare use in the next generation. It is also associated with a more academic high school track and increased college going. Therefore, there appear to be positive spillover effects to the next generation. |
| [51] [Kenya] | To inform improvements in safe abortion and post-abortion family planning (PAFP) services, this study aimed to explore the pathways, decision-making, experiences and preferences of women receiving safe abortion and post-abortion family planning (PAFP) at private clinics in western Kenya | Women who had received an abortion or post-abortion care service at one of nine clinics (n=22) | Semi-structured interviews | Some respondents also struggled to obtain money and had to delay obtaining the service while they gathered funds. However, most respondents viewed the services as good value. The reasons for terminating the pregnancy were most commonly that respondents were still in education or were caring for another young child. |
| [52] [United States] | To describe payment for abortion care before new restrictions among a sample of women receiving first and second trimester abortions | English- and Spanish-speaking women aged 15 and older, with no known fetal anomalies or demise, presenting for abortion care at one of 30 facilities throughout the United States between January 2008 and December 2010 and meeting specific gestational age criteria | Interviews and regression analysis | In a multivariate analysis, living in a state where Medicaid for abortion was available, having Medicaid or private insurance, being at a lower gestational age, and higher income were associated with lower odds of reporting cost as a reason for delay. Out-of-pocket costs for abortion care are substantial for many women, especially at later gestations. There are significant gaps in public and private insurance coverage for abortion. Other organizations, such as abortion funds, were more commonly reported as sources of payment among women with pregnancies beyond the first trimester and that women receiving abortions after 20 weeks were likelier to have had financial assistance from insurance and other organizations than women receiving first trimester abortions. In addition, the findings echo existing research documenting increased travel costs associated with obtaining abortion at later gestational ages. |
| [53] [South Africa] | To explore participants decision-making in abortions, including circumstances of procedure and perceptions of coping. | Black women who had undergone an induced abortion within a period of three months of the study (n=5) | Qualitative interviews | The economic context of four of the five women was significant in the abortion trajectories, including their reasons for obtaining abortions (e.g. not being able to afford to have a child; expense of raising children).  Women also cited a desire to finish their studies or the impact on their employment as reasons for their abortions. |
| [54] [Australia] | To illuminate an important clinical question that had been inaccessible to researchers until the 1970s: What effect did an abortion have on normally rule abiding women? | Women seeking abortion services | Interviews | The most important change reported by the women was an increased capacity to run their own lives. The women discovered that they could make and carry out difficult decisions, that they could alter the course of events and exert their wishes over their destiny. Single, nulliparous women took mothering seriously and had an abortion to avoid becoming inadequate mothers. Abortion was a challenge to the married womens’ sense of themselves as good mothers, and their motives related to good mothering. The working class women had histories of managing tough and challenging life events, and they used the strengths, skills and networks they had established and applied those to the abortion decision. |
| [55][United States] | To understand abortion care in relation to providers of women’s health care | Pregnant people seeking abortions across three clinics in two states in the U.S. Heartland | Qualitative interviews | Case study of Joy [pseudonym] who chose her general provider in anticipation of the costs being cheaper. It was more important, if the costs were the same, for Joy to use her regular clinic as it was “comfortable”. |
| [56] [Latin America] | To summarize the findings of a literature review on women’s experiences with medical abortion in Latin American countries where voluntary abortion is illegal | Studies on women’s experiences with medical abortion in Latin American countries where voluntary abortion is illegal | Literature review | Women perceived medical abortion as safer, more practical and less expensive than other methods. |

1. Ahmed S, Islam A, Khanum PA, Barkat e K. Induced abortion: What's happening in rural Bangladesh. Reproductive Health Matters. 1999;7(14):19-29. doi: <https://doi.org/10.1016/S0968-8080(99)90003-4>.

2. Aiken ARA, Johnson DM, Broussard K, Padron E. Experiences of women in Ireland who accessed abortion by travelling abroad or by using abortion medication at home: a qualitative study. BMJ sexual & reproductive health. 2018. Epub 2018/07/05. doi: 10.1136/bmjsrh-2018-200113. PubMed PMID: 29972360; PubMed Central PMCID: PMCPMC6237647.

3. Aiken ARA, Guthrie KA, Schellekens M, Trussell J, Gomperts R. Barriers to accessing abortion services and perspectives on using mifepristone and misoprostol at home in Great Britain. Contraception. 2018;97(2):177-83. doi: <https://doi.org/10.1016/j.contraception.2017.09.003>.

4. Aiken A, Gomperts R, Trussell J. Experiences and characteristics of women seeking and completing at-home medical termination of pregnancy through online telemedicine in Ireland and Northern Ireland: a population-based analysis. BJOG : an international journal of obstetrics and gynaecology. 2017;124(8):1208-15. Epub 2016/10/18. doi: 10.1111/1471-0528.14401. PubMed PMID: 27748001; PubMed Central PMCID: PMCPMC5393954.

5. Akin A, Kocoglu GO, Akin L. Study Supports the Introduction of Early Medical Abortion in Turkey. Reproductive Health Matters. 2005;13(26):101-9.

6. Almond D, Edlund L, Milligan K. Son Preference and the Persistence of Culture: Evidence from South and East Asian Immigrants to Canada. Population and Development Review. 2013;39(1):75-95.

7. Alouini S, Uzan M, Méningaud JP, Hervé C. Knowledge about contraception in women undergoing repeat voluntary abortions, and means of prevention. European Journal of Obstetrics & Gynecology and Reproductive Biology. 2002;104(1):43-8. doi: <https://doi.org/10.1016/S0301-2115(02)00060-X>.

8. Altshuler AL, Ojanen-Goldsmith A, Blumenthal PD, Freedman LR. A good abortion experience: A qualitative exploration of women's needs and preferences in clinical care. Social Science & Medicine. 2017;191:109-16. doi: <https://doi.org/10.1016/j.socscimed.2017.09.010>.

9. Aniteye P, Mayhew S. Attitudes and Experiences of Women Admitted to Hospital with Abortion Complications in Ghana. African Journal of Reproductive Health / La Revue Africaine de la Santé Reproductive. 2011;15(1):47-55.

10. Appiah-Agyekum NN, Sorkpor C, Ofori-Mensah S. Determinants of abortion decisions among Ghanaian university students. International journal of adolescent medicine and health. 2015;27(1):79-84. Epub 2014/08/26. doi: 10.1515/ijamh-2014-0011. PubMed PMID: 25153553.

11. Bailey PE, Bruno ZV, Bezerra MF, Queiróz I, Oliveira CM, Chen-Mok M. Adolescent pregnancy 1 year later: the effects of abortion vs. motherhood in Northeast Brazil. Journal of Adolescent Health. 2001;29(3):223-32. doi: <https://doi.org/10.1016/S1054-139X(01)00215-4>.

12. Banerjee SK, Andersen K. Exploring the pathways of unsafe abortion in Madhya Pradesh, India. Global Public Health. 2012;7(8):882-96. doi: 10.1080/17441692.2012.702777. PubMed PMID: WOS:000308031400007.

13. Bélanger D, Flynn A. The Persistence of Induced Abortion in Cuba: Exploring the Notion of an "Abortion Culture". Studies in Family Planning. 2009;40(1):13-26.

14. Belton S, Whittaker A. Kathy Pan, sticks and pummelling: Techniques used to induce abortion by Burmese women on the Thai border. Social Science & Medicine. 2007;65(7):1512-23. doi: <https://doi.org/10.1016/j.socscimed.2007.05.046>.

15. Bennett LR. Single women's experiences of premarital pregnancy and induced abortion in Lombok, Eastern Indonesia. Reproductive Health Matters. 2001;9(17):37-43. doi: <https://doi.org/10.1016/S0968-8080(01)90006-0>.

16. Biggs MA, Ralph L, Raifman S, Foster DG, Grossman D. Support for and interest in alternative models of medication abortion provision among a national probability sample of U.S. women. Contraception. 2018. doi: <https://doi.org/10.1016/j.contraception.2018.10.007>.

17. Brack CE, Rochat RW, Bernal OA. It's a Race Against the Clock: A Qualitative Analysis of Barriers to Legal Abortion in Bogot, Colombia. International Perspectives on Sexual and Reproductive Health. 2017;43(4):173-82.

18. Broen AN, Moum T, Bodtker AS, Ekeberg O. Reasons for induced abortion and their relation to women's emotional distress: a prospective, two-year follow-up study. General hospital psychiatry. 2005;27(1):36-43. Epub 2005/02/08. doi: 10.1016/j.genhosppsych.2004.09.009. PubMed PMID: 15694217.

19. Chae S, Desai S, Crowell M, Sedgh G. Reasons why women have induced abortions: a synthesis of findings from 14 countries. Contraception. 2017;96(4):233-41. doi: 10.1016/j.contraception.2017.06.014.

20. Chibber KS, Biggs MA, Roberts SCM, Foster DG. The Role of Intimate Partners in Women's Reasons for Seeking Abortion. Women's Health Issues. 2014;24(1):e131-e8. doi: <https://doi.org/10.1016/j.whi.2013.10.007>.

21. Chunuan S, Kosunvanna S, Sripotchanart W, Lawantra J, Lawantrakul J, Pattrapakdikul U, et al. Characteristics of Abortions in Southern Thailand. Pacific Rim International Journal of Nursing Research. 2012;16(2):97-112.

22. Cockrill K, Weitz TA. Abortion patients' perceptions of abortion regulation. Women's health issues : official publication of the Jacobs Institute of Women's Health. 2010;20(1):12-9. Epub 2010/02/04. doi: 10.1016/j.whi.2009.08.005. PubMed PMID: 20123172.

23. Dennis A, Manski R, Blanchard K. Does Medicaid Coverage Matter? A Qualitative Multi-State Study of Abortion Affordability for Low-income Women. Journal of Health Care for the Poor & Underserved. 2014;25(4):1571-85. doi: 10.1353/hpu.2014.0151.

24. Díaz-Olavarrieta C, Cravioto VM, Villalobos A, Deeb-Sossa N, García L, García SG. Mexico City’s Legal Abortion Program: health workers’ experiences. Revista Panamericana de Salud Publica. 2012;32(6):399-404.

25. Fergusson DM, Boden JM, Horwood LJ. Abortion among young women and subsequent life outcomes. Perspectives on Sexual & Reproductive Health. 2007;39(1):6-12.

26. Fernandez Lopez MI, Carrillo Navarro F, Leal Hernandez M, Carrillo Canaveras I, Carrillo Navarro E, Lozano Sanchez ML, et al. [An approach to abortion and immigration in Spain. The social economic value of the human life]. Cuadernos de bioetica : revista oficial de la Asociacion Espanola de Bioetica y Etica Medica. 2010;21(73):313-26. Epub 2010/11/26. PubMed PMID: 21090843.

27. Finer LB, Frohwirth LF, Dauphinee LA, Singh S, Moore AM. Reasons U.S. Women Have Abortions: Quantitative and Qualitative Perspectives. Perspectives on Sexual and Reproductive Health. 2005;37(3):110-8.

28. Flory F, Manouana M, Janky E, Kadhel P. Caractéristiques sociodémographiques et médicales des interruptions volontaires de grossesse des mineures en Guadeloupe. Gynécologie Obstétrique & Fertilité. 2014;42(4):240-5. doi: <https://doi.org/10.1016/j.gyobfe.2013.05.001>.

29. Foster DG, Kimport K. Who Seeks Abortions at or After 20 Weeks? Perspectives on Sexual & Reproductive Health. 2013;45(4):210-8. doi: 10.1363/4521013.

30. Ganatra B, Hirve S. Induced Abortions Among Adolescent Women in Rural Maharashtra, India. Reproductive Health Matters. 2002;10(19):76-85. doi: <https://doi.org/10.1016/S0968-8080(02)00016-2>.

31. Gibb S, Donaldson C, Henshaw R. Assessing strength of preference for abortion method using 'willingness to pay': a useful research technique for measuring values. Journal of Advanced Nursing. 1998;27(1):30-6. doi: 10.1046/j.1365-2648.1998.00473.x.

32. Gresh A, Maharaj P. A qualitative assessment of the acceptability and potential demand for medical abortion among university students in Durban, South Africa. The European journal of contraception & reproductive health care : the official journal of the European Society of Contraception. 2011;16(2):67-75. Epub 2011/01/26. doi: 10.3109/13625187.2010.546534. PubMed PMID: 21261553.

33. Gruber J, Levine P, Staiger D. Abortion Legalization and Child Living Circumstances: Who is the 'Marginal Child?'. 1997.

34. Howie FL, Henshaw RC, Naji SA, Russell IT, Templeton AA. Medical abortion or vacuum aspiration? Two year follow up of a patient preference trial. British journal of obstetrics and gynaecology. 1997;104(7):829-33. Epub 1997/07/01. PubMed PMID: 9236649.

35. Hulme-Chambers A, Temple-Smith M, Davidson A, Coelli L, Orr C, Tomnay JE. Australian women’s experiences of a rural medical termination of pregnancy service: A qualitative study. Sexual & Reproductive Healthcare. 2018;15:23-7. doi: <https://doi.org/10.1016/j.srhc.2017.11.008>.

36. Hussey LS. Is Welfare Pro-life? Assistance Programs, Abortion, and the Moderating Role of States. Soc Serv Rev. 2011;85(1):75-107. doi: 10.1086/659227. PubMed PMID: WOS:000289619400003.

37. Izugbara CO, Ukwayi JK. The clientele of traditional birth homes in rural southeastern Nigeria. Health Care for Women International. 2003;24(3):177-92. PubMed PMID: 106843171. Language: English. Entry Date: 20030627. Revision Date: 20150818. Publication Type: Journal Article.

38. Jejeebhoy SJ, Kalyanwala S, Zavier AJF, Kumar R, Jha N. Experience seeking abortion among unmarried young women in Bihar and Jharkhand, India: delays and disadvantages. Reproductive Health Matters. 2010;18(35):163-74. doi: <https://doi.org/10.1016/S0968-8080(10)35504-2>.

39. Jerman J, Frohwirth L, Kavanaugh ML, Blades N. Barriers to Abortion Care and Their Consequences For Patients Traveling for Services: Qualitative Findings from Two States. Perspectives on Sexual & Reproductive Health. 2017;49(2):95-102. doi: 10.1363/psrh.12024.

40. Johansson A, Le Thi Nham T, Lap Nt, Sundstrom K. Abortion in Context: Women's Experience in Two Villages in Thai Binh Province, Vietnam. International Family Planning Perspectives. 1996;22(3):103-7. doi: 10.2307/2950750.

41. Johnson BR, Horga M, Andronache L. Women's perspectives on abortion in Romania. Social Science & Medicine. 1996;42(4):521-30. doi: <https://doi.org/10.1016/0277-9536(95)00175-1>.

42. Jones RK, Upadhyay UD, Weitz TA. At What Cost? Payment for Abortion Care by U.S. Women. Women's Health Issues. 2013;23(3):e173-8. doi: 10.1016/j.whi.2013.03.001.

43. Kalyanwala S, Zavier AJF, Jejeebhoy S, Kumar R. Abortion Experiences of Unmarried Young Women In India: Evidence from a Facility-Based Study In Bihar and Jharkhand. International Perspectives on Sexual and Reproductive Health. 2010;36(2):62-71.

44. Kinoti SN, Gaffikin L, Benson J. How research can affect policy and programme advocacy: example from a three-country study on abortion complications in sub-Saharan Africa. East African medical journal. 2004;81(2):63-70. Epub 2004/05/06. PubMed PMID: 15125088.

45. Loeber O, Wijsen C. Factors Influencing the Percentage of Second Trimester Abortions in the Netherlands. Reproductive Health Matters. 2008;16(31, Supplement):30-6. doi: <https://doi.org/10.1016/S0968-8080(08)31377-9>.

46. Margo J, McCloskey L, Gupte G, Zurek M, Bhakta S, Feinberg E. Women's Pathways to Abortion Care in South Carolina: A Qualitative Study of Obstacles and Supports. Perspectives on Sexual & Reproductive Health. 2016;48(4):199-207. doi: 10.1363/psrh.12006.

47. Medoff MH. An Estimate of Teenage Abortion Demand. Journal of Socio-Economics. 1999;28(2):175-84. doi: <http://www.sciencedirect.com/science/journal/10535357>. PubMed PMID: 0606267.

48. Medoff MH. Black Abortion Demand. Review of Black Political Economy. 2000;28(1):29-36. doi: <https://link.springer.com/journal/volumesAndIssues/12114>. PubMed PMID: 0592499.

49. Mitchell EM, Kwizera A, Usta M, Gebreselassie H. Choosing early pregnancy termination methods in Urban Mozambique. Social Science & Medicine. 2010;71(1):62-70. doi: 10.1016/j.socscimed.2010.03.025. PubMed PMID: 105053222. Language: English. Entry Date: 20100813. Revision Date: 20150711. Publication Type: Journal Article.

50. Molland E. Benefits from Delay? The Effect of Abortion Availability on Young Women and Their Children. Labour Economics. 2016;43:6-28. doi: <http://www.sciencedirect.com/science/journal/09275371>. PubMed PMID: 1596811.

51. Penfold S, Wendot S, Nafula I, Footman K. A qualitative study of safe abortion and post-abortion family planning service experiences of women attending private facilities in Kenya. Reproductive Health. 2018;15(1):N.PAG-N.PAG. doi: 10.1186/s12978-018-0509-4. PubMed PMID: 129271442. Language: English. Entry Date: 20180426. Revision Date: 20190107. Publication Type: Article.

52. Roberts SCM, Gould H, Kimport K, Weitz TA, Foster DG. Out-of-Pocket Costs and Insurance Coverage for Abortion in the United States. Women's Health Issues. 2014;24(2):e211-8. doi: 10.1016/j.whi.2014.01.003.

53. Suffla S. Experiences of induced abortion among a group of South African women. South African journal of psychology = Suid-Afrikaanse tydskrif vir sielkunde. 1997;27(4):214-22. Epub 2002/09/27. PubMed PMID: 12321538.

54. Wainer J. Abortion and the struggle to be good in the 1970s. Australian & New Zealand Journal of Psychiatry. 2008;42(1):30-7.

55. Weitz TA, Cockrill K. Abortion clinic patients' opinions about obtaining abortions from general women's health care providers. Patient Education & Counseling. 2010;81(3):409-14. doi: 10.1016/j.pec.2010.09.003. PubMed PMID: 104963200. Language: English. Entry Date: 20110401. Revision Date: 20150711. Publication Type: Journal Article.

56. Zamberlin N, Romero M, Ramos S. Latin American women's experiences with medical abortion in settings where abortion is legally restricted. Reprod Health. 2012;9(1):34. Epub 2012/12/25. doi: 10.1186/1742-4755-9-34. PubMed PMID: 23259660; PubMed Central PMCID: PMCPMC3557184.
