# Supplementary material for: The microeconomics of abortion: A scoping review and analysis of the economic consequences for abortion care-seekers
Source: PLoS One. 2021 Jun 9;16(6):e0252005. doi: 10.1371/journal.pone.0252005 (PMC8189560; doi:10.1371/journal.pone.0252005)
Supplement: Attachment — Submitted filename: Micro econ response to reviewers 271120.docx [file pone.0252005.s005.docx]

Below we respond in detail to the reviewers’ comments and editorial requirements

| Editor / Reviewer comment | Manuscript changes / changes pending |
| --- | --- |
| Editorial |  |
| Please ensure that your manuscript meets PLOS ONE's style requirements, including those for file naming^[1]^ | All files have been reviewed to meet PLOS ONE style requirements. |
| Please include your tables as part of your main manuscript and remove the individual files. Please note that supplementary tables (should remain/ be uploaded) as separate "supporting information" files. | Tables have now been included in the manuscript [supplementary tables remain in supporting information files] |
| Funding: Please clarify the sources of funding (financial or material support) for your study. List the grants or organizations that supported your study, including funding received from your institution. State what role the funders took in the study. If the funders had no role in your study, please state: “The funders had no role in study design, data collection and analysis, decision to publish, or preparation of the manuscript.” If any authors received a salary from any of your funders, please state which authors and which funders. If you did not receive any funding for this study, please state: “The authors received no specific funding for this work.” Please include your amended statements within your cover letter; we will change the online submission form on your behalf. | *This work was supported by the Netherlands Ministry of Foreign Affairs, activity number 28438. This funder had no role in the design and development of the manuscript or the decision to publish.* |
| Please complete your Competing Interests on the online submission form to state any Competing Interests. If you have no competing interests, please state "The authors have declared that no competing interests exist.", as detailed online in our guide for authors at [http://journals.plos.org/plosone/s/submit-now](about:blank) This information should be included in your cover letter; we will change the online submission form on your behalf. | *The authors have declared that no competing interests exist.* |
| Please amend your manuscript to include your abstract after the title page. | Amended – abstract included after title page |
| Please include captions for your Supporting Information files at the end of your manuscript, and update any in-text citations to match accordingly. Please see our Supporting Information guidelines for more information: [http://journals.plos.org/plosone/s/supporting-information](about:blank) | Captions for Supporting Information files have been placed at the end of the manuscript |
| Reviewer #1 |  |
| I think the greatest value of the manuscript is when it draws universal truths from this scoping review to demonstrate how similar barriers and facilitators are across the world. It would be incredibly valuable if they were able to provide guidance on how to standardize data collection on micro-economic costs; even if only to create discrete categories of types of costs for future research activities. | Thank you. Whilst developing guidance on how to standardize data collection on micro-economic costs is beyond the scope of our review, we agree that this would be a valuable addition to the field. We have amended the discussion to reflect this. Line 571-579. |
| I wonder if the authors have bitten off more than they can chew with making the scoping review so broad. I would suggest that they separate out discrete portions of this analysis rather than trying to publish it as one scoping review covering all micro-level economic impacts. Another option would be for them to narrow their search terms more. | We have substantially streamlined our revised manuscript in response to this and other comments from the Reviewer for which, thanks. As a scoping review, our aim is to be deliberately wide, including in our framing of economics – focusing on costs, impacts, benefits and values – rather than, for example, financial costs alone. We have chosen to retain the broad scope of this review rather than to separate out discrete portions of this analysis in order to present a holistic picture of the microeconomics of abortion. |
| The manuscript should say up front what the period of publication years was that were included in this scoping review, and languages included, not just leave that information in an appendix. | This information is included in Table 1, which has now been inserted into the main text. Table 1 – PICOTS criteria used in the scoping review [PICOTS = Populations, Interventions, Control, Outcomes, Timeframe and Setting] – specifies these aspects of the scoping review. We had included mention of the included language [English, French, Spanish, German and Dutch] in our limitations in the original manuscript. We have added an additional sentence in the methodology to make this point earlier in the manuscript [Line 133] “We included items in English, French, Spanish, German and Dutch.” |
| Why were the articles recommended by experts not picked up in the searches? By adding in those resources, it seems like the methodology is no longer replicable. | Searches for, and identification of, relevant literature are dependent to some extent on the quality of the indexing [keywords, title, abstract] of the original item in the database / website. Expert recommendations for scoping reviews are therefore a standard tool^[2]^ to identify work that our replicable search strategy might have missed, including more recent work that might not have been indexed at the time of our search. |
| Is the framework of separating costs, impacts, values and benefits from elsewhere or is this a framework that the authors have created? | The framework was developed by us, to reflect our focus on the economics of abortion, rather than just the finances of abortion. Because economics (like eg: sociology) focuses on behaviours as well as money, the goal of our framework was to include outcomes – negative or positive - that went beyond financial outcomes as measured by cost. We have added text to the paper: Line 87 “The framework was developed by the authors to reflect our focus on the economics of abortion, rather than just the finances of abortion. As economics (like sociology) focuses on behaviours as well as money, the goal of our framework is to include outcomes – negative or positive - that go beyond financial outcomes as measured in monetary terms.” |
| How did the authors treat the costs of abortion attempts likely to be ineffective? Quinine is not an effective abortifacient, but it’s presented as a less expensive abortion method. | Our scoping review is focused on all abortion-related care, irrespective of its effectiveness and safety. We are centring what people do with respect to seeking abortion-related care – including ineffective actions undertaken to induce an abortion. We have added two sentences along these lines to the paper. Line 115 “Our scoping review is focused on all abortion-related care, irrespective of its effectiveness and safety. We are centering what people do with respect to seeking abortion-related care – including ineffective actions undertaken to induce an abortion.“ |
| In the paragraph on evidence from countries where abortion is theoretically free but in fact women have to pay, India would be a valuable country to also include. | This para has been substantially revised, with an example from India moved from elsewhere (discussion) to add to the examples of countries where abortion is theoretically free. |
| Are “unofficial fees” bribes? | Text amended from “unofficial fees” to unofficial *payments*”. Evidence from items reporting unofficial payments [made either in cash or as grifts] cannot be only described as bribes. The language around corruption in healthcare provision is diverse – and includes but is not limited bribery, extortion, informal payments (Garcia, 2019^[3]^). We have selected to use “unofficial payment” in order to capture the diversity of such costs when identified in included items. Sometimes unofficial payment is demanded [and care withheld without payment] and sometimes unofficial payment is not demanded per se but expected [and paid to receive treatment – or eg: faster or preferential treatment] |
| When the authors present the comparator costs, sometimes they include the US$ equivalent and sometimes they don’t. It would be good to be consistent. | We present the comparator costs when they are provided by the original item – comparator costs are not subsequently calculated by us. In our writing we suggest that inclusion of comparator costs [whether absolute or relative] in future research would enhance the evidence base on the economics of abortion. We have clarified this in the manuscript [Line 203] “We present the comparator costs when they are provided by the original study.” |
| Can the Results section be given a more signposted organization, e.g. with subheadings, within the three major sections? | We agree and sub-headings have been introduced throughout |
| The paper is extremely long. An analysis that makes the authors categorize the types of evidence and conclusions would allow them to cut down verbosity and summarize the results more succinctly rather than so much explication. When the findings are the same across different contexts, they don’t need a sentence for every country (e.g. lines 375-381). | Throughout the paper, substantial reductions in the detailed descriptions of individual items have been made [and this is reflected in multiple responses below]. The supplementary tables provide detailed information about the types of evidence and key results for each item. |
| I think that the “types” of evidence considered is deserving of more explication, i.e. author extrapolations of the meaning of costs on women’s lives is also considered data, what the authors “infer” (lines 504 and 508) (or is it information that the authors of the cited pieces inferred? It’s not clear), interpretations (line 511). Should hypothesized cost impacts (pg. 12, lines 265-267) be given the same weight as respondents identifying cost as a barrier? It seems like that shouldn’t have made it into the scoping review as it’s not a finding of the study, just a hypothesis by the authors. | As a scoping review – which explicitly excludes a quality assessment of included items – we do not weight the evidence according to the type of data presented by authors in an included item. Where authors of an included item have inferred – on the basis of their evidence – an economic outcome [cost, impact, benefit or value], this is included – but it is made explicit that this is the inference or hypothesis of the item’s author(s). Text added to the manuscript Line 136 “Therefore, as a scoping review that explicitly excludes a quality assessment of included studies, we do not “weigh” the evidence presented by authors in an included item. Where authors of an included study inferred an economic outcome [cost, impact, benefit or value] on the basis of their evidence, the findings in this manuscript explicitly state that this inference or hypothesis belongs to the author(s) of the included study.” |
| Pg.8, lines 173-175: Why are brokers needed if the women could pay with a credit/debit card? Wouldn’t a broker help women who don’t have a credit/debit card? | Text amended to “In Kenya, patients unable to access a facility’s required mode of payment (eg: mobile money or the use of a credit/debit card) used brokers who charged a fee,” |
| Pg. 8, lines 183-185: Describing how women feel about the money they obtained feels beyond the scope of “costs,” and belongs with “impacts.” | Text deleted from Costs |
| Pg. 9, line 190: costs of spontaneous abortion? That would be US$ 0, unless the woman went for care. Do they mean “PAC for induced abortion vs. spontaneous abortion”? The next sentence has the same problem as PAC doesn’t just mean care for induced abortions. And see again pg. 14, line 318, and throughout. | Text amended to specify whether PAC for induced abortion or spontaneous abortion  “and PAC for induced abortion compared to PAC for spontaneous abortion (Henshaw, Adewole et al. 2008, Banerjee, Andersen et al. 2012, Ilboudo, Greco et al. 2015). Two patterns emerge: micro-level costs for PAC for induced abortion compared to care for a spontaneous abortion are significantly higher, often as a result of complex care-seeking trajectories due to the need for secrecy in restrictive settings; and, the costs of PAC for induced abortion are higher than those of safe abortion (Leone, Coast et al. 2016).” |
| Traveling to another country for an abortion is really going to generate other costs than paying for an abortion at home; it shouldn’t be treated in the same paragraph as just another cost associated with the abortion. It should be introduced as an option that some women seek who reside in countries where they can’t legally get an abortion. And while we think it’s only the wealthiest who get abortions this way, in fact more than half of women found it “difficult or very difficult” to cover the cost of travel, which is an interesting finding likely related to the fact that wealth is not equal to the money women themselves can actually control. | Agree that example referring to travel costs is incorrectly included in this paragraph; text removed. |
| --Pg. 10, line 210: “The inadequacies of data…” is an observation that belongs in the Discussion. | Content revised and moved to discussion |
| --Pg. 10, lines 212-213: It bears pointing out by the authors that they are looking at the costs and delays for both induced abortion and postabortion care. In its purest form, the three delays should only apply to postabortion care. --Pg. 10, line 224: Was raising money a delay in deciding or was delay in deciding a reason for winding up with a second-trimester abortion, which of course costs more money? I presume the latter, and not the way the text is currently written. --Pg. 10, lines 218-222: belong in the second delay, not the first. | We have revised this section and placed mention of the 3 Delays Model into the discussion. |
| --Pg. 10, line 230: after “*Delays in receiving abortion-related care,”* a transition sentence is needed to explain the Bangladesh example. That transition sentence could explain that a lot of abortion-related care women seek is not allopathic, and therefore may not resolve their complications. | Example removed as part of streamlining overall text in response to separate comments about the level of detail reported about individual items. |
| --Pg. 11, line 248: other considerations, such as what is considered more “discreet” should be mentioned in the Discussion as additional factors influencing abortion method choice that are not influenced by cost. To mix them in there dilutes and distracts from the focus on cost, and this is not a review of all factors women take into account when determining how to conduct an abortion. Further in the same paragraph, lines 256: “limited appointment availability, and desire to avoid protestors picketing” falls into this same category as well, as does pg. 13, line 285’s reference to abortion access, and a bit further along, references to secrecy concerns, and a tendency to delay seeking services and an unwillingness of qualified providers to help them. Again, see lines 346-348. I would argue that the other concerns beyond financial, are not part of the Results of this paper. | Text amended to remove reference to factors (discreteness, protestors, unwilling providers, secrecy etc.) not related to economic costs, and Discussion revised to include mention of these additional factors. |
| --Pg. 12, lines 273-275: The authors should make clear that consent by an adult is not required specifically because she’s getting abortion-related care, it’s because she’s a minor getting *any* medical care. | Text amended to clarify [additions in italics]: In India, because the law requires a guardian’s consent for *all medical care– including abortion services -* for individuals aged below 18 years… |
| --Pg. 12, line 278: 11-13 year olds in the US “fully employed”? They are not going to be employed as it’s prohibited by law. The authors of this article don’t make the argument that it’s likely due to abuse and that’s also why abortions are delayed? | Text amended to “…the authors suggest that this age group has ‘significant difficulty’ acquiring the funds needed for abortion procedures”. The authors (Ely et al 2017b) do not deal with or mention the [potential] role of abuse in abortion care-seeking in this article. |
| --Pg. 17, line 381-383: Do the authors distinguish in the impact of employment/work/income between women getting safe abortions vs. unsafe abortions? Or is it the impact of even having a safe abortion in these countries that the referenced article is about? | Responding to other comments to reduce the detail of individual studies, this text has been removed as part of a streamlining of this section. |
| --Pg. 17, lines 386, 388—why not summarize the findings from Reference 13 here? It’s a noteworthy omission. | In response to other comments about the level of detail in the manuscript, the text at this location has been substantially reduced. |
| --Pg. 17, lines 398-398: “Women compared…raising a child” shouldn’t be included there but could be included in the description of what women are taking into account when incurring impacts of the abortion. It belongs under “Values and benefits evidence derived from women’s reasons for having an abortion.” | This study was already included under benefits and values. Text removed from Economic impacts section. |
| Pg. 18, lines 412-414: How does that fit under the category of “Studies comparing the economic impacts of different types of abortion care-seeking?” | Text deleted. |
| Pg. 18, lines 415-418: not enough information is given about the intervention to allow the reader to understand how this intervention worked and how the evidence presented demonstrates economic impacts. | We were unable to identify exactly what this comment referred to. |
| Pg. 19, line 426: “Non-Parenting Turnaways” were not identified in the set up of the discrete respondent categories. | Thank you for highlighting this. Our revised text now includes the fourth category “Non-Parenting Turnaways (included Turnaways who subsequently had an abortion elsewhere, reported that they had miscarried, or placed the child for adoption)”. |
| Pg. 20, line 457: “that the clinic was the closest location…” needs greater clarity, i.e. “that the clinic which was the closest only provided medical abortion” | Responding to other comments to reduce the detail of individual studies, this text has been removed as part of a streamlining of this section. |
| Pg. 20, lines 462-466: What is the “benefits and values” take-away here? That benefits and values are ranked differently according to people who value “importance of choice” and their social class, and that financial status doesn’t predict how people value benefits and values? It’s a lot of work for the reader to come to that conclusion from the text provided. | Responding to other comments to reduce the detail of individual studies, this text has been removed as part of a streamlining of this section. |
| Pg. 20, line 469: “experience inequity” compared to whom? | Text amended to “…suggests that women with few economic and social resources valued the lower costs of telemedicine compared to having to travel for an abortion”. |
| Pg. 21, lines 473-476: what are readers supposed to take away from that? | Responding to other comments to reduce the detail of individual studies, this text has been removed as part of a streamlining of this section. |
| Pg. 22, lines 513-515: This text belongs in the Discussion as an evidence gap under “areas for future research.” | Removed from the flagged page and included in the discussion. |
| Pg. 23, line 531: This article doesn’t go into decision-making about whether or not to have an abortion, but about when, where and what kind of care to get. Perhaps make that explicit? | Text amended to reflect that economic factors are implicated in decision-making about, and delays to, decision-making about abortion-related care. |
| Pg. 24, lines 542-544: Should be moved to an evidence gap under “areas for future research.” (It is there again in lines 554-556—so could just be deleted from 542-544.) | Text amended to consolidate all suggestions of areas for future research. |
| Pg. 24, lines 545-546: “the ways in which perceptions of economic factors…affect abortion-related care-seeking and its timing” is an incomplete thought. | Line 555 text amended to “We know very little about the ways in which economic factors (including perceptions of economic factors) intersect with concepts of risk/safety and quality of care to affect abortion-related care-seeking and its timing.“ |
| Pg. 24, line 552: Are we now at “areas for future research”? It could be signposted more clearly. | Text amended to more clearly signal discussion of evidence gaps and areas for future research |
| Pg. 25, line 568: Phone-based payment systems were never discussed in the paper, nor was there any evidence that some providers *only* accept debit/credit cards and not cash. | This sentence was included in order to suggest some of the ways in which the modalities of financial payments might change, with potential implications. One of the included papers (Mutua et al, 2018) details how mobile money payments are becoming increasingly critical to access abortion-related care. In the same paper, some providers were reported as not accepting cash [only cards or mobile money]. |
| Another idea brought forth for the first time in the Discussion is the conditionality of care—lines 570-575 belong in the Results. | This sentence was moved to the results section. |
| --There is an overuse of additional clauses between en-dashes. Furthermore, sometimes the dashes are hyphens (which is incorrect) and sometimes they are en-dashes. For example, see lines 389-391: three en-dashes in one sentence! | En-dashes checked throughout, including this specific location, and amended. . |
| --Sometimes it’s USA and sometimes it’s US. Best to stay consistent. | The text now uses USA consistently. |
| --abortion-related care—it should be hyphenated. | “abortion-related” used consistently throughout the revised manuscript |
| --Pg. 3, lines 51-64: discuss the costs, impacts, values and benefits in the same order as they are listed. (Currently, benefits are discussed before values.) Pg. 6, line 123, the order goes to “costs, impacts, benefits and value.” Please keep them in the same order throughout as it’s already a challenge to keep straight the differences between each domain. Since you wind up merging these last two categories anyway, perhaps begin by just having three domains—it’s less taxing for your reader. | Text changed so that category order is presented consistently as costs, impacts, benefits and values. Because we originally searched and coded for the four categories, we retain them in the methodology section, and combine values and benefits in the analytic section. |
| --Perhaps spell out the acronym TIAB when it’s used the first time? | Text amended to explain TIAB as title and abstract. |
| --Pg. 6, line 121: “ort” should be “or” | Thanks for spotting this typo. Changed to “or”. |
| --This is a hard sentence to grasp: “Study populations were most likely to be based on abortion care-seeker status.” | Text amended to: Study populations were most likely to be based on abortion care-seeker status *rather than other categories (eg: age, race/ethnicity, geographical location)*. |
| --Pg. 9, line 201: to be consistent with the writing on line 196 and 200, the authors should a) use a single or a double quotation mark consistently and b) consistently use them around the categories, e.g. somewhat or very difficult. | Used consistently throughout in revised manuscript using single ‘’ for quotes and double “” for our emphases / concepts |
| --Pg. 9, line 43: A new paragraph should begin with “The use of non-financial measures to assess…” | New paragraph inserted. |
| --Pg. 10, line 227: Is NI supposed to be a country? | Yes. NI previously defined in original manuscript as Northern Ireland [line xxx]. Text now amended to spell out Northern Ireland in the relevant places, and we have replaced RoI with the Republic of Ireland. |
| --Some of the citations include too much detail, e.g. language, entry date, revision date, publication type. These pieces of information are not generally included in references. And the journal names are not uniformly capitalized, e.g. reference #7. | Citations have been checked and amended |
| --Pg. 12, line 281: “abortions” instead of “abortion” connotes that adolescents are having multiple abortions. | Sentence within which “abortions” occurred has been amended; “abortions” removed. |
| --Pg. 13, line 290: “in” should be inserted before listing the three countries, and again on line 292. | “in” inserted in both places. |
| --Pg. 14, line 314 and again 321: “Studies that evidence” is awkward. See also line 374: “Studies evidencing” and throughout. | “evidence” replaced with “demonstrate”, “show”, “identify”, and “explore”. |
| --Pg. 15, line 333 and 334: “their” doesn’t belong if the structure is to be kept parallel within the list. | Text amended to: lowered consumption, increased work or give up jobs |
| --Pg. 16, line 337: hyphenate “low-income”? | Hyphen inserted. |
| --Pg. 16, line 343: Perhaps replace “Medicaid” with something that specifies it’s public health insurance? Readers may not be familiar with what it is. Similarly, I wonder if a greater explanation of TRAP laws is in order. | Medicaid clarified with “public health insurance” at first use. Text inserted to clarify TRAP laws [new text in italics]: Evidence from the USA shows how state-level variation in changing Targeted Regulation of Abortion Provider (TRAP) laws *– the primary purpose of which is to limit abortion access -* can have differential cost implications across multiple intersections. |
| --Pg. 16, line 373: “retrospectively” should be “retrospective” | Changed to “retrospective”. |
| --Pg. 16, line 376: remove “on” | “on” removed. |
| --Pg. 17, line 411: remove “)” | Unable to work out what this is referring to in the m/s |
| --Pg. 22, lines 495-497: This should not be a stand-alone paragraph. | Merged with preceding paragraph. |
| --Pg. 22, line 504: an en-dash needed after “wishes.” | En-dash added. |
| --Pg. 24, line 549: “with” should be “there are” | “with” replaced with “there are”. |
| --Pg. 25, line 566: “the” missing between “that” and “changing” | “the” inserted. |
| Overuse of the word “lacuna/lacunae” | One instance of lacuna replaced with “evidence gap”. |
| --Pg. 26, line 592: “important” should be “importance” | “important” changed to “importance”. |
| Reviewer #2 |  |
| “it would be desirable to conduct an independent research, based on your own research design, in order to quantitatively find out what makes abortion difficult.” | No changes made as this suggestion goes well beyond the scope of our study. |

1. <https://journals.plos.org/plosone/s/file?id=wjVg/PLOSOne_formatting_sample_main_body.pdf> <https://journals.plos.org/plosone/s/file?id=ba62/PLOSOne_formatting_sample_title_authors_affiliations.pdf>
2. [*https://training.cochrane.org/handbook/current/chapter-04*](https://training.cochrane.org/handbook/current/chapter-04)
3. Garcia, P. J. (2019) Corruption in global health: the open secret. The Lancet. <https://doi.org/10.1016/S0140-6736(19)32527-9>
