# Supplementary material for: The microeconomics of abortion: A scoping review and analysis of the economic consequences for abortion care-seekers
Source: PLoS One. 2021 Jun 9;16(6):e0252005. doi: 10.1371/journal.pone.0252005 (PMC8189560; doi:10.1371/journal.pone.0252005)
Supplement: Attachment — Submitted filename: Micro econ RR2 response document 170421.docx [file pone.0252005.s006.docx]

| Reviewer | Response |
| --- | --- |
| there’s too much in this one manuscript, and I would specifically remove (or seriously winnow) the section on “benefits or values” as a lot of the information described there does not fall into the economic sphere, including relationship issues, stigma, and identity maintenance. If the authors insist on keeping this in the paper, they should make the link between these topics and microeconomics much clearer. | We have retained this section due to its relevance to our overall methodology and key themes. However, we did winnow the section as suggested by the reviewer. To further address the reviewer’s concern that this section does not fall in the economic sphere, we added the following text to the beginning of the section: “Microeconomic benefits (advantages) and values (importance, worth, welfare gains, utility) include diverse factors of intrinsic worth at the individual level”. The examples and citations that we retained are facets of people’s lives that affect their well-being, autonomy, and socioeconomic status. |
| I think this conclusion is very important: “Although relatively few micro-level studies are defined explicitly by their authors or their methodology as “economic” studies, our review shows that there is a wealth of economically relevant information that can be gleaned from the evidence base.” | Thank you |
| I also find valuable their specification in research gaps presented in the Discussion. | Thank you |
| They are still overusing the en-dash on pg. 2 and 12, and they’re still mixing hyphens with en-dashes (lines 80-81, line 190, line 275, line 308, lines 331-332, lines 481-482, lines 491-492). | Amended throughout |
| Line 102: “(PRISMA)” acronym not necessary because it appears on line 103. | “(PRISMA)” deleted |
| Line 136: why “simultaneously”? | “simultaneously” deleted |
| Lines 156-159: Another reason for the dominance of studies from the USA is the languages the authors used in which to conduct their search. | Text amended to: “This dominance of the USA in studies of abortion reflects political attention, data availability, the institutional affiliation of authors, the location of funding and other resources for conducting studies, and our search strategy languages.” |
| Line 170: “three-quarters” | Amended to: “three-quarters” |
| Line 173: Still hard to know what is meant by “abortion care-seeker status”—this is a term that the author team is clearly comfortable with but which is not intuitive to the reader. How about, “Study populations were most often whoever was seeking an abortion.” (I don’t even think they need the text “rather than…”) | Text amended to: “Study populations were most likely to be based on an individual’s status as someone seeking abortion care.”. Table 4 also updated to reflect this change. |
| Line 217: “the” missing before “pregnancy outcome.” | “the” inserted |
| Lines 257-258: Stating that the collection of non-financial measures are important is a recommendation, and not a result, and should be moved to the Discussion. | The point about not knowing costs has been retained, but moved to the start of the section on costs.  The recommendation about the importance of non-financial measures is now incorporated into the Discussion. |
| Line 270: insert “any” before “induced abortion” | “any” inserted |
| Line 283: the word “interactions” is not the right word here. | Text amended to: “In the USA, health insurance processes and coverage….” |
| Line 287: what is “an advocacy service”? | Text amended to: “reproductive rights advocacy organisation” |
| Lines 299-230: “women reported various reasons for not using surgical abortion services…because of the cost”? | Text amended to: “In Australia, women reported various reasons for not using surgical abortion services, despite the close proximity of services, including cost” |
| Lines 306-307: “Younger ages may mean higher costs charged for abortion-related services”—this is unnecessarily obtuse. | Text amended to: “Younger people may be charged higher rates for abortion-related services than older people.“ |
| Line 335: Not sure that the word “interventions” is the correct one here; it sounds as though the section would be talking about things like “The Justice Fund” in the USA which was a time-delimited experiment which paid for period for women’s abortions. How about “Social support” or “Charitable accommodations”? | We have decided to retain “interventions” – an intervention as the act of intervening in a situation. Because it is a more generic term that does not specify the type or form of the intervention. The reviewer’s suggestions are more specific and do not capture the broad range of interventions that are described in this section. Although in much economics literature “interventions” has become synonymous with policies and programmes designed to change behaviours, its use here is generic – the act of intervening. |
| Line 361: It’s not the fact that there are a limited number of studies which demonstrates the power of prospective research designs, but rather “There are a limited number of studies which use a prospective design; they demonstrate the potential power to understand how the overall costs…” | Text amended to: “There are a limited number of studies which use a prospective design; they demonstrate the potential power to understand how the overall costs of abortion-related care evolve over time.” |
| Line 372 and the rest of the paragraph, and the paragraph which follows about TRAP laws: although it is possible to cross-reference, the authors should specify in the text geographically where the evidence is coming from. | “USA” inserted |
| Line 394: “prescriptions” for medication abortion? “medication” ◊medication abortion? | Text amended to: “A review of studies in Latin American found that requirements of prescriptions for medical abortion are a barrier that encourage use of informal, often more costly, sources of medical abortion” |
| Lines 400-402: the references are not formatted properly, and there’s a gratuitous period and space after [100] | References amended, thanks for spotting this. |
| Line 450: “in” should be “on” | Amended to “on” |
| Line 501-502: That text belongs at the beginning of the section. | Text (amended) moved to the start of the section |
| Line 539: Indent missing. | Indent added |
|  |  |
|  |  |
|  |  |
